# Feasibility of Specular Reflection Imaging for Extraction of Neck Vessel Pressure Waveforms

**DOI:** 10.3389/fbioe.2022.830231

**Published:** 2022-03-21

**Authors:** Gennadi Saiko, Timothy Burton, Alexandre Douplik

**Affiliations:** ^1^ Photonics Group, Department of Physics, Faculty of Science, Ryerson University, Toronto, ON, Canada; ^2^ Photonics Group, Department of Biomedical Engineering, Faculty of Science, Ryerson University, Toronto, ON, Canada; ^3^ Institute of Biomedical Engineering Science and Technology (iBEST), Keenan Research Centre of the Li Ka Shing (LKS) Knowledge Institute, St. Michael Hospital, Toronto, ON, Canada

**Keywords:** imaging, contactless, non-invasive, cardiovascular, diagnostics, heart failure

## Abstract

Cardiovascular disease (CVD) is a leading cause of death worldwide and was responsible for 31% of all deaths in 2015. Changes in fluid pressures within the vessels of the circulatory system reflect the mechanical function of the heart. The jugular venous (JV) pulse waveform is an important clinical sign for assessing cardiac function. However, technology able to aid evaluation and interpretation are currently lacking. The goal of the current study was to develop a remote monitoring tool that aid clinicians in robust measurements of JV pulse waveforms. To address this need, we have developed a novel imaging modality, Specular Reflection Vascular Imaging (SRVI). The technology uses specular reflection for visualization of skin displacements caused by pressure pulsations in blood vessels. SRVI has been tested on 10 healthy volunteers. 10-seconds videos of the neck illuminated with a diffuse light source were captured at 250 fps. SRVI was able to identify and discriminate skin displacements caused by carotid artery and jugular vein pulsations to extract both carotid artery and jugular vein waveforms, making them easier to be visualized and interpreted. The method provided a 6-fold improvement in signal strength over a comparator remote PPG dataset. The current pilot study is a proof-of-concept demonstration of the potential of Specular Reflection Vascular Imaging for extraction of JV pulse waveforms.

## Introduction

Cardiovascular disease (CVD) is a leading cause of death worldwide and was responsible for 31% of all deaths in 2015. In the US, cardiovascular disease (CVD) was responsible for 647,457 deaths in 2017, which accounted for 23.0% of total deaths (Heron, 2019). The prevalence of CVD (comprising coronary heart disease, heart failure, stroke and hypertension) in US adults ≥20 years of age is 48% overall (121.5 million in 2016) (Benjamin et al., 2019).

Fluid pressure pulsations in blood vessels incorporate information about mechanical properties of vessels in the circulatory system. By examining the pressures developed within the major arteries or veins, clinicians can infer information about the heart’s functions.

The jugular vein is a major venous extension of the heart’s right atrium. Central venous pressure (CVP) is the clinical gold standard for understanding cardiac mechanical function (Cook, 1990). However, CVP measurement requires invasive catheterization using a central line catheter (CLC) inserted into the superior vena cava, or right atrium, via the jugular vein. CLC requires a cardiologist subspecializing in such invasive diagnostic procedures, as well as an appropriately staffed operating room, all of which can result in significant expense. Further, complications include pain at cannulation site, local hematoma or bleeding, infection (both at the site as well as bacteremia), misplacement into another vessel (possibly causing puncture or cannulation), vessel laceration, or dissection, air embolism, thrombosis, and pneumothorax possibly requiring a chest tube (Tse and Schick, 2021). Therefore, despite the clinical value of CVP, many patients are not indicated for CLC for the sole purpose of CVP measurement. CLC is reserved primarily for emergent situations and ICU settings (Cook, 1990; Mendenhall et al., 2021). Additionally, since the CLC catheter facilitates a single-point measurement, then no spatial information can be assessed, potentially leading to omission of important clinical information (Ashley and Niebauer, 2004).

Jugular Venous (JV) pressure is a proxy for CVP, which can be assessed non-invasively. Specifically, changes in fluid pressure within blood vessels cause deformations of the surrounding tissues and skin, particularly where vessels are close to the body surface. Visual observation of skin displacements in the jugular vein proximity is a clinically established technique to evaluate the JV pressure and JV pulse waveforms (Applefeld, 1990).

The JV pressure can be measured non-invasively during a physical examination (Applefeld, 1990). The patient is put in a reclined position (45–60° from the horizontal position). The pulsating venous column’s height is manually measured (in centimeters of water) above the right atrium’s midpoint by a ruler.

JV pressure can provide insight into cardiac function associated with the right heart chambers without direct observation of the heart itself, such as resistance diseases (e.g., pulmonary hypertension, tricuspid stenosis (Applefeld, 1990)), mechanical diseases (e.g., tricuspid regurgitation (McGee, 2012)), electrophysiological disorders (e.g., atrial fibrillation, heart block, atrioventricular dissociation (McGee, 2012)), pericardial disease (e.g., tamponade, pericarditis (McGee, 2012)), and heart failure.

In addition to JV pressure measurements, physicians may acquire additional information by analyzing JV pulse waveforms. The normal JV pulse contains three positive waves, labeled as “a,” “c,” and “v”, respectively. According to (Applefeld, 1990), “these positive deflections occur, respectively, before the carotid upstroke and just after the P wave of the ECG (a-wave); simultaneous with the upstroke of the carotid pulse (c-wave); and during ventricular systole until the tricuspid valve opens (v-wave)”. They can be used for calculation of JV pressure; however, their primary utility is embedded in their relative amplitudes.

According to (Stanford Medicine, 2021) jugular vein examination has a clinical significance. For example, an elevated “a” wave indicates resistance to right atrial emptying, which may occur at or beyond the tricuspid valve (pulmonary hypertension, rheumatic tricuspid stenosis, right atrial mass or thrombus). Similarly, a missing “a” wave is indicative of absence of atrial contraction, common in atrial fibrillation (Stanford Medicine, 2021).

As we mentioned, the JV pressure and JV pulse waveforms can be measured non-invasively during a physical exam (Applefeld, 1990) through physician observation of the aforementioned skin displacements. However, learning to evaluate the JV pulse is perhaps one of the most challenging to master of all physical diagnosis techniques (Applefeld, 1990). It is crucial that the physician distinguishes between venous and arterial pulsations, which can be assisted by illumination with a tangential light source (Applefeld, 1990). The jugular is differentiated through ready occlusion by application of light pressure, and being non-palpable (Applefeld, 1990). Further, the waveform is biphasic in a reclined position, exhibiting a-, c-, and v-peaks appearing in rapid succession, complexifying the understanding of any given peak (McGee, 2012). The clinician may also choose to measure a reference arterial pulsation, such as that occurring in the carotid on the opposite side of the neck (Applefeld, 1990) or a radial artery. Further information may be gleaned by applying pressure in the right upper quadrant of the abdomen and observing the mean JV pressure; under normal conditions, any rise in pressure will only be transient (Applefeld, 1990).

In recent years, several approaches have been proposed to tackle this clinical challenge. In particular, ultrasound has been proposed to measure the JV pulse waveform through Doppler velocity imaging (Sisini et al., 2015; Zamboni, 2016). However, despite the recent increasing affordability of ultrasound methods given advances in handheld ultrasound devices, this technology requires constant stable probe skin contact, trained ultrasound technicians, and is only able to provide axial hemodynamic information. Moreover, contact with the skin may obstruct the venous flow and affect results.

Recently, non-contact approaches have been proposed to measure the deformation of the JV and the carotid artery as an indication of the pressure. These methods assume that the deformation waveform shares similar features to the pulse waveform. (Amelard et al., 2017; Moço et al., 2017; HajiRassouliha et al., 2019) have used non-contact techniques to measure JV or carotid artery deformation waveforms. The JV pulse waveform extraction method of (Amelard et al., 2017) is based on photoplethysmography (PPG) imaging using a near infrared camera with an optical bandpass of 850–1000 nm, to maximize photon penetration and minimize melanin absorption. (Moço et al., 2017) proposed a method to measure skin motion cardiac-related frequency components under nonuniform light using a color camera, assuming that carotid artery wall displacements dominate the motion. An alternative approach was proposed by (HajiRassouliha et al., 2019), who used a monochrome camera to measure skin deformations for carotid artery waveform measurements using subpixel image registration directly. Later, the technique was extended to JV pulse waveform measurements (Tang et al., 2018).

The primary disadvantage of the solutions described above is that in a realistic clinical environment the results will be heavily affected by motion artefacts, ambient light conditions, and skin color tone of the patients.

The goal of the current study is to develop the proof of concept of a robust remote monitoring tool, such that the technology may be used in the future in the clinic for reproducible and quantifiable measurements of JV pulse waveforms. We present the proof of concept of a new methodology, termed Specular Reflection Vascular Imaging, SRVI, which uses specular reflection for visualization of skin displacements caused by pressure pulsations in blood vessels. Below, we briefly describe the concept of the methodology.

Light interacts with the skin or any other body surface tissue (e.g. mucosa) in several possible ways. Firstly, some portion of light penetrates into the tissue and experiences multiple acts of absorption and scattering. As a result, some light will be absorbed, and some will emerge from the tissue; such emerged light is called a diffuse reflectance. This light brings information about internal optical properties of the turbid tissue.

However, some part of light will be reflected in mirror-like fashion from the surface of the tissue due to mismatch in refraction index on the interface between the tissue and the air—a Fresnel (or specular) reflection. For the normal incidence, the coefficient of specular reflection *r*_*s*_ will depend on the relative index of refraction *n* (see, for example, (Stone & Whalen, 1982; Douplik et al., 2013)):

$$r_{s}=\frac{{\left(n-1\right)}^{2}}{{\left(n+1\right)}^{2}}$$ (1)

The specular reflection has very small dependence on the wavelength of the incident light. For realistic tissues (*n* = 1.33–1.45), typical values of r_s_ are quite low, in the range of 2–4% percent (Liu et al., 2011; Douplik et al., 2013). In most imaging applications even such a low value of specular reflection coefficient can pose a significant problem of hot spots and mask the useful signal. While diffuse reflection from the skin in the visible range of spectrum is typically in the range of 15–60% (Douplik et al., 2013; Descalle et al., 1998), it is distributed across the large (2π) solid angle. Thus, its angular density is much lower than the angular density of the specular reflection, which is concentrated in a very narrow solid angle.

**FIGURE 1:**
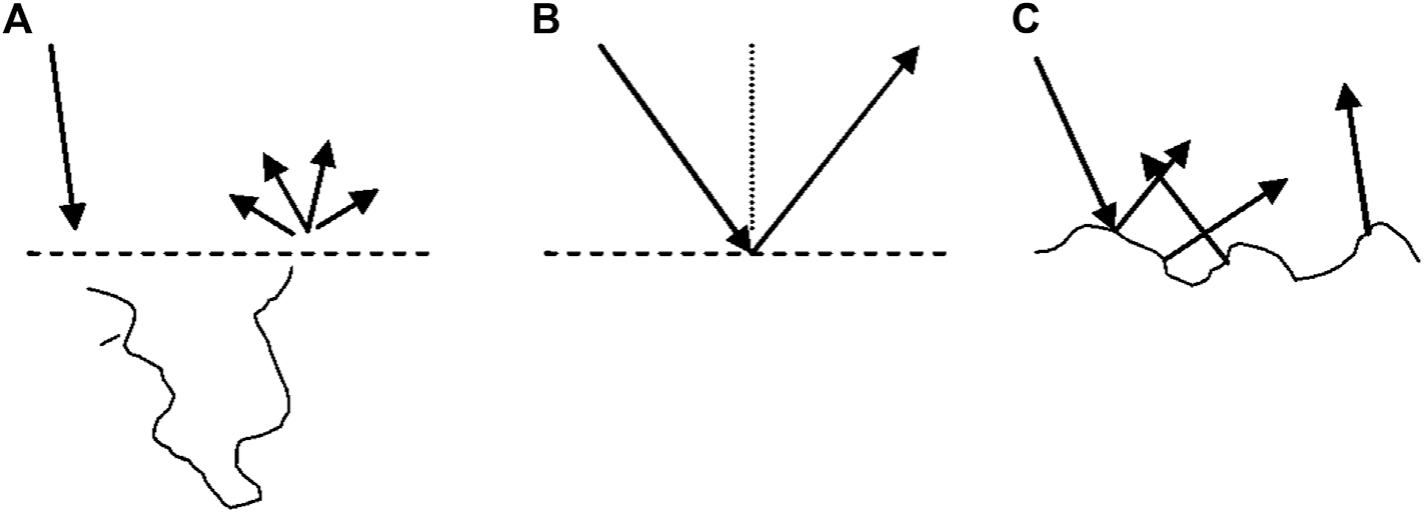
Three types of reflection from the turbid tissues. Diffuse reflection **(A)** and specular (Fresnel) reflection from smooth surface **(B)** and rough surface **(C)**.

However, specular reflection from the skin is not always noticeable. This phenomenon is attributed to the roughness of the surface (Guzelsu et al., 2003). If the surface is not smooth, then the various parts of the surface are oriented at different angles and thus scatter lights in different directions. This type of scattering is commonly observed on matte surface finishing of metal or plastic and oftentimes referred to as “diffuse reflection” (Zook, 1976). However, to avoid confusion, we will reserve “diffuse” scattering and reflection terms to describe the light, which underwent multiple scattering events within the tissue. These three mechanisms are depicted in Figure 1.

Thus, depending on the surface of the tissue, we have diffuse reflection in combination with mirror-like specular reflection from smooth surfaces or random specular reflection from rough surfaces.

While mirror-like specular reflection is typically an undesirable artifact, it can also bring useful information about the surface of the tissue. For example, it can provide information about changes in the surface geometry caused by changes of the blood volume in the subsurface blood vessels, and in particular, a carotid artery and jugular vein.

However, as mentioned before, the type of specular reflection (smooth vs. rough surface) depends on the peculiarity of the skin. For example, in case of edematous skin, the reflection is predominantly directed (smooth surface), while aging skin loses its elasticity and the reflection will be predominantly multidirectional (rough surface).

The topical application of a skin care agent such as oil smooths the skin and increases directional specular reflection.

## Methods

### Video Data Collection

**FIGURE 2:**
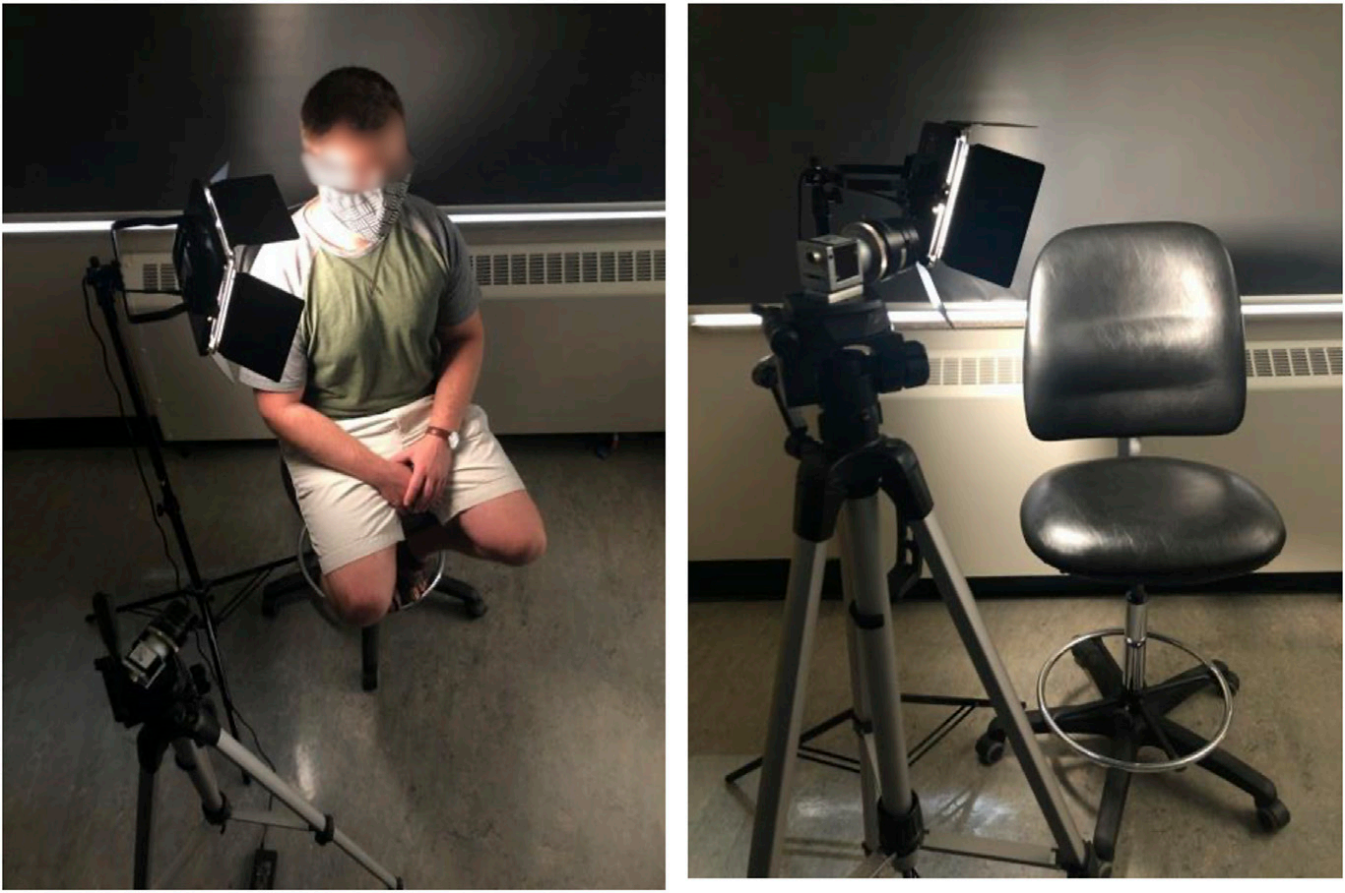
Acquisition setup.

Subjects were in an upright seated position, with a smoothening agent (baby oil (Johnson and Johnson, New Jersey, United States)) applied to the neck and illuminated with a diffuse light source (Neewer LED Video Light, Shenzhen, China). 512 × 512 pixel video data framed on the neck was captured at 250 frames per second for 10 seconds using a RGB camera (Basler acA 2000-165uc; Ahrensburg, Germany). The imaging setup can be seen in Figure 2. The target area included adjacent anatomical landmarks like the earlobe and jaw, for reference.

The study was approved by the Ryerson REB, and pilot data was captured from 10 healthy subjects, and 1 subject with ECG to validate the nature of the arterial and venous waveforms. Subjects had a variety of skin tones. Replicate videos under slightly different framings were captured.

### ECG Signal Collection

SRVI was validated by simultaneously acquiring a single lead ECG at 400 Hz (Maxim MAX86150EVSYS; San Jose, CA, California, United States). Synchronization of video and ECG was achieved through simultaneous initiation and termination of video and ECG acquisition using programmatically automated mouse clicks (Auto Mouse Click, murgee.com). Lag in ECG acquisition initiation was adjusted for by using a common termination time point.

### Video Data Processing

**FIGURE 3:**
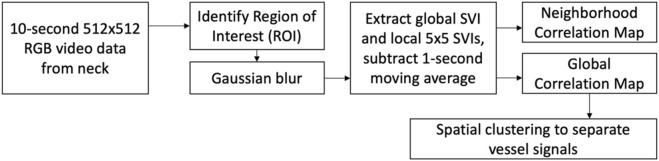
Image processing flow.

The video data was processed using MATLAB R2020b (Mathworks; Natick, MA). The complete image processing workflow is shown in Figure 3. The processing starts with the manual identification of the region of interest (ROI), which is a rectangular region of pixels excluding reference anatomical structures. A Gaussian blur was then applied to the ROI to reduce pixel noise (MATLAB “imgaussfilt”, filtering in the spatial domain with kernel size of 12 pixels and standard deviation of 51) (Gedraite & Hadad, 2011). Local SRVIs were extracted from each 5 × 5 pixel region by averaging the intensity of the pixels within the region within a given color channel (R, G or B), and repeating for all the frames to create a 250 Hz time series, which matches the camera frame rate (MATLAB matrix averaging using “mean”). The intensity averaging approach used to extract SRVI is the same as that used for remote PPG (Boccignone et al., 2020). Low frequency information not relevant to the present work was excluded from the SRVI signal by subtracting the 1-second moving average (MATLAB “movmean” with local 250-point means).

**FIGURE 4:**
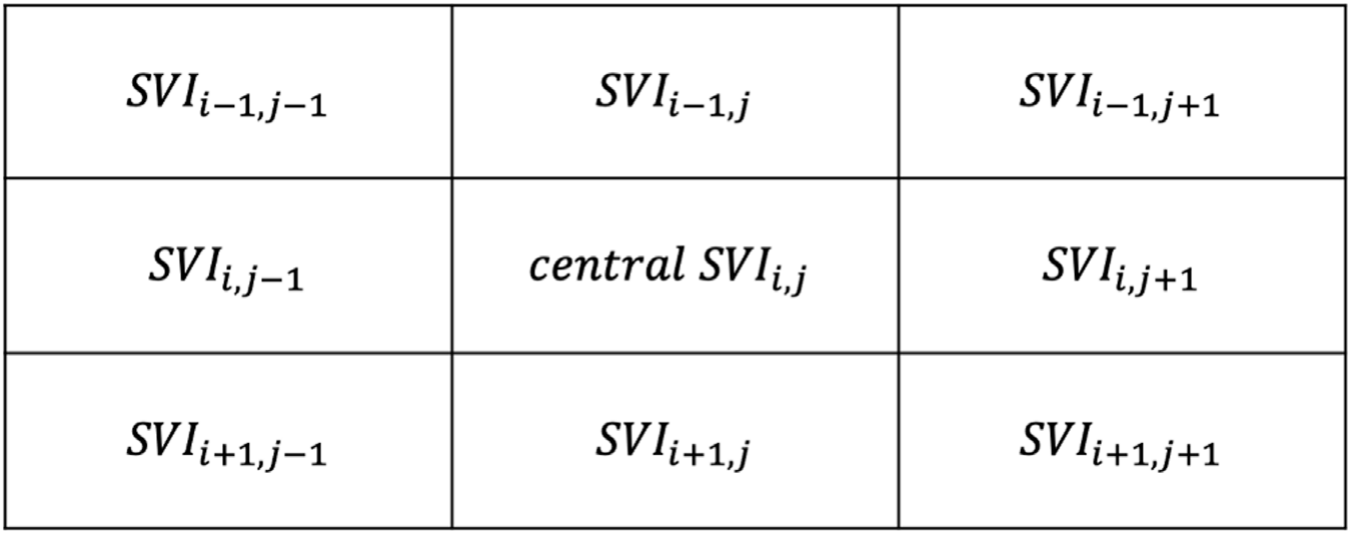
Neighbors to the central region at position *i*,*j*, with *i* representing rows and *j* representing columns.

An exploratory neighborhood correlation analysis examined the extent to which each SRVI signal is related to its neighbors using Pearson correlation. Namely, we have explored the relationship between each local SRVI (at grid position i, j) and it is eight neighbors, i.e., the regions that are directly above (row i−1), below (row i+1), left (column j−1) and right (column j+1) of the central SRVI, as well as the four diagonals (combinations of i−1, i+1, j−1, j+1), as shown in Figure 4. The Pearson correlation coefficient was evaluated between in each of the eight cases (MATLAB “corr”), averaged to represent the relationship of the central SRVI signal to its neighborhood, and represented as a neighborhood correlation map (MATLAB “imagesc”, with alphadata 0.7, pbaspect configured to match the incoming image dimensions, and colormap jet). Pearson correlation was chosen as the similarity metric since it can capture a variety of changes between pairs of SRVI signals; for example, morphology and timing. (Liu et al., 2016) performed a similar analysis with local rPPG signals extracted from the face, with the goal of detecting whether the face being presented to a facial recognition system is genuine, or a realistic mask that may otherwise fool the system. The authors extracted local rPPG signals across the face, and leveraged the knowledge that rPPG signals from adjacent skin must have high similarity to confirm that the face is genuine. In this case, the maximum value of the cross-correlation spectrum was used as the similarity metric, whereas we have used Pearson correlation to understand the relationship between spatially proximal SRVI signals.

**FIGURE 5:**
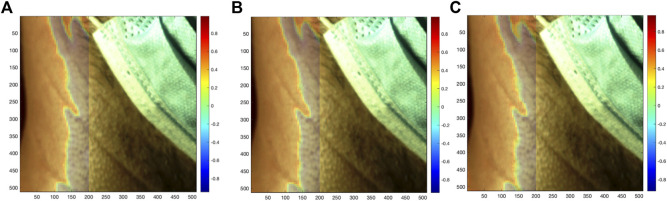
Correlation of each local SRVI (red **(A)**, green **(B)**, and blue **(C)** channels, respectively) to the global SRVI extracted from the ROI (value of Pearson correlation coefficient shown using coloring), overlaid as a transparent map on the first video frame.

Based on the results of the neighborhood analysis, a common reference against which each local SRVI could be compared was anticipated to be useful. The common reference was set to the global SRVI from the full ROI, which was extracted in the same manner as local SRVI except all pixels were included rather than 5 × 5 regions of pixels. The relationship between each local SRVI and the global SRVI was quantified using Pearson correlation (as previously described in the neighborhood analysis). A global correlation map (such as those shown in Figure 5) was generated by first displaying the first frame in the video for anatomical context, and then overlaying the grid of each local SRVI’s correlation to the global SRVI (MATLAB “histeq” to enhance contrast of the first frame using histogram equalization, then “imagesc” with alphadata 0.2, pbaspect configured to match the incoming pixel dimensions and colormap jet).

**FIGURE 6:**
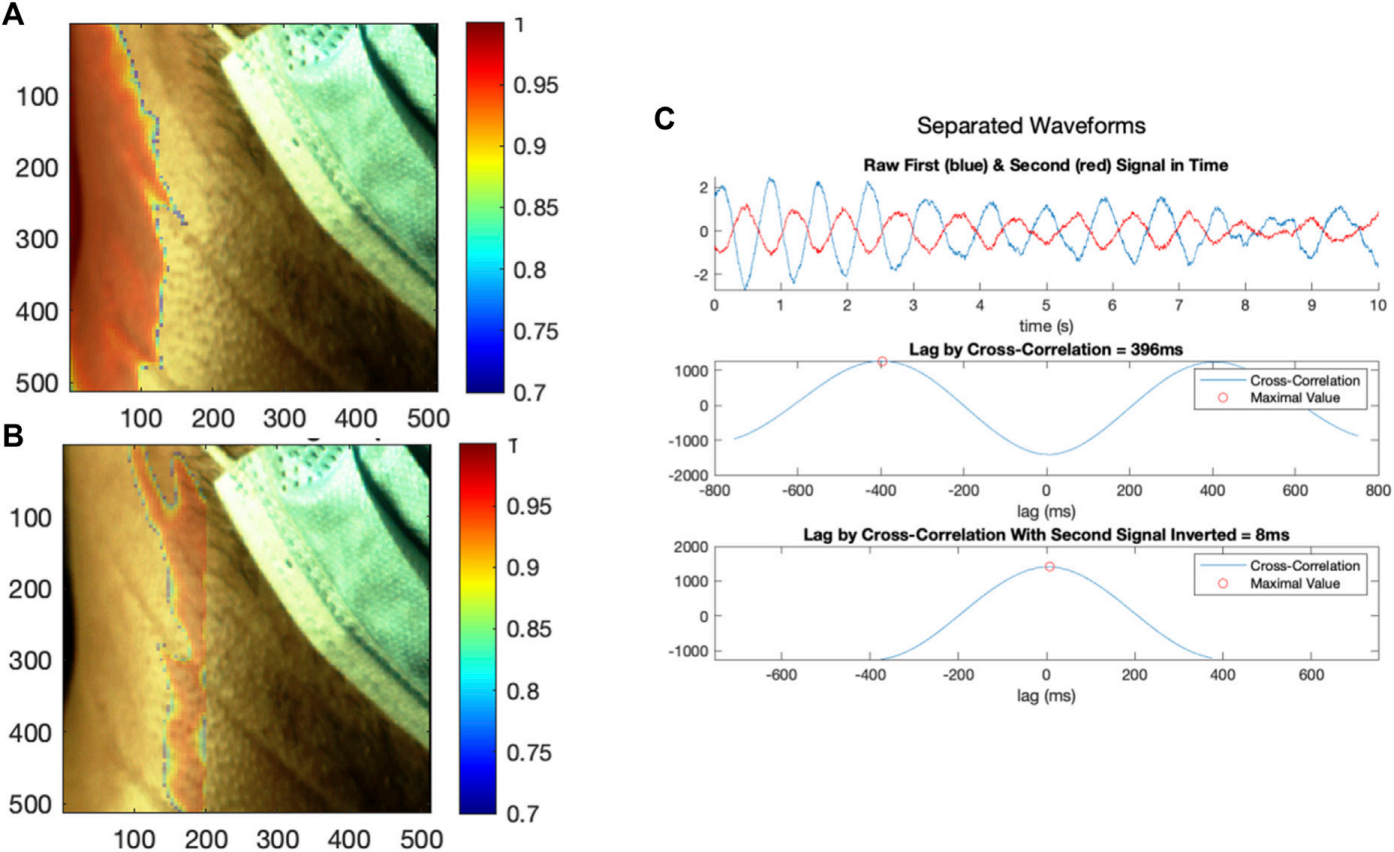
**(A)** The first region generating local SRVI signals with high internal correlation, with the colour bar indicating correlation of the local SRVIs to the average SRVI in that region, **(B)** the second region generating local SRVI signals with high internal correlation, with the colour bar indicating correlation of the local SRVIs to the average SRVI in that region, and **(C)** the SRVI signals from the first and second regions plotted in the time domain, and analyzed using cross-correlation (as acquired, and with the second signal inverted).

Spatial clustering was then performed on the global correlation map to identify disparate regions characterized by highly negative correlations and highly positive correlations. Spatial clustering has been performed in a similar application; specifically, (Luijtelaar et al., 2014), performed a density-based clustering of signals represented in a feature space to segregate rPPG signals originating from tissue from noise signals originating from non-tissue regions in the video frame. The authors then performed a cluster-growing operation, such that signals originally classified as not being generated by skin may be re-added using information from the initial clustering. In our methodology, an initial threshold for highly negative correlations was established at the bottom decile of correlations, and for highly positive correlations, at the top decile. Local SRVI signals with correlations above the top decile were averaged together to create an initial candidate signal for the highly correlated region, and local SRVI signals with correlations below the bottom decile averaged for the negatively correlated region. The correlation of each local SRVI was then calculated to the candidate high-correlation signal, and to the candidate low-correlation signal, creating two new correlation maps. A threshold was established on each map to only preserve local SRVIs with correlation greater than 0.70, yielding a binary map for both the high-correlation and low-correlation signals. These binary maps were analyzed as images to detect contiguous regions (MATLAB “regionprops” configured to extract the area and convex hull). The largest contiguous region in each map was chosen based on the detected region with the maximal area, and each local SRVI was assessed for inclusion in that region by testing whether it was captured by the convex hull (MATLAB “inpolygon”). The local SRVIs included in the largest high-correlation region were averaged together to generate the final high-correlation SRVI, and local SRVIs in the largest low-correlation region were averaged to generate the final low-correlation SRVI. As in Figure 6a, the high-correlation signal region was shown overlaid on the first frame for anatomical reference, with all other segments of the map hidden (MATLAB “histeq” to enhance contrast of the first frame using histogram equalization, then “imagesc” with alphadata 0.4 for high correlation regions and 0 for others, pbaspect configured to matched the incoming pixel dimensions and colormap jet). As in Figure 6b, the low-correlation signal can also be plotted in an equivalent manner.

The local SRVI signals composing these disparate regions were averaged to create representative signals. The representative signals which were compared to each other using cross-correlation, as well as to the simultaneous ECG (when available). SRVI signals were also compared to those made available from Amelard et al. (Amelard et al., 2017).

### Combined SRVI and ECG Data Processing

The 1-second centered moving average (Matlab movmean) was removed from both ECG and SRVI, to exclude any baseline wander. The ECG was smoothed using a 40 ms moving average, and the SRVI data with a 20 ms moving average. The SRVI was scaled to have similar amplitude as the ECG, for visualization purposes.

### SRVI and PPG Signal Strength Calculation

To calculate the signal strength of the dynamic cardiac pulsation component in SRVI and PPG signals, an envelope approach using moving maximum and minimum was invoked. Specifically, the moving maximum and minimum of *k*-points was calculated for the signal, then the point-wise difference between the maximum and minimum time series was evaluated, and the median value was taken as the representative amplitude to exclude any outlying data. *k* was calculated with respect to the minimum realistic heart rate of a healthy subject at rest, which presumed to be 50 beats per minute (0.83 Hz), and the number of frames per second captured by the acquiring camera according to the following equation. *k* = 301 for SRVI and *k* = 72 for PPG.

$$k=\;\frac{1}{0.83Hz}*fps$$ (2)

## Results

**FIGURE 7:**
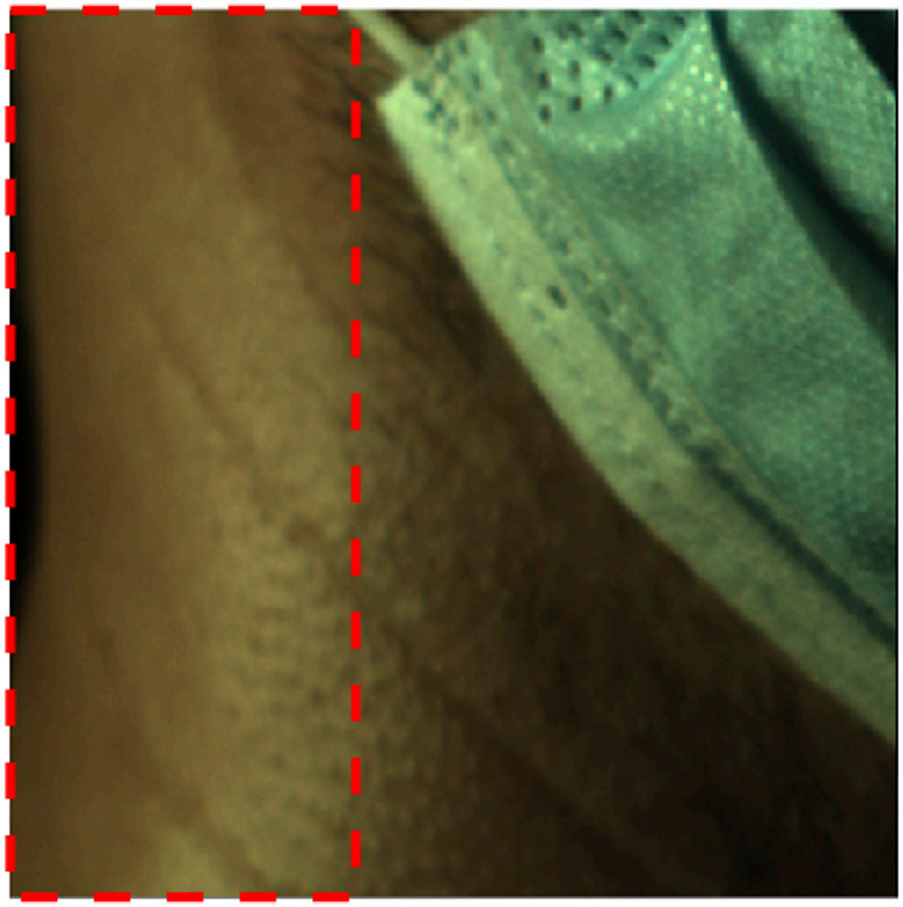
Framing and region of interest (red rectangle) that excludes the mask.

The camera framing on an exemplar subject is shown in Figure 7, including a red box to delineate the region of interest (ROI). The complete frame captures anatomical structures and other reference points (i.e., chin, mask, etc.), while the ROI captures the maximal rectangular area expected to contain the carotid and jugular signals. While an exemplar subject is presented here, the analysis results for all subjects and signals can be found in Supplementary Materials Section 2.

### Local Correlation Analysis

Local SRVI signals were extracted using the red channel, which will be used for all analyses, for consistency. SRVI results were similar for all RGB channels, as would be expected given that each channel captures specular reflection in a similar manner.

**FIGURE 8:**
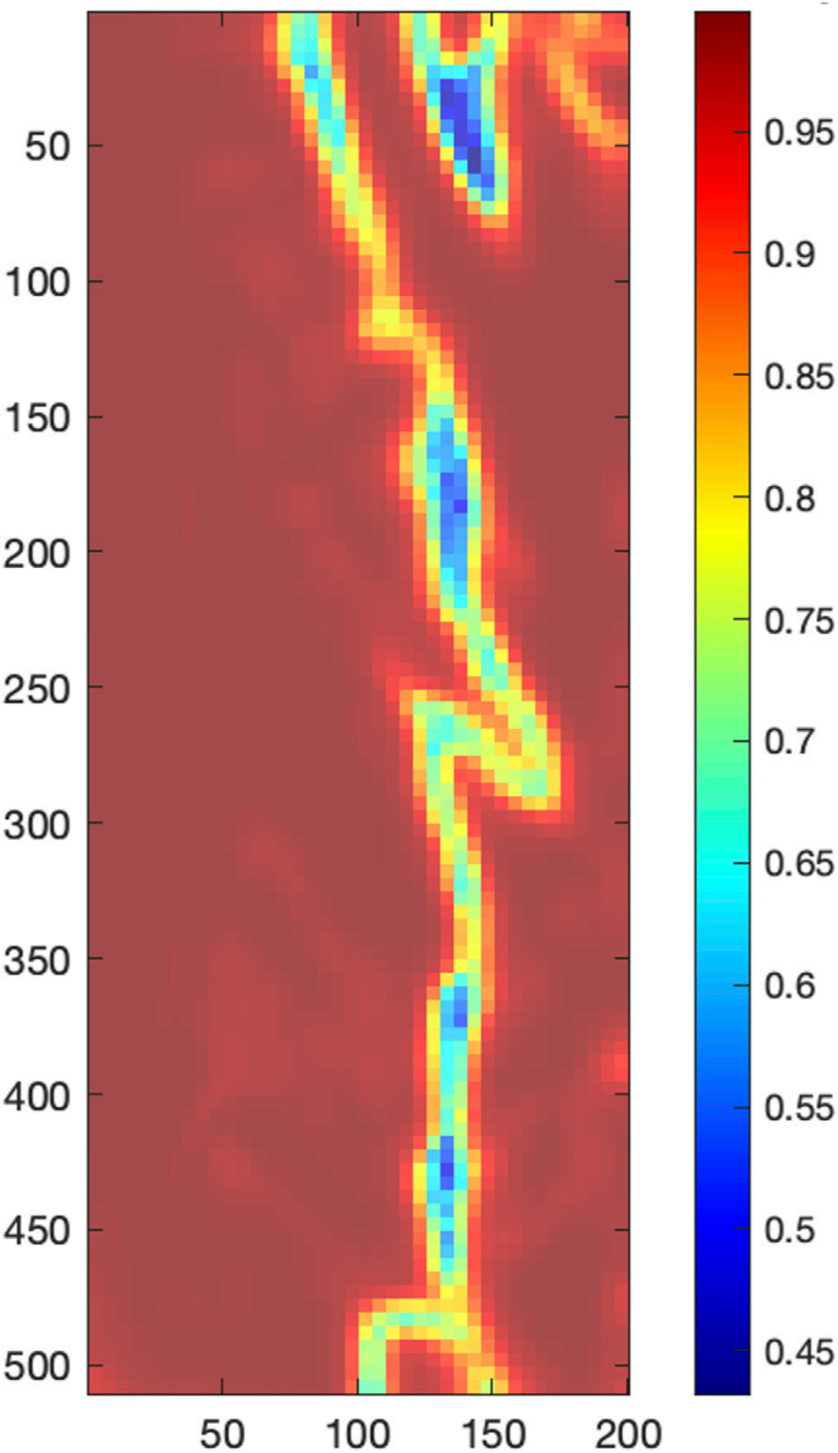
Local correlation analysis, with the color bar indicating the average Pearson correlation coefficient of each central local SRVI to its 8 neighbors.

First, the neighborhood correlation analysis was performed, the result of which is shown in Figure 8. The neighborhood correlation analysis revealed that most local SRVI signals were highly correlated to their neighbors (>0.95), except for a band running vertically in the ROI, where the correlation dropped significantly (approximately 0.45–0.85) (Figure 8).

The neighborhood correlation result was further explored by extracting the global SRVI signal from the ROI (Figure 7) and calculating the correlation to each local SRVI signal to create a global correlation map. Figure 5 is a visualization of that result, where the correlation map for each channel (red, green, and blue, respectively) is shown as a transparent overlay on the first video frame (not reduced to the ROI to include reference anatomical structures). Through comparison of both sets of results, it became clear that the vertical band identified in the neighborhood correlation marked the division between two disparate regions in the plot, as shown in the global correlation map. Each color channel produces virtually identical results; therefore, as previously mentioned, the red channel was used for all further processing for conciseness.

### Vessel Signal Extraction Using Spatial Clustering

Spatial clustering was used to separate these distinct regions, and SRVI signals were computed for each using the average of their local SRVIs. Figure 6 shows the separation of the regions (a, b), as well as the relationship between the two resultant SRVIs (c). The signals are now presumed to be putative vessel signals, with one representing arterial flow in the carotid, and the other representing venous flow in the jugular. However, from Figure 6 it is not yet clear which signal represents the carotid artery waveform and which reflects JV pulse waveform. During each signal, 12 cycles occur in a 10-seconds period, resulting in a rate of 72 beats per minute (a realistic heart rate). The lag between the signals is 396 ms (which is realistic–see Table 1).

**TABLE 1:** Comparison of lag and inverted lag between SRVI and (Amelard et al., 2017) data.

Data	Lag (mean ± SD)	Inverted lag (mean ± SD)
SRVI Data	390 ± 38 ms	32 ± 21 ms
Amelard et al. (2017)	427 ± 99 ms	47 ± 84 ms

The process was performed for 20 videos from 10 subjects, with 1 analysis failure, resulting in 19 successful analyses, and can be found in the Supplementary Appendix. The lag between the pair of signals within each video was calculated using cross-correlation, which resulted in a lag across the dataset of 390 ± 38 ms. The signals seem to be anticorrelated, so we explored the possibility that it is the same signal, but just inverted. In the case of a single video, as shown in Figure 6C, the inversion results in a lag of 8 ms. Further, across the entire dataset, the lag was 32 ± 21 ms, indicating that the signals are not simply the inverse of each other. Finally, the amplitude of the cardiac pulses was extracted from the dataset, revealing an average signal strength (trough to peak, excluding outliers) of 1.98 pixel-intensity. To provide context, a dataset of video PPG collected with an iPhone (Burton et al., 2021) was analyzed for signal strength in the same methodology in each color channel (since PPG can differ, unlike SRVI), yielding average signal strengths of 0.29, 0.37, and 0.31 pixel-intensities for red, green and blue, respectively. In comparison to that PPG data, SRVI has a 6.83-fold improvement in signal strength in the red channel, 5.35-fold in blue and 6.39-fold in red.

### Validation Using Comparison With Literature

We also performed a comparison with (Amelard et al., 2017). The Amelard et al. dataset was published as a Supplementary Material, and the lag and inverted lag from this data was analyzed to provide a comparison to our results using the SRVI approach. Specifically, Amelard et al. collected data from 24 subjects, and for each, identified 5 anatomical regions that were highly correlated to the arterial waveform as captured using a finger clip PPG (therefore identified by Amelard et al. as carotid signals), and 5 regions that were highly negatively correlated to the arterial waveform (therefore identified by Amelard et al. as jugular signals). The signals were extracted from each region and made publicly available, though it is not possible to determine from the published data which pair of positive and negative signals were most anatomically proximal to each other. Therefore, the lag and inverted lag were calculated for each possible pairing of signals (25) from each subject (24), for a total of 600 observations. The mean and standard deviation for both the SRVI and Amelard et al. data are shown in Table 1. The raw data underlying the SRVI summary statistics can be found in Supplementary Table S1. Statistical testing cannot be performed to compare the SRVI and Amelard et al. data due to the necessity to create each possible signal pairing for each Amelard et al. subject, which would have the effect of overpowering the test. However, the Amelard et al. mean lag and mean inverted lag both fall within one standard deviation of the SRVI results, demonstrating that both methods result in waveforms with similar properties.

### Validation Using Synchronization With ECG

**FIGURE 9:**
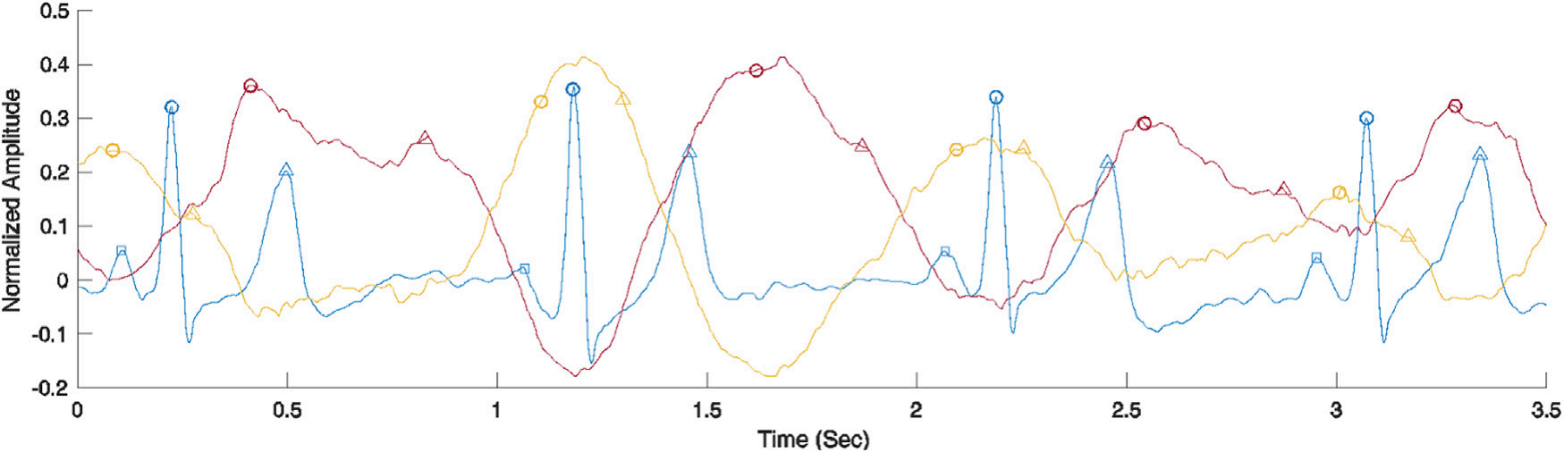
Comparison of ECG (blue) and SRVI signals (carotid artery signal in red and JV pulse signal in yellow). ECG landmarks are P-waves representing maximal atrial depolarization (squares), R-peaks representing maximal ventricular depolarization (circles) and T-wave representing maximal ventricular repolarization (triangles). The carotid artery SRVI landmarks are systolic peaks (circles) and estimated diastolic peaks (triangles). The JV pulse SRVI landmarks are a-waves representing atrial contraction (circles) and c-waves representing right ventricular contraction (triangles).

To elucidate the origin of each signal, we performed SRVI data collection synchronized with ECG acquired at 400 Hz. Figure 9 shows a comparison between SRVI signals and ECG over a duration of four complete cardiac cycles. The SRVI signal shown in red is maximal at or shortly after the maximal ventricular repolarization (i.e., T-wave), and delayed from the maximal ventricular depolarization (i.e., R-peak) by a lag corresponding to a reasonable median pulse transit time (PTT) of 272 ms, indicating that this is the arterial signal originating in the carotid.

There is a dearth of data on heart-to-carotid PPT in healthy subjects, but typical heart to periphery (e.g., fingertip) PPT is about 250 ms, as measured with an ECG and a finger clip PPG (Pitson et al., 1994). The observed median PPT in the SRVI data shown in Figure 9 of 272 ms is close to that value. The observed peaks in the SRVI jugular signal are maximal in the region of the R-peaks, which aligns with previously described properties of jugular signals (García-López & Rodriguez-Villegas, 2020). Further, as expected, the peaks of the SRVI jugular and SRVI carotid signals are flattened by various physiological events occurring through the course of the cardiac cycle. As shown in Figure 9, in the case of the SRVI jugular peak, the flattening is caused by an initial “a” wave after the atrial depolarization (i.e., p-wave), followed a second “c” wave after right ventricular depolarization. Also as shown in Figure 9, in the case of the SRVI carotid peak, there are distinct landmarks corresponding to both cardiac systole and diastole. The characteristics of these SRVI signals and their relationship to ECG align with published properties of jugular and arterial signals, with the exception of the absence of a “v” wave, which typically presents as a relatively small amplitude wave in the after the offset of the ventricular repolarization and prior to atrial depolarization (i.e., in the TP segment) (García-López & Rodriguez-Villegas, 2020). The anatomical position of the subjects (seated) may contribute to this deviation from the expected JV pulse waveform, as observed by other investigators collecting this waveform in a seated position (Moço et al., 2018a; Proto et al., 2021).

## Discussion

Here, we presented a pilot study of a novel imaging approach, SRVI, based on capturing skin displacements caused by blood pressure waveforms in major neck vessels using specular reflection. As a proof of concept, the SRVI technique was able to distinguish between two areas on the neck, which, based on ECG comparison, can be attributed to carotid artery and jugular vein. Differentiation of these regions enables the extraction of carotid artery and jugular vein waveforms.

SRVI exhibited similar waveform properties to those from a PPG-based method described in the past by Amelard et al. (Amelard et al., 2017). Further, SRVI is aligned with the ECG in a physiologically expected manner. Both these observations support the validity of the SRVI waveforms within the constraints of our dataset. Further, as expected, the method shows consistency through all color channels of the color CMOS camera.

### Advantages of SRVI

SRVI has several substantial advantages over other optical methods, the most common of which is PPG (where the most direct comparison is to remote PPG). Firstly, SRVI is insensitive to the skin tone, since the specular reflection coefficient depends on the index of refraction only. Lack of dependence on skin tone is a critical property, given recent reporting that has identified potential skin tone biases of PPG. For instance, (Sjoding et al., 2020), investigated the occurrence of occult hypoxemia across patients who self-identified as White or Black, which is a true oxygen saturation of <88% given a PPG-quantified saturation from 92 to 96% (i.e., a false negative from PPG on detection of low saturation). (Sjoding et al., 2020) reported that occult hypoxemia occurred in 88/749 (12%) of patients who self-identified as Black, as compared to 99/2,778 (4%) of patients who self-identified as White. In contrast to these known racial biases of PPG, SRVI should be universally applicable to all skin tones, as supported by the pilot results, which contained a variety of skin tones (see Supplementary Appendix).

Secondly, since SRVI is based on specular reflection from the surface of the skin, it has the potential to generate stronger signals as compared to PPG-based methods, which was observed in the present SRVI data in comparison to video PPG data (Burton et al., 2021), which showed that SRVI has 6.83, 5.35, and 6.39-fold improvements in signal strength over PPG. This finding is not unexpected, due to the mechanism of PPG sensing. PPG sensors can either be transmission mode or reflection mode, with the remote PPG being an extension of the latter. In this case, light scattering from the tissue is detected by a contactless sensor. The amplitude of such signals is known to be low, with (Moço et al, 2018b) providing a simulated-based explanation for the mechanism. (Moço et al, 2018b) simulated photon propagation in a multi-layered turbid media, configured to represent the optical properties of six layers of skin in the palm or finger pad, and found that depth origin of the PPG signal was from approximately 1.5–2 mm (and consist with variation in blood volume in the tissue). The depth that the photons must travel to exit the skin and strike the sensor may account for the known low amplitude of such reflectance-based PPG signals, while SRVI signals detect displacements on the surface of the skin.

### Limitations of SRVI

It is possible that the specular reflectance from the surface of the skin could contain a PPG component, which can be particular true in wavelengths with significant absorption by oxy- and deoxyhemoglobins (blue and green ranges of the spectrum). Though we found that all colour channels yielded similar SRVI signals, the red channel of the camera was used herein to minimize any contribution from PPG. Our current work is limited by the number of subjects (*N* = 10), as well as all subjects being healthy volunteers. Lack of relevant cardiac disease limits our ability to project the performance of SRVI on symptomatic subjects, and such data is required to assess the validity of the waveforms within that population. Collection in a reclined position will also be of value, to better compare to other published data, and because in seated subjects (such as we evaluated), the JV drains and clinically becomes less obvious. Therefore, future work will include collecting a larger dataset to assess reliability, as well as observing waveforms of the jugular vein pulse in patients with elevated jugular vein pressure in a reclined position, and recording ECG in all subjects to confirm timing. Further, the development of SRVI has been focused on the waveform itself, and it currently not capable of producing assessments of the jugular venous pressure. We believe that it may be possible to extend the methodology to estimate jugular venous pressure, which will be explored in future work. Finally, while the SRVI data acquisition is relatively low-cost in the context of medical devices, it is not insignificant. The analysis steps described herein could be automated to minimize the educational burden to administer the data capture, with the caveat that the computation requires a sufficiently resourced device to execute.

## Data Availability

The raw data supporting the conclusions of this article will be made available by the authors, without undue reservation.

## References

[B1] Amelard R. Hughson R. L. Greaves D. K. Pfisterer K. J. Leung J. Clausi D. A. (2017). Non-contact Hemodynamic Imaging Reveals the Jugular Venous Pulse Waveform. Sci. Rep. 7 (1), 1–10. 10.1038/srep40150 28065933 PMC5220303

[B2] Applefeld M. M. (1990). “The Jugular Venous Pressure and Pulse Contour,” in Clinical Methods: The History, Physical, and Laboratory Examinations. 1. Editors Walker H. K. Hall W. D. Hurst J. W. (Boston, MA: Butterworths). Chapter 19. 21250143

[B3] Ashley E. A. Niebauer J. (2004). Cardiology Explained. London, England: Remedica. 20821845

[B4] Benjamin E. J. Muntner P. Alonso A. Bittencourt M. S. Callaway C. W. Carson A. P. (2019). Heart Disease and Stroke Statistics-2019 Update: A Report from the American Heart Association. Circulation 139 (10), e56–e528. 10.1161/CIR.0000000000000659 30700139

[B5] Boccignone G. Conte D. Cuculo V. D'Amelio A. Grossi G. Lanzarotti R. (2020). An Open Framework for Remote-PPG Methods and Their Assessment. IEEE Access 8, 216083–216103. 10.1109/access.2020.3040936

[B6] Burton T. Saiko G. Douplik A. (2021). Feasibility Study of Remote Contactless Perfusion Imaging with Consumer-Grade Mobile Camera. Manuscript Submitted Adv. Exp. Med. Biol. 1, 1. 10.1007/978-3-031-14190-4_47 36527651

[B7] Cook D. J. (1990). Clinical Assessment of Central Venous Pressure in the Critically Ill. Am. J. Med. Sci. 299, 175–178. 10.1097/00000441-199003000-00006 2316561

[B8] Descalle M. Jacques S. Prahl S. Laing T. Martin W. (1998). "Measurements of Ligament and Cartilage Optical Properties at 351 Nm, 365 Nm, and in the Visible Range (440 to 800 Nm)," in Proc. SPIE 3195, Laser-Tissue Interaction, Tissue Optics, and Laser Welding III. (International Society for Optics and Photonics).

[B9] Douplik A. Saiko G. Schelkanova I. Tuchin V. V. (2013). “The Response of Tissue to Laser Light,” in Lasers for Medical Applications. Diagnostics, Therapy and Surgery. Editor Sawston H. J. (United Kingston: Woodhead Publishing), 47–109. 10.1533/9780857097545.1.47

[B10] García-López I. Rodriguez-Villegas E. (2020). Extracting the Jugular Venous Pulse from Anterior Neck Contact Photoplethysmography. Sci. Rep. 10 (1), 1–12. 10.1038/s41598-020-60317-7 32103056 PMC7044195

[B11] Gedraite E. Hadad M. (2011). “Investigation on the Effect of a Gaussian Blur in Image Filtering and Segmentation,” in Proceedings ELMAR-2011 (IEEE).

[B12] Guzelsu N. Federici J. F. Lim H. C. Chauhdry H. R. Ritter A. B. Findley T. (2003). Measurement of Skin Stretch via Light Reflection. J. Biomed. Opt. 8 (1), 80–86. 10.1117/1.1527936 12542383

[B13] HajiRassouliha A. Tang E. J. L. P. Nash M. P. Taberner A. J. Nielsen P. M. F. Cakmak Y. O. (2019). “Quantifying Carotid Pulse Waveforms Using Subpixel Image Registration,” in Computational Biomechanics for Medicine. Editors Nielsen P. Wittek A. Miller K. Doyle B. Joldes G. Nash M. (Springer), 83–92. 10.1007/978-3-319-75589-2_8

[B14] Heron M. (2019). Deaths: Leading Causes for 2017. Natl. Vital Stat. Rep. 68 (6), 1–77. 32501203

[B16] Liu L. Bai X. Zhang Z. Zhang B. (2011). “Theoretical Calculation and Numerical Simulation of Spherical Lung Cancer Cells' Refractive index,” in Proceedings of 2011 6th International Forum on Strategic Technology (IEEE). 10.1109/ifost.2011.6021201

[B17] Liu S. Yuen P. C. Zhang S. Zhao G. (2016). “3D Mask Face Anti-spoofing with Remote Photoplethysmography,” in European Conference on Computer Vision (Cham: Springer), 85–100. 10.1007/978-3-319-46478-7_6

[B18] Luijtelaar R. Wang W. Stuijk S. Haan G. (2014). “Automatic Roi Detection for Camera-Based Pulse-Rate Measurement,” in Asian Conference on Computer Vision (Cham: Springer), 360–374.

[B19] McGee S. (2012). “Inspection of the Neck Veins” in Evidence-Based Physical Diagnosis. Elsevier, 301.

[B20] Mendenhall B. R. Wilson C. Singh K. Dua A. O’Rourke M. C. (2021). “Internal Jugular Vein Central Venous Access,” in StatPearls. Abai B. Abu-Ghosh A. Acharya A. B. Acharya U. Adhia S. G. Aeby T. C. 1. (Treasure Island, FL: StatPearls Publishing). 28613791

[B21] Moço A. V. Hamelmann P. Stuijk S. de Haan G. (2018a). “The Importance of Posture and Skin-Site Selection on Remote Measurements of Neck Pulsations: An Ultrasonographic Study,” in 2018 40th Annual International Conference of the IEEE Engineering in Medicine and Biology Society (EMBC) (5918-5921) (IEEE). 10.1109/embc.2018.8513651 30441683

[B22] Moço A. V. Mondragon L. Z. Wang W. Stuijk S. de Haan G. (2017). Camera-based Assessment of Arterial Stiffness and Wave Reflection Parameters from Neck Micro-motion. Physiol. Meas. 38 (8), 1576–1598. 10.1088/1361-6579/aa7d43 28671872

[B23] Moço A. V. Stuijk S. de Haan G. (2018b). New Insights into the Origin of Remote PPG Signals in Visible Light and Infrared. Sci. Rep. 8, 1 1–15. 10.1038/s41598-018-26068-2 29855610 PMC5981460

[B24] Pitson D. Chhina N. Knijn S. Van Herwaaden M. Stradling J. (1994). Changes in Pulse Transit Time and Pulse Rate as Markers of Arousal from Sleep in normal Subjects. Clin. Sci. 87 (2), 269–273. 10.1042/cs0870269 7924174

[B25] Proto A. Conti D. Menegatti E. Taibi A. Gadda G. (2021). Plethysmography System to Monitor the Jugular Venous Pulse: A Feasibility Study. Diagnostics 11 (12), 2390. 10.3390/diagnostics11122390 34943625 PMC8699927

[B26] Sisini F. Tessari M. Gadda G. Di Domenico G. Taibi A. Menegatti E. (2015). An Ultrasonographic Technique to Assess the Jugular Venous Pulse: a Proof of Concept. Ultrasound Med. Biol. 41 (5), 1334–1341. 10.1016/j.ultrasmedbio.2014.12.666 25704322

[B27] Sjoding M. W. Dickson R. P. Iwashyna T. J. Gay S. E. Valley T. S. (2020). Racial Bias in Pulse Oximetry Measurement. New Engl. J. Med. 383 (25), 2477–2478. 10.1056/NEJMc202924010.1056/nejmc2029240 33326721 PMC7808260

[B28] Stanford Medicine R. W. (2021). Neck Vein Examination & Wave Forms. Available at: https://stanfordmedicine25.stanford.edu/the25/neck-exam-jugular-venous-pressure-measurement.html (Accessed October 8^th^, 2021).

[B29] Stone J. Whalen M. S. (1982). Index of Refraction Dispersion Ofn‐ Andp‐type InP between 0.95 and 2.0 eV. Appl. Phys. Lett. 4112, 1140–1142. 10.1063/1.93412

[B15] Tang E. J. L. P. HajiRassouliha A. Nash M. P. Nielsen P. M. F. Taberner A. J. Cakmak Y. O. (2018). Non-contact Quantification of Jugular Venous Pulse Waveforms from Skin Displacements. Sci. Rep. 8 (1), 1–12. 10.1038/s41598-018-35483-4 30467407 PMC6250701

[B30] Tse A. Schick M. A. (2021). “Central Line Placement,” in StatPearls. Abai B. Abu-Ghosh A. Acharya A. B. Acharya U. Adhia S. G. Aeby T. C. 1. (Treasure Island, FL: StatPearls Publishing).

[B31] Zamboni P. (20162016). Why Current Doppler Ultrasound Methodology Is Inaccurate in Assessing Cerebral Venous Return: the Alternative of the Ultrasonic Jugular Venous Pulse. Behav. Neurol. 1, 1. 10.1155/2016/7082856 PMC4783538 27006525

[B32] Zook J. (1976). A Simple Model for Diffuse Reflection. Opt. Commun. 17 (1), 77–82.

