# Supplementary material for: Feasibility of Specular Reflection Imaging for Extraction of Neck Vessel Pressure Waveforms
Source: Front Bioeng Biotechnol. 2022 Mar 21;10:830231. doi: 10.3389/fbioe.2022.830231 (PMC8979108; doi:10.3389/fbioe.2022.830231)
Supplement: Supplementary file 1 [file DataSheet1.docx]

Supplementary Material

# SVI Cross-Correlation Analysis

Supplementary Material Table 1 below contains the raw data constituting the SVI summary statistics in manuscript Table 1. The complete set of analysis results for each signal in the below table can be found in the following section.

*Supplementary Material Table 1: Lag by Cross-Correlation between Intra-subject SVI Signals*

| Subject | Recording | Lag between signals by cross-correlation (ms) | Lag between signals, with one signal inverted (ms) |
| --- | --- | --- | --- |
| 1 | A | 396 | 8 |
| 1 | B | 332 | 24 |
| 2 | A | 360 | 12 |
| 2 | B | 448 | 40 |
| 3 | A | 364 | 40 |
| 3 | B | 388 | 16 |
| 4 | A | 404 | 20 |
| 4 | B | 400 | 12 |
| 5 | A | 352 | 36 |
| 5 | B | 360 | 68 |
| 6 | A | 404 | 72 |
| 6 | B | 352 | 32 |
| 7 | A | 376 | 8 |
| 7 | B | *n/a – analysis not successful* | *n/a – analysis not successful* |
| 8 | A | 448 | 24 |
| 8 | B | 456 | 52 |
| 9 | A | 384 | 16 |
| 9 | B | 348 | 8 |
| 10 | A | 452 | 60 |
| 10 | B | 380 | 52 |

# Analysis Results for All Signals

## Subject 1, Signal A

Presented in the manuscript.

## Subject 1, Signal B

| 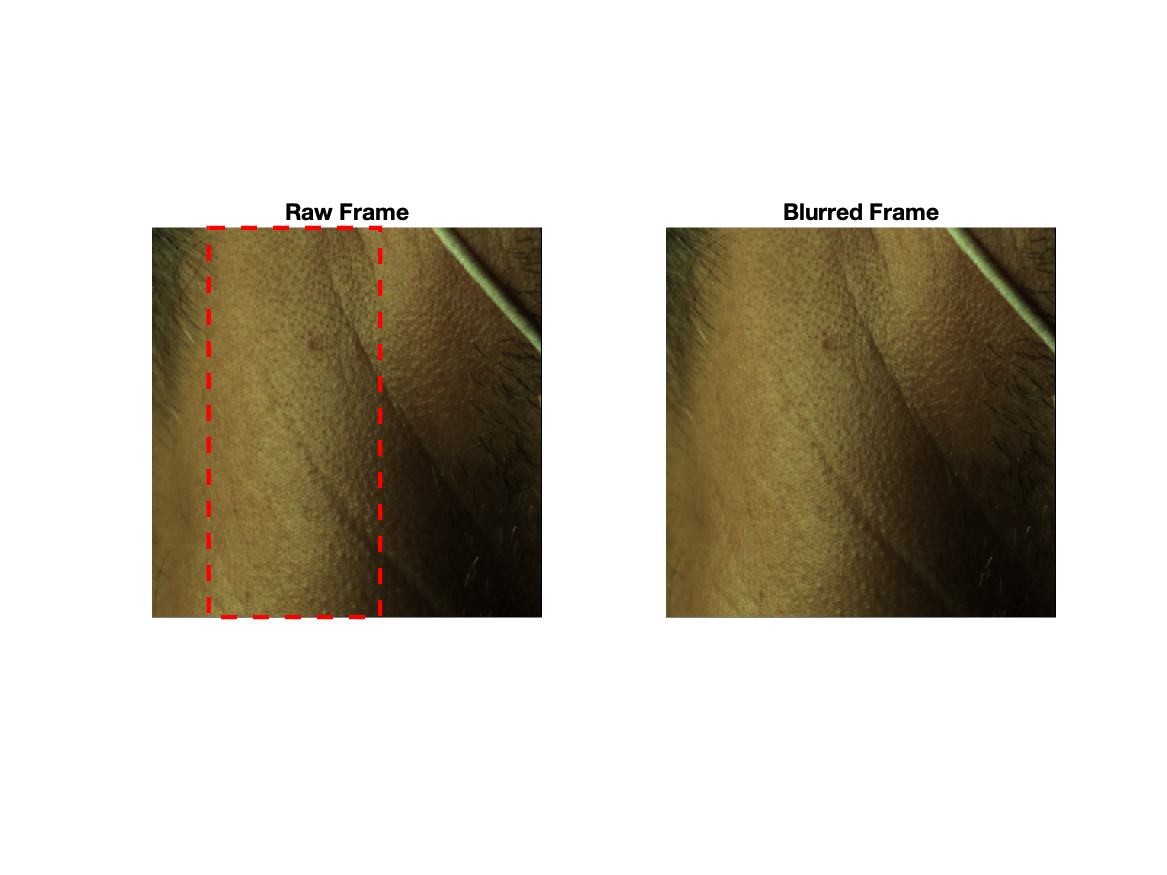 | 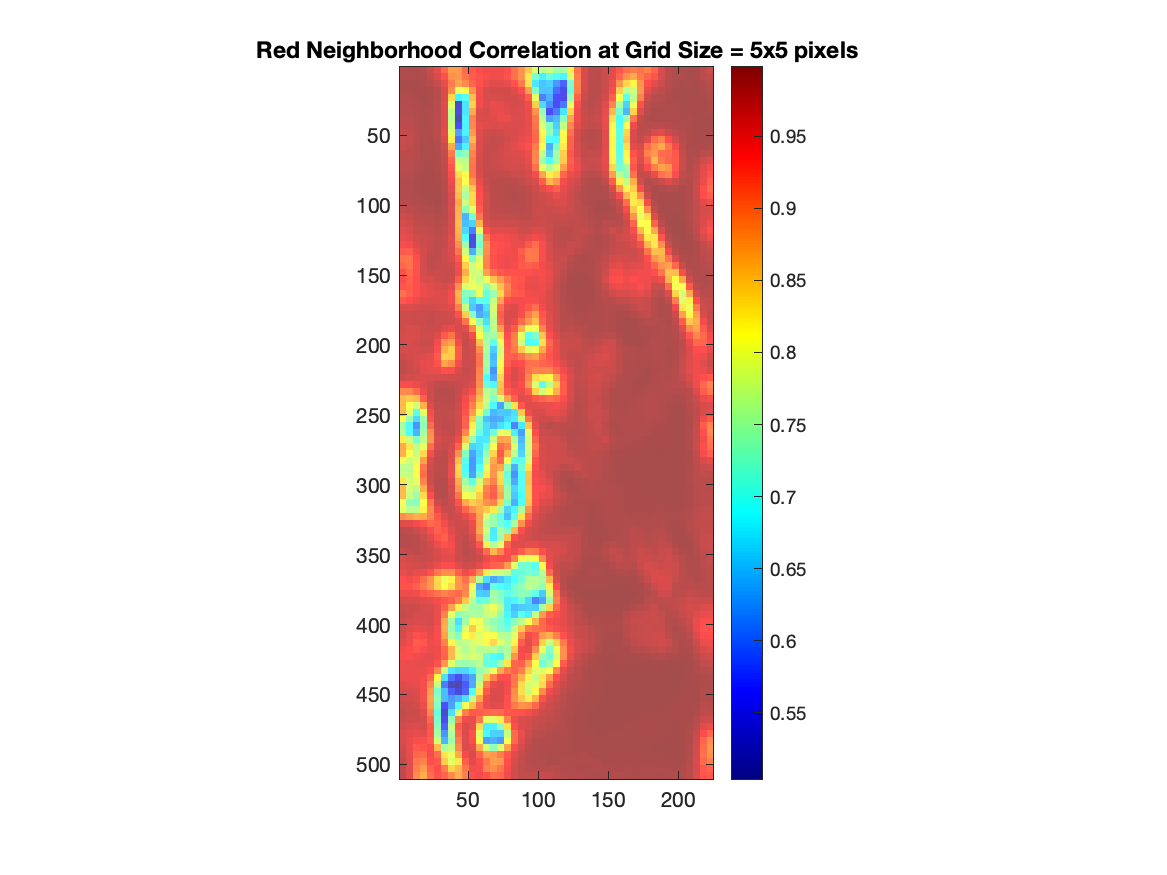 |
| --- | --- |

Figure 1: ROI (left) and local correlation analysis (right)

| 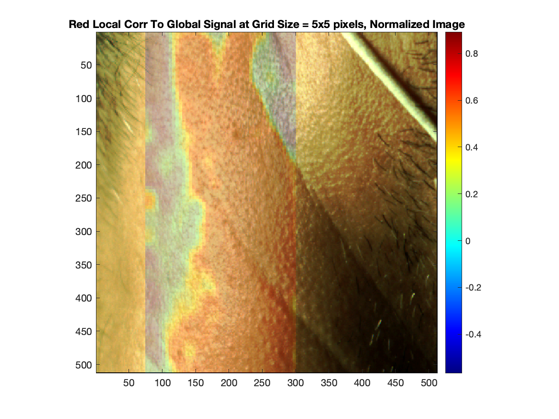 | 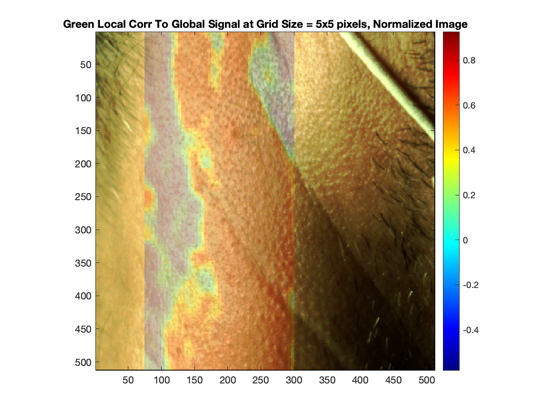 | 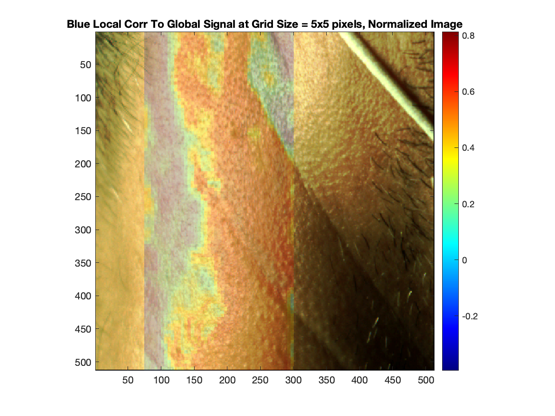 |
| --- | --- | --- |

Figure 2: Correlation of each local SVI (red on left, green in middle, blue on right) to the global SVI signal extracted from the ROI.

| 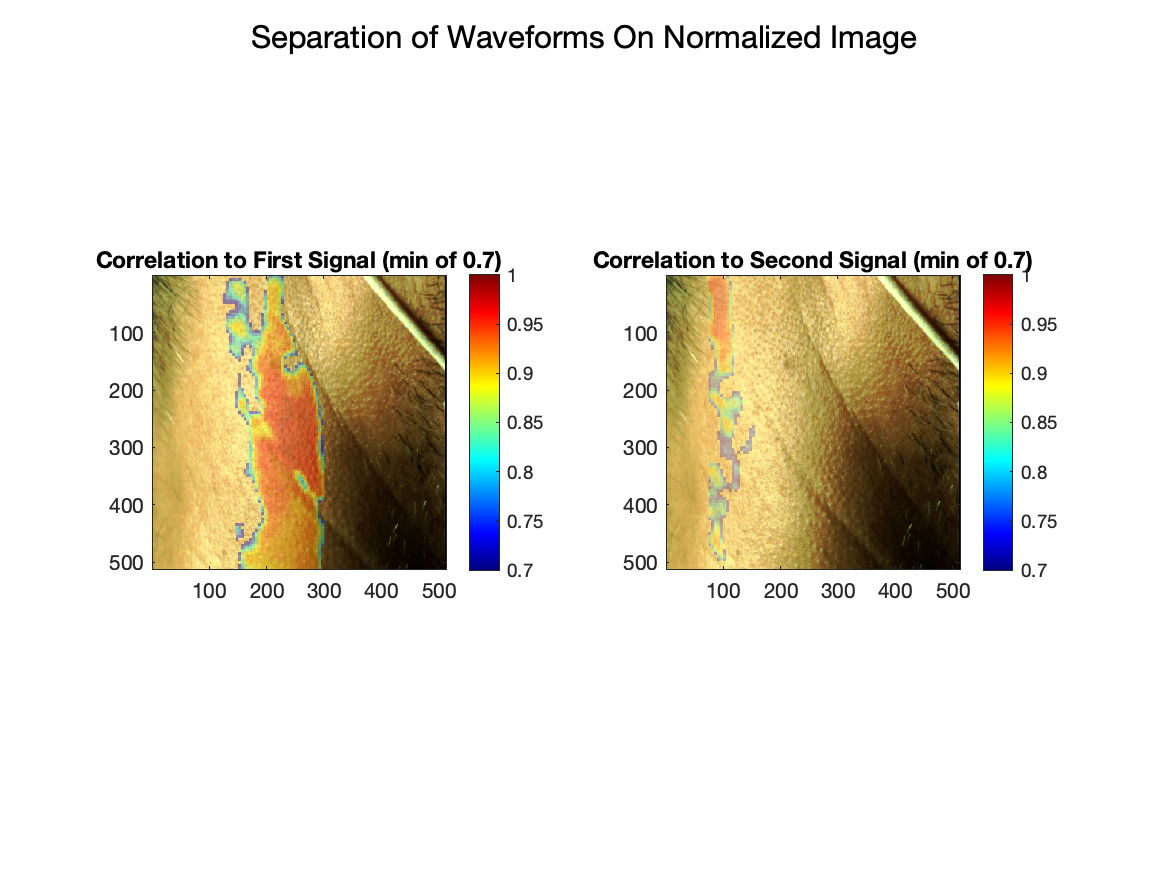 | 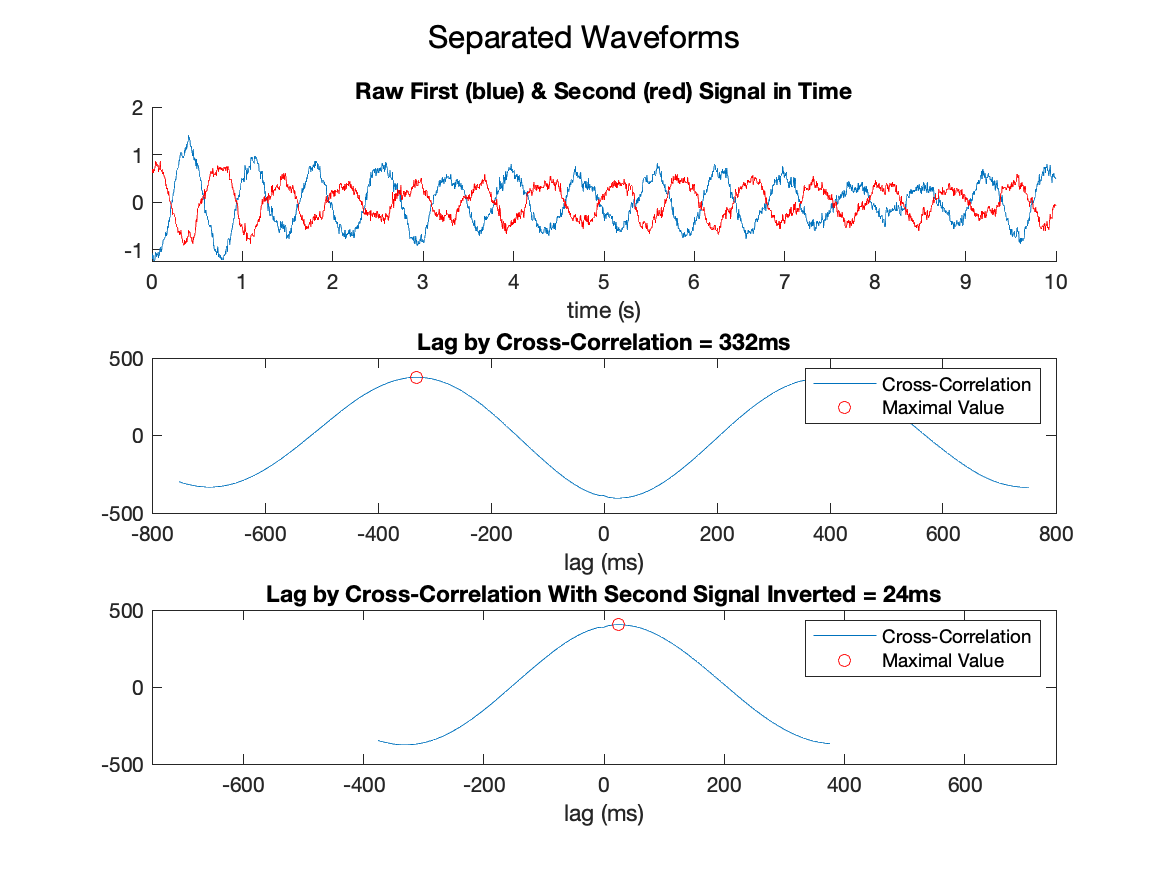 |
| --- | --- |
| 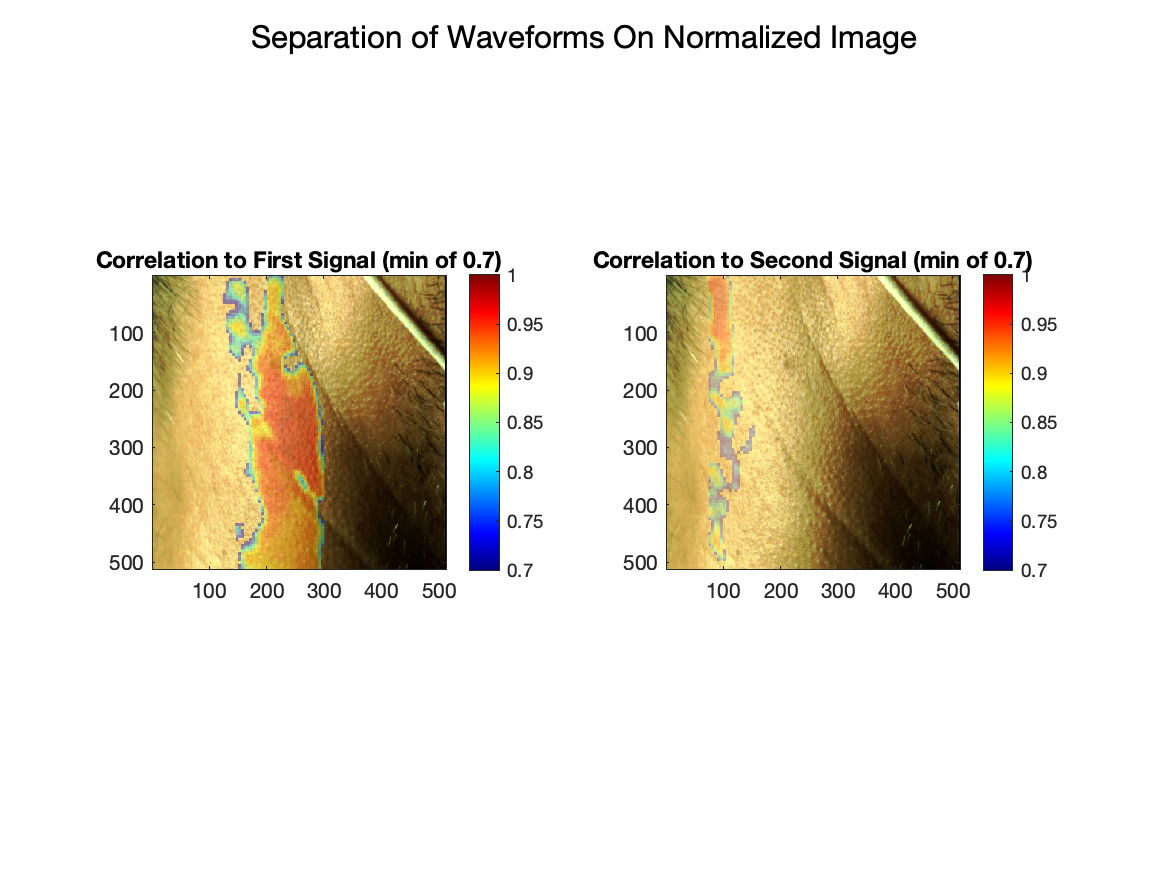 |  |

Figure 3: the first region generating local SVI signals with high internal correlation (top left), the second region generating local SVI signal with high internal correlation (top right), the SVI signals from the first and second regions plotted in the time domain and analyzed with cross-correlation (right).

## Subject 2, Signal A

| 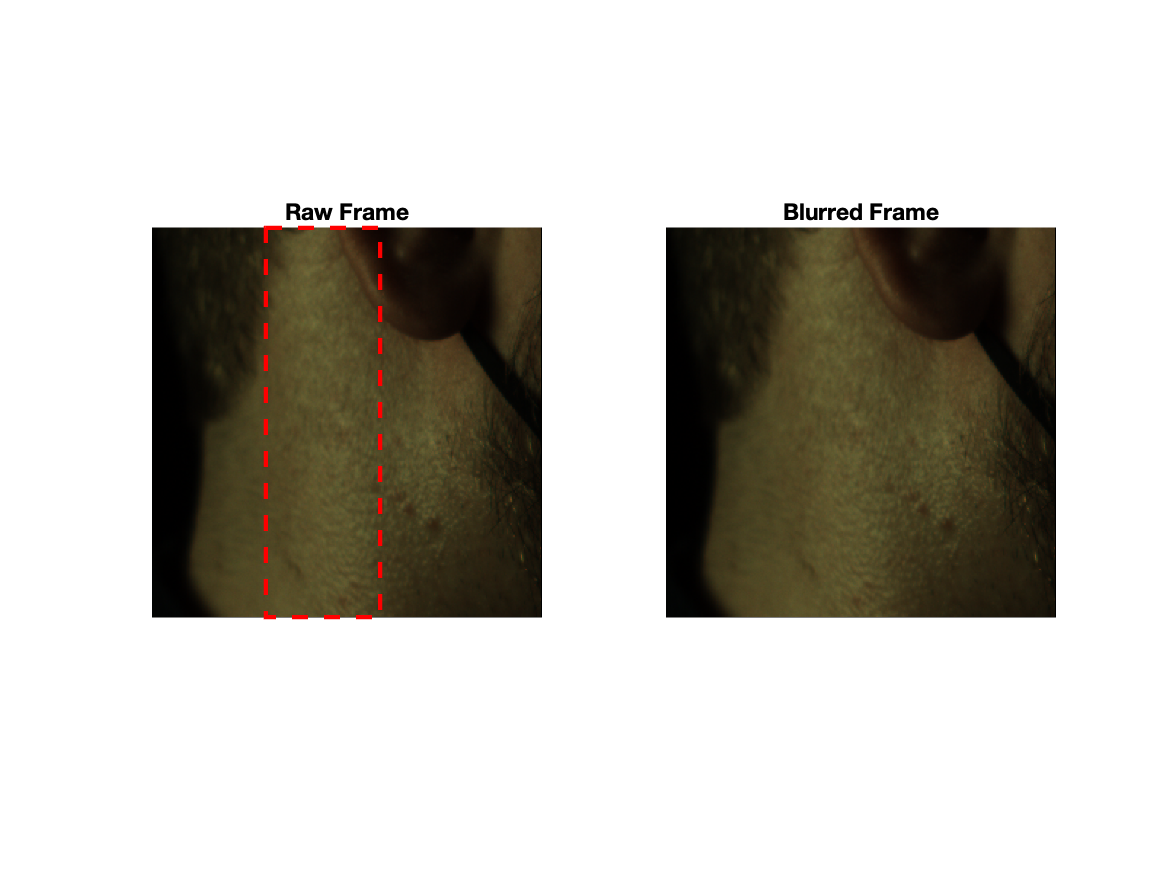 | 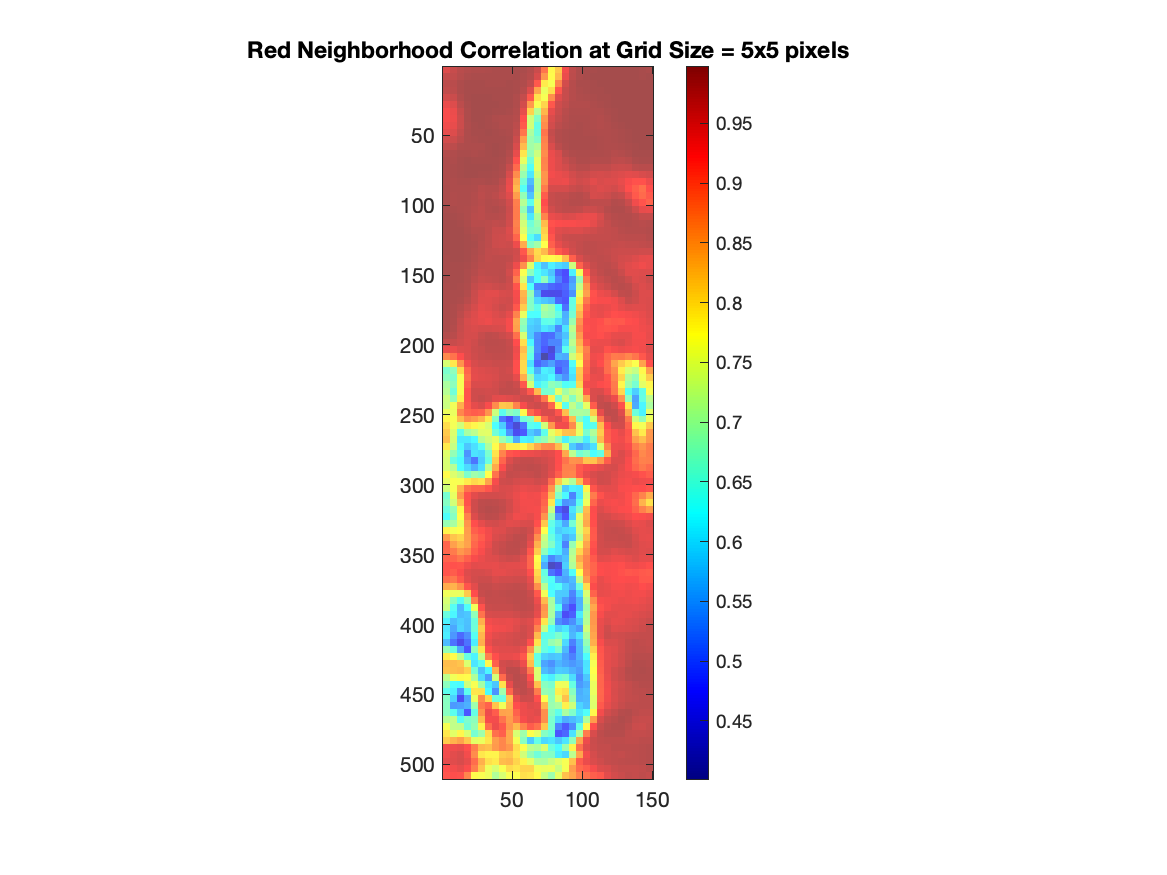 |
| --- | --- |

Figure 4: ROI (left) and local correlation analysis (right)

| 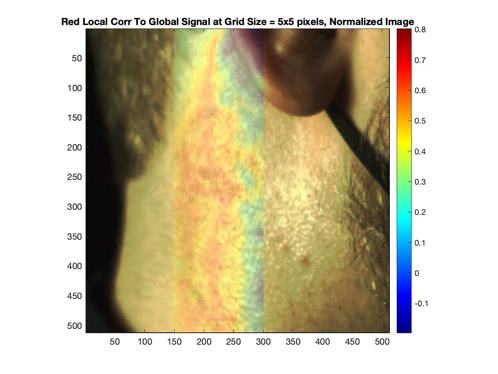 | 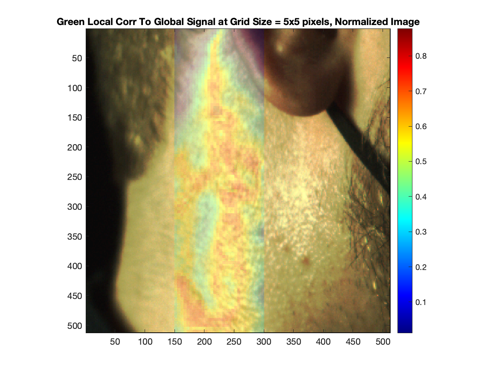 | 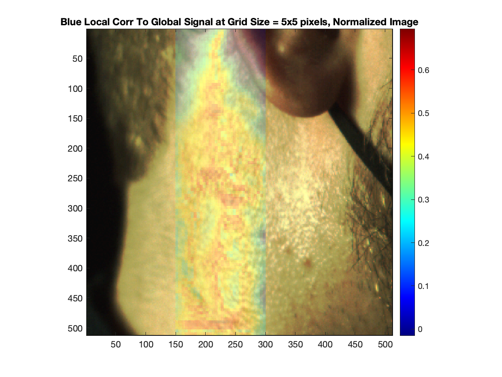 |
| --- | --- | --- |

Figure 5: Correlation of each local SVI (red on left, green in middle, blue on right) to the global SVI signal extracted from the ROI.

| 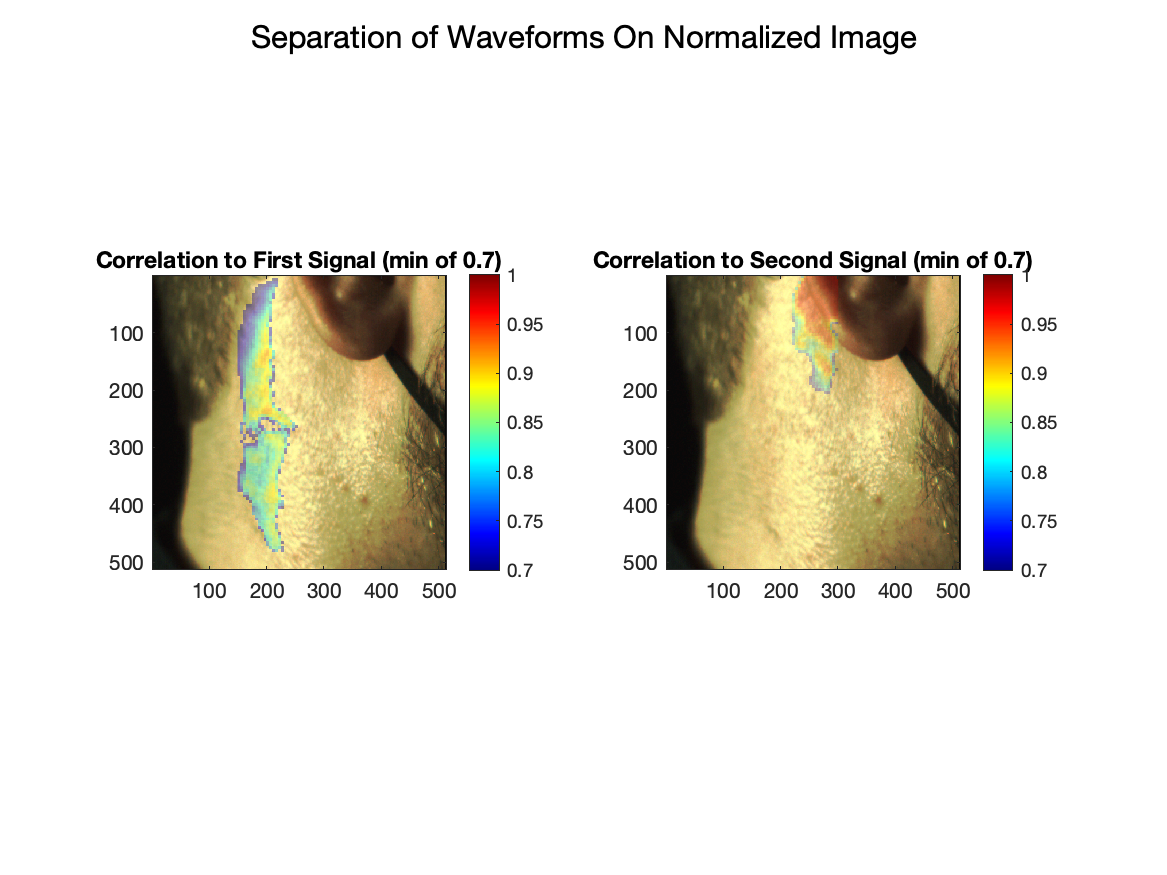 | 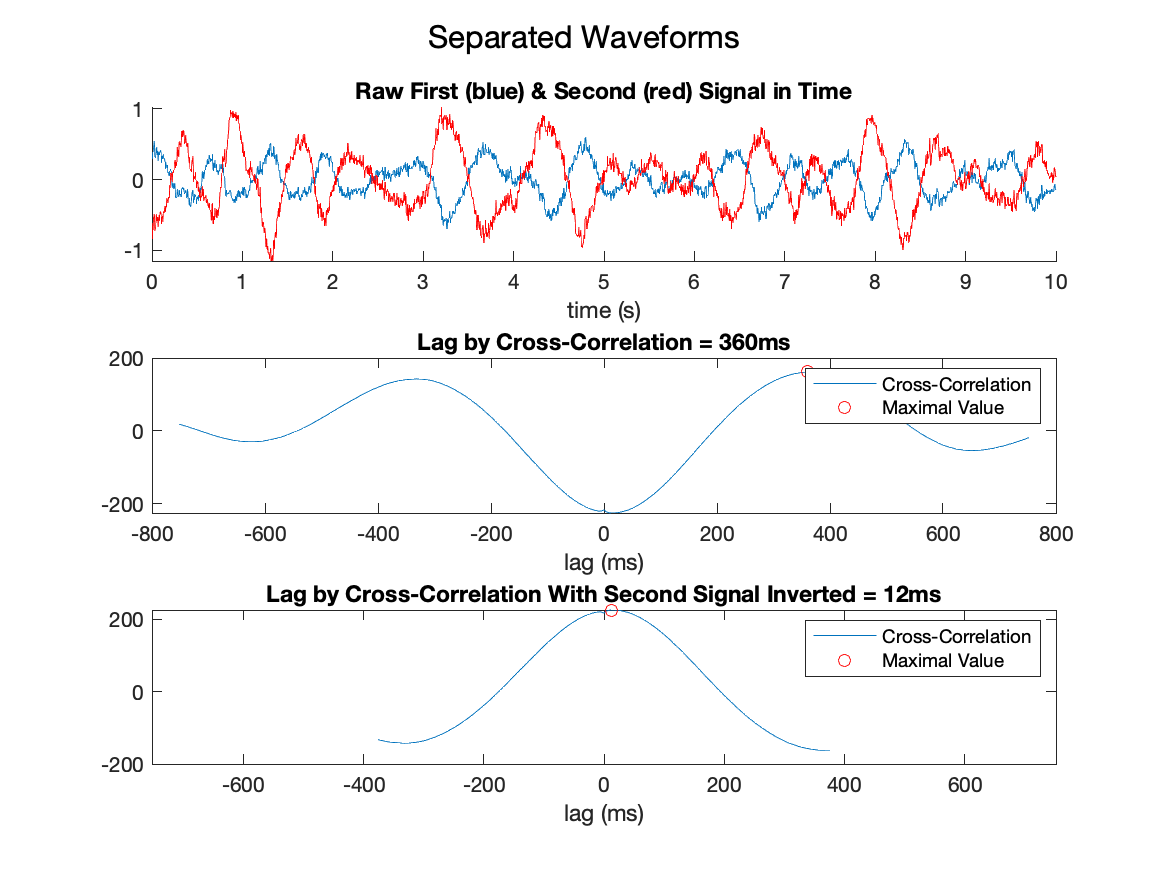 |
| --- | --- |
| 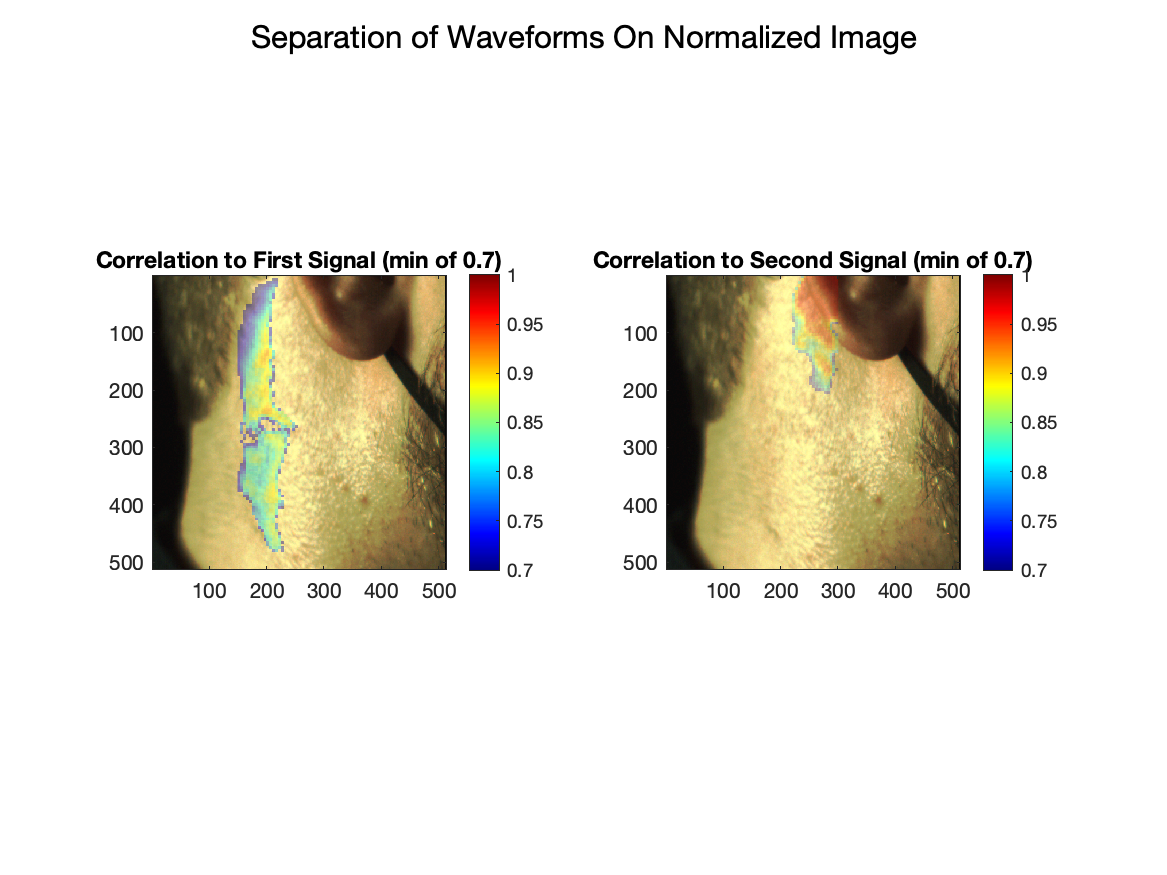 |  |

Figure 6: the first region generating local SVI signals with high internal correlation (top left), the second region generating local SVI signal with high internal correlation (top right), the SVI signals from the first and second regions plotted in the time domain and analyzed with cross-correlation (right).

## Subject 2, Signal B

| 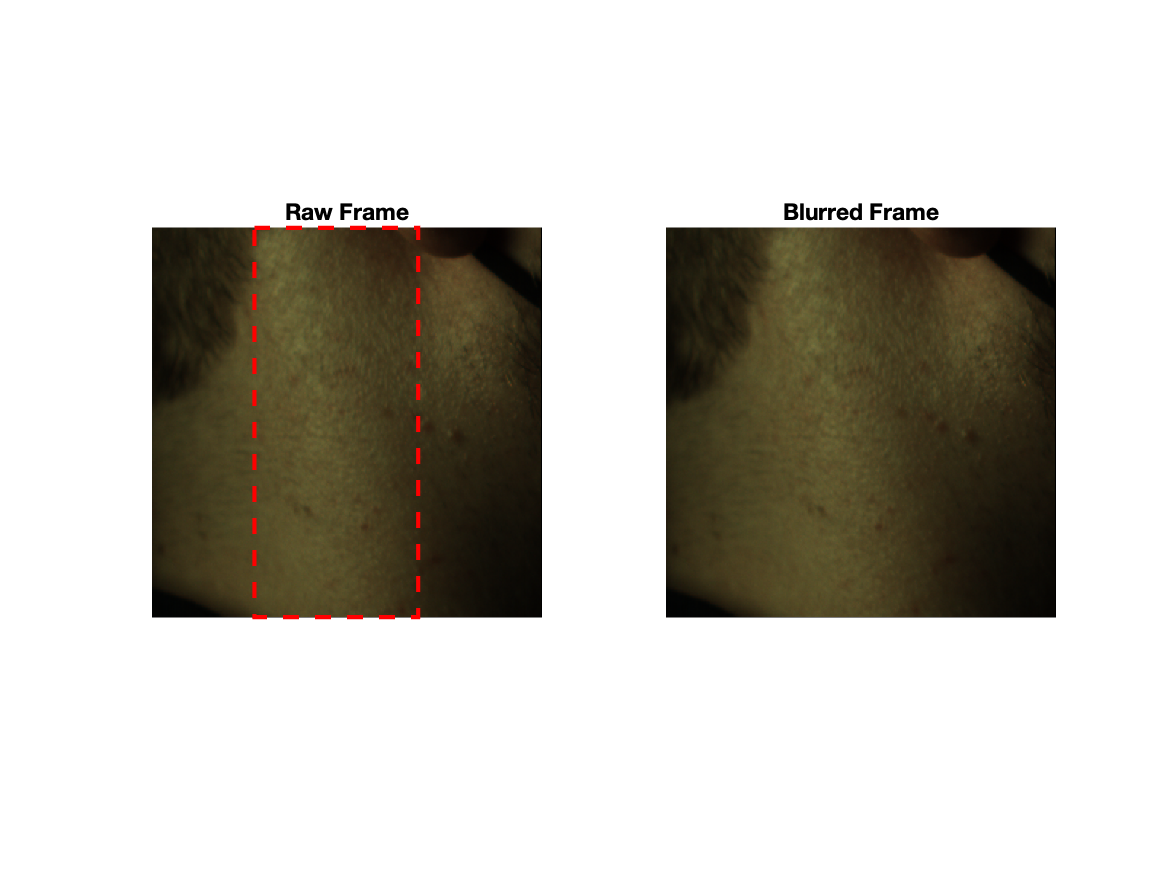 | 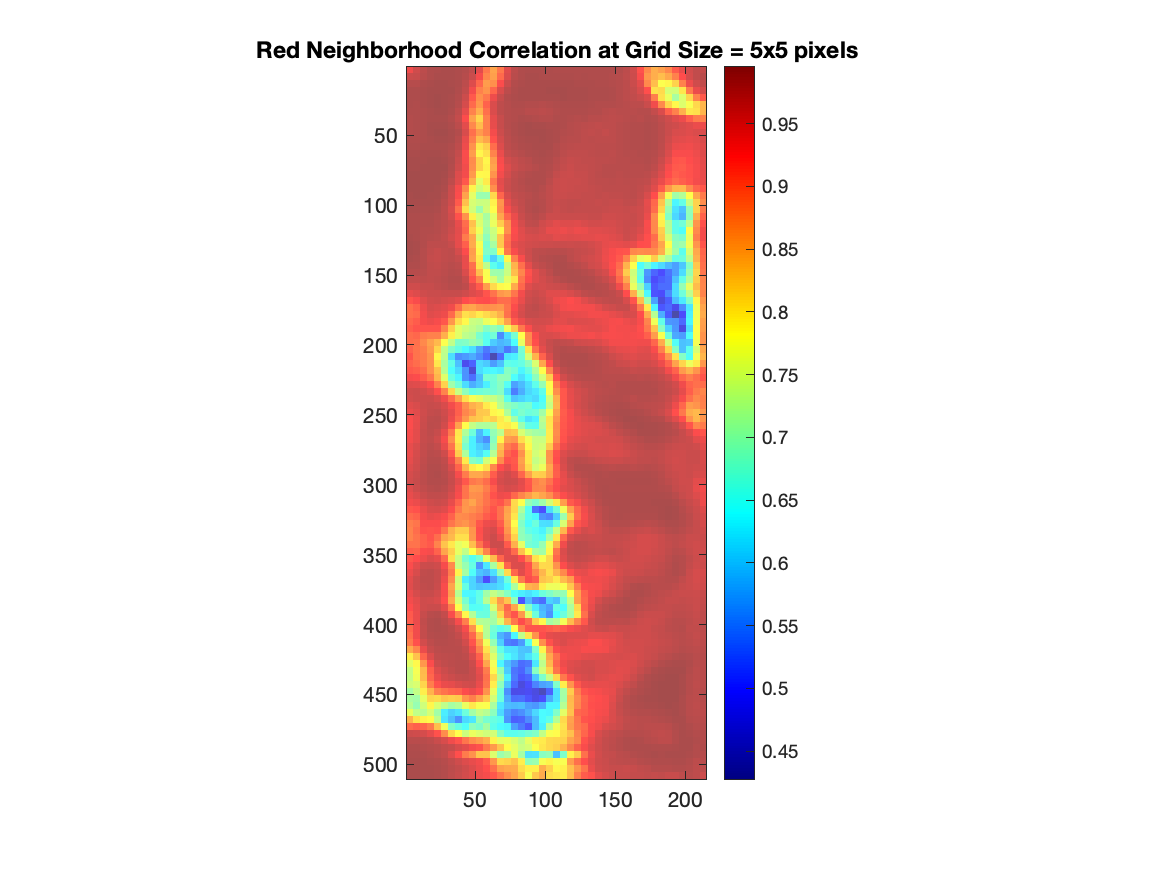 |
| --- | --- |

Figure 7: ROI (left) and local correlation analysis (right)

| 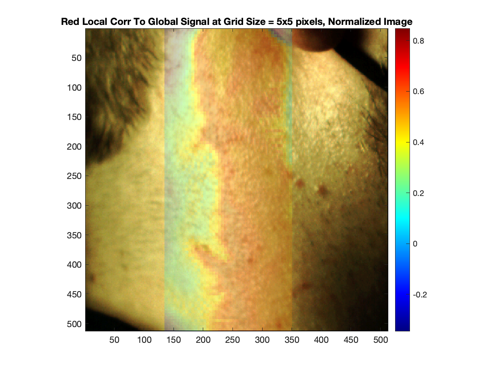 | 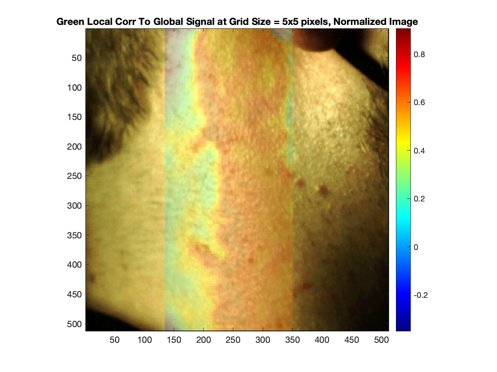 | 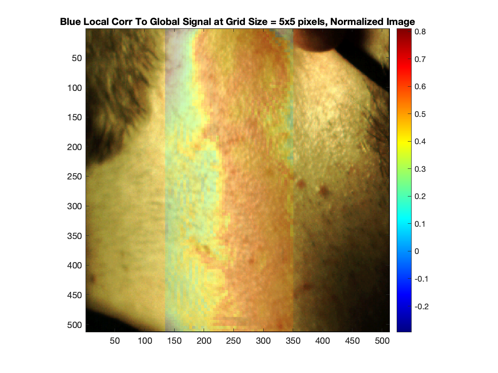 |
| --- | --- | --- |

Figure 8: Correlation of each local SVI (red on left, green in middle, blue on right) to the global SVI signal extracted from the ROI.

| 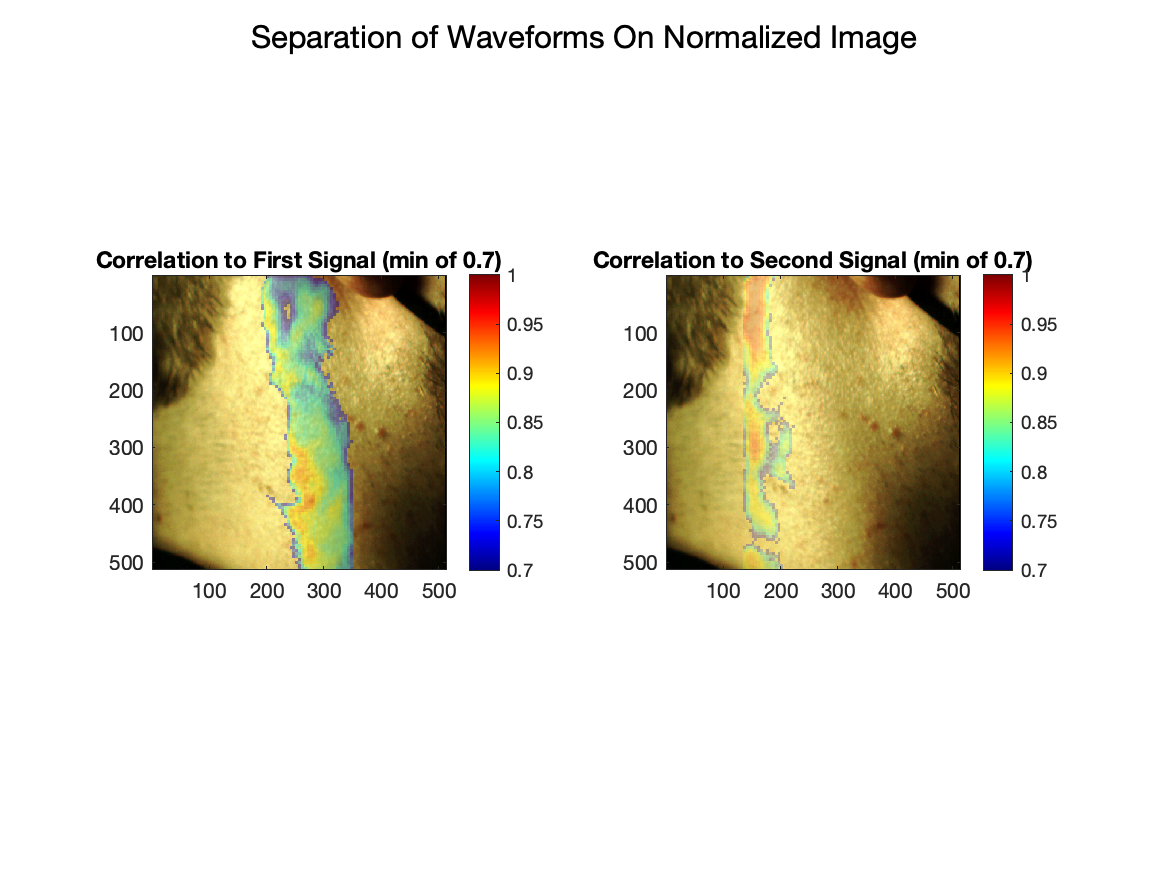 | 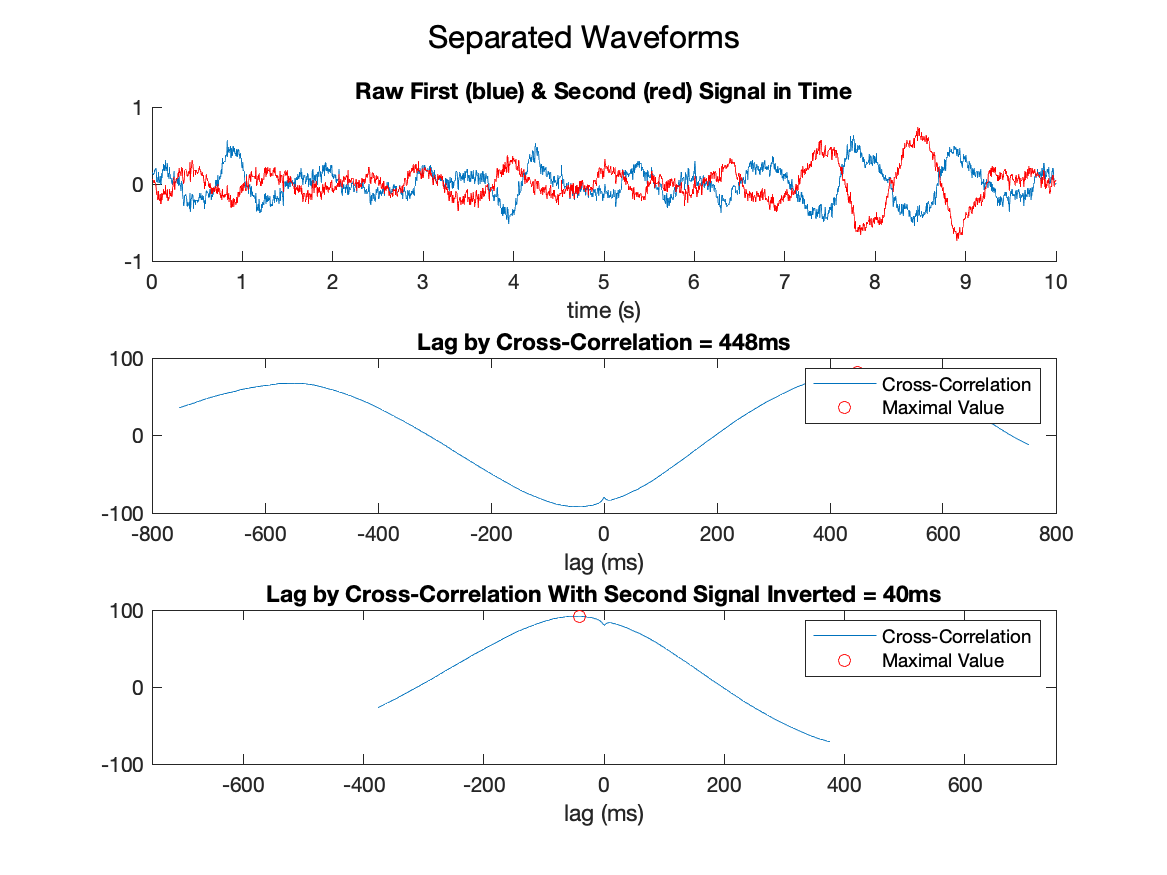 |
| --- | --- |
| 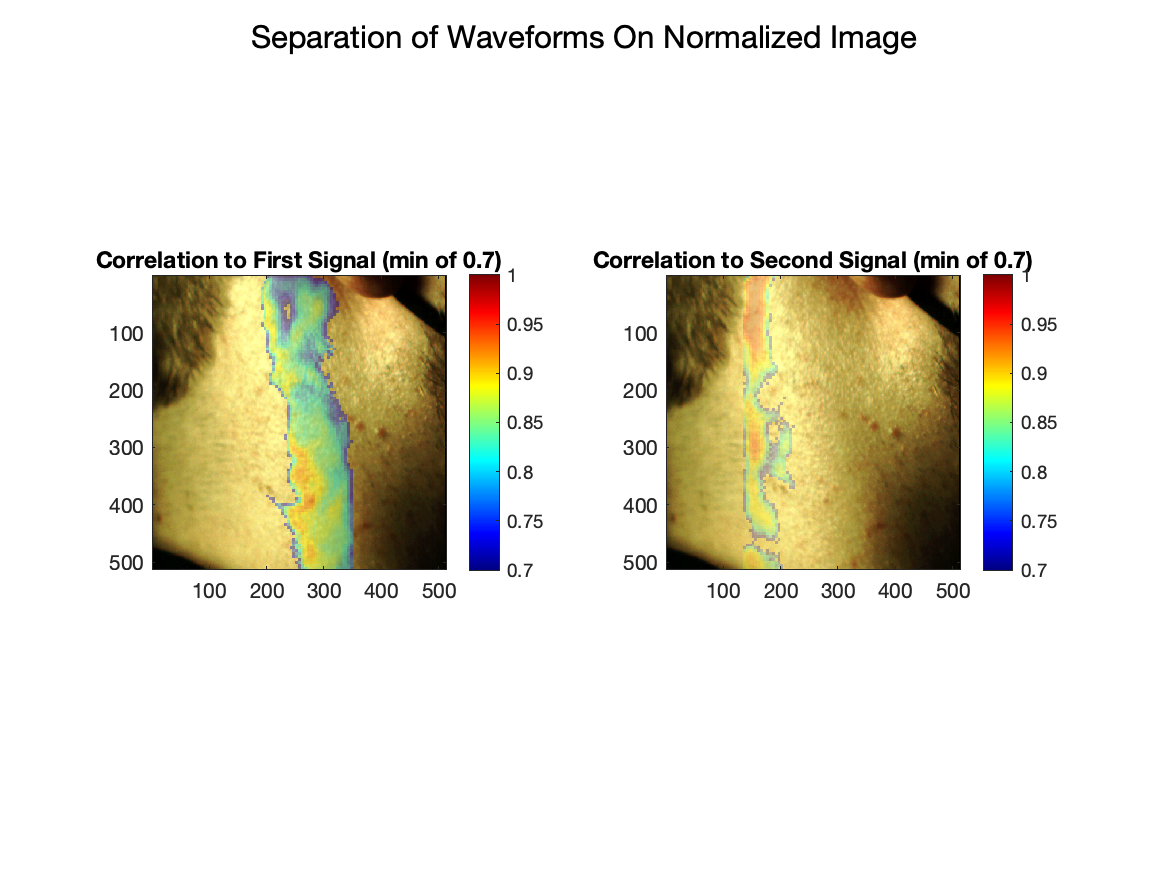 |  |

Figure 9: the first region generating local SVI signals with high internal correlation (top left), the second region generating local SVI signal with high internal correlation (top right), the SVI signals from the first and second regions plotted in the time domain and analyzed with cross-correlation (right).

## Subject 3, Signal A

| 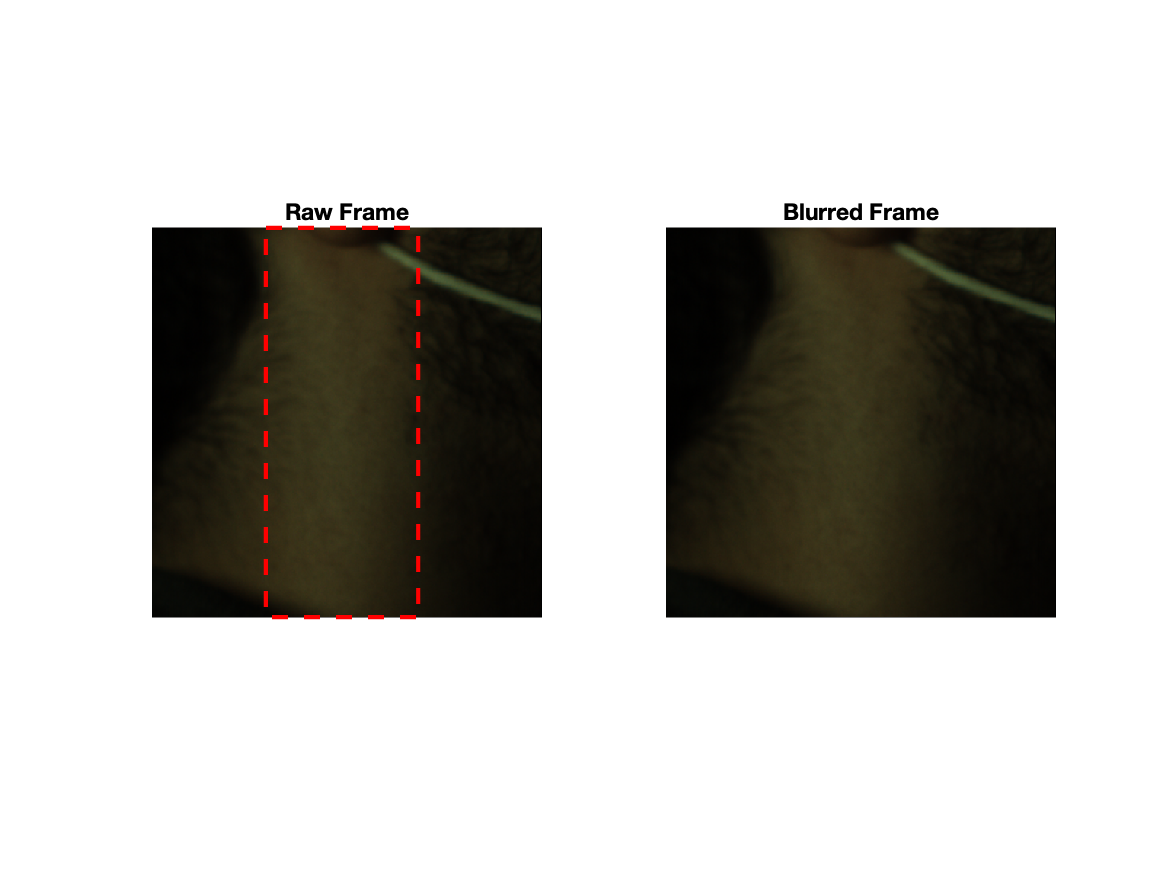 | 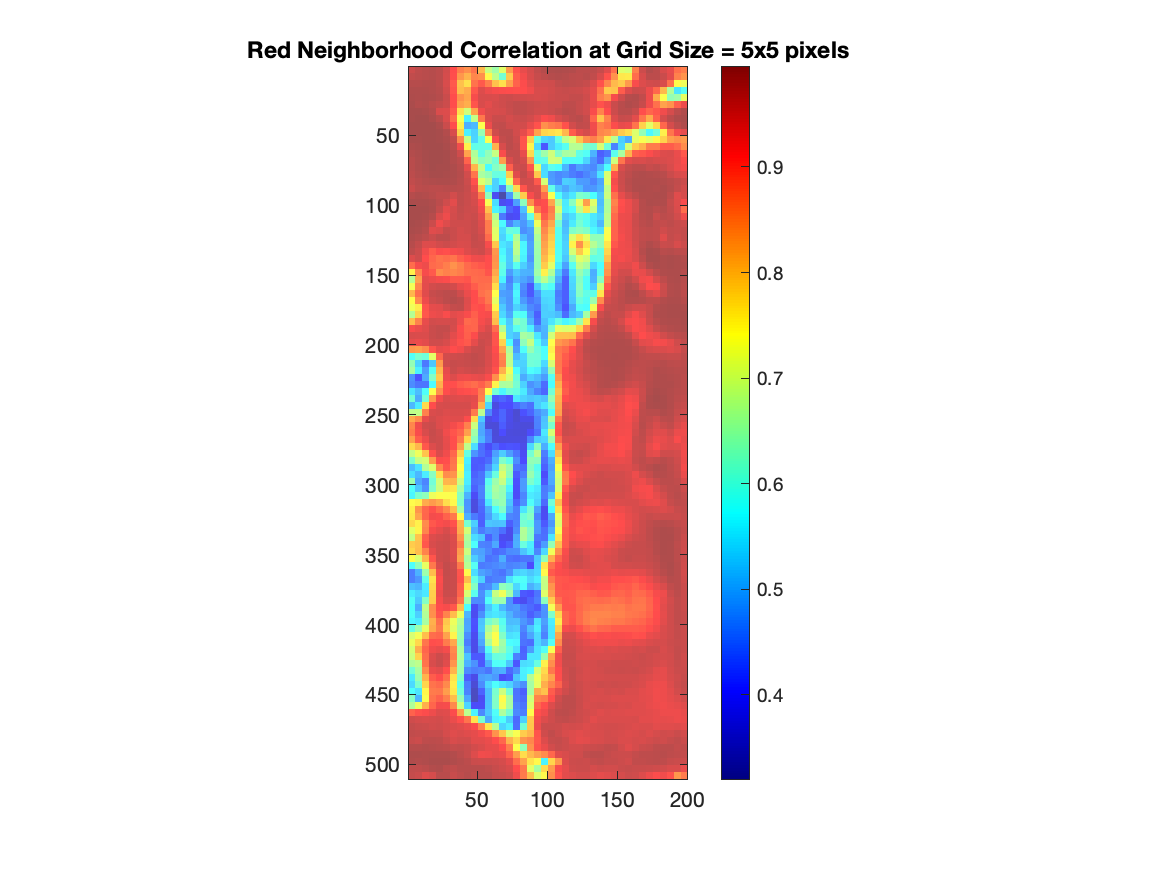 |
| --- | --- |

Figure 10: ROI (left) and local correlation analysis (right)

| 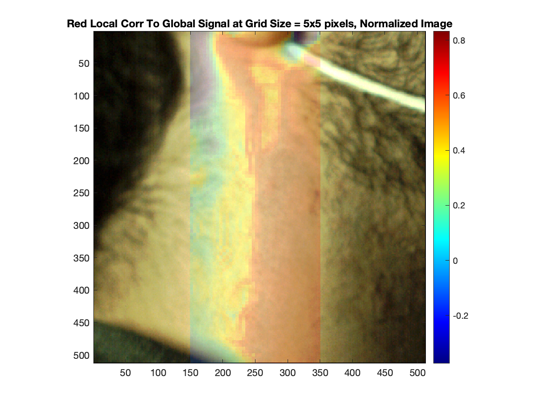 | 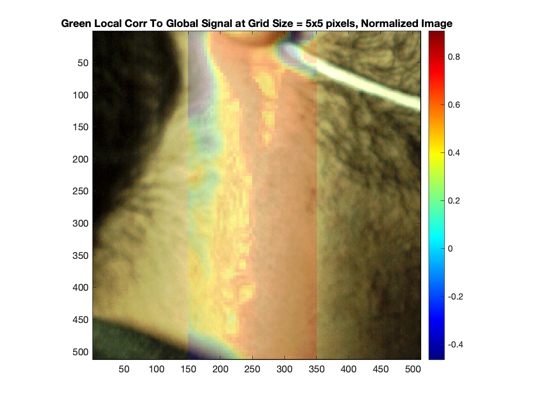 | 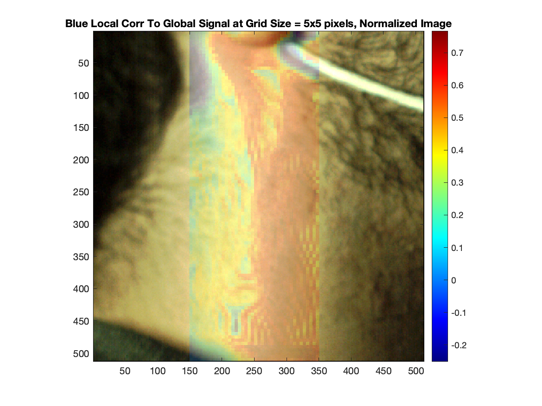 |
| --- | --- | --- |

Figure 11: Correlation of each local SVI (red on left, green in middle, blue on right) to the global SVI signal extracted from the ROI.

| 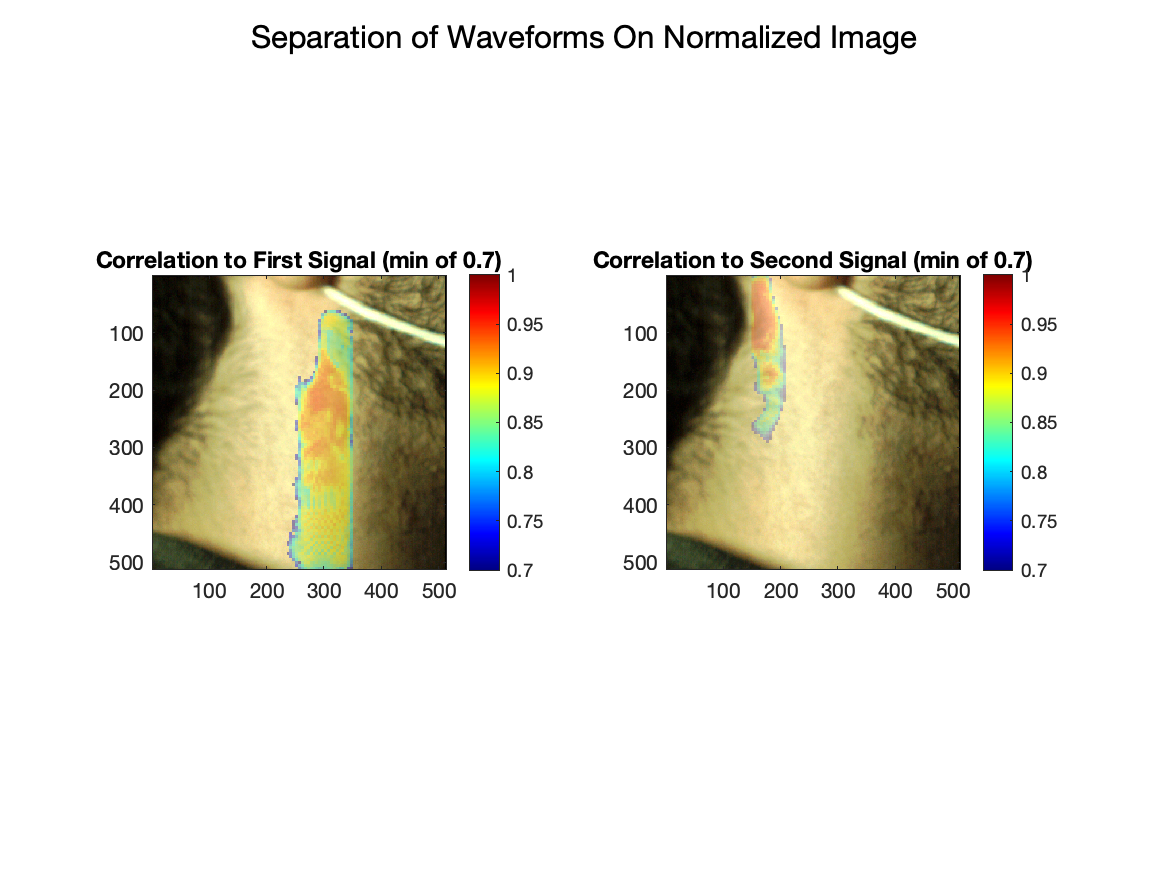 | 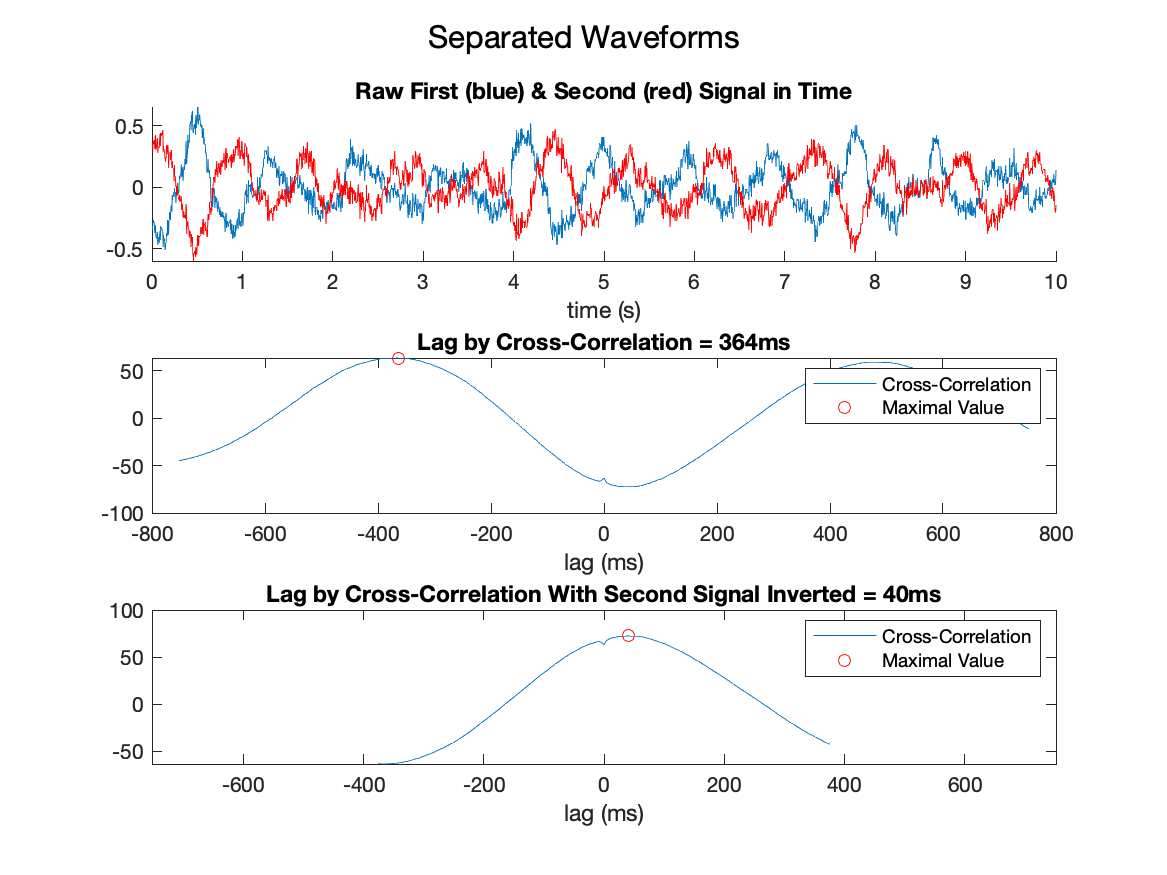 |
| --- | --- |
| 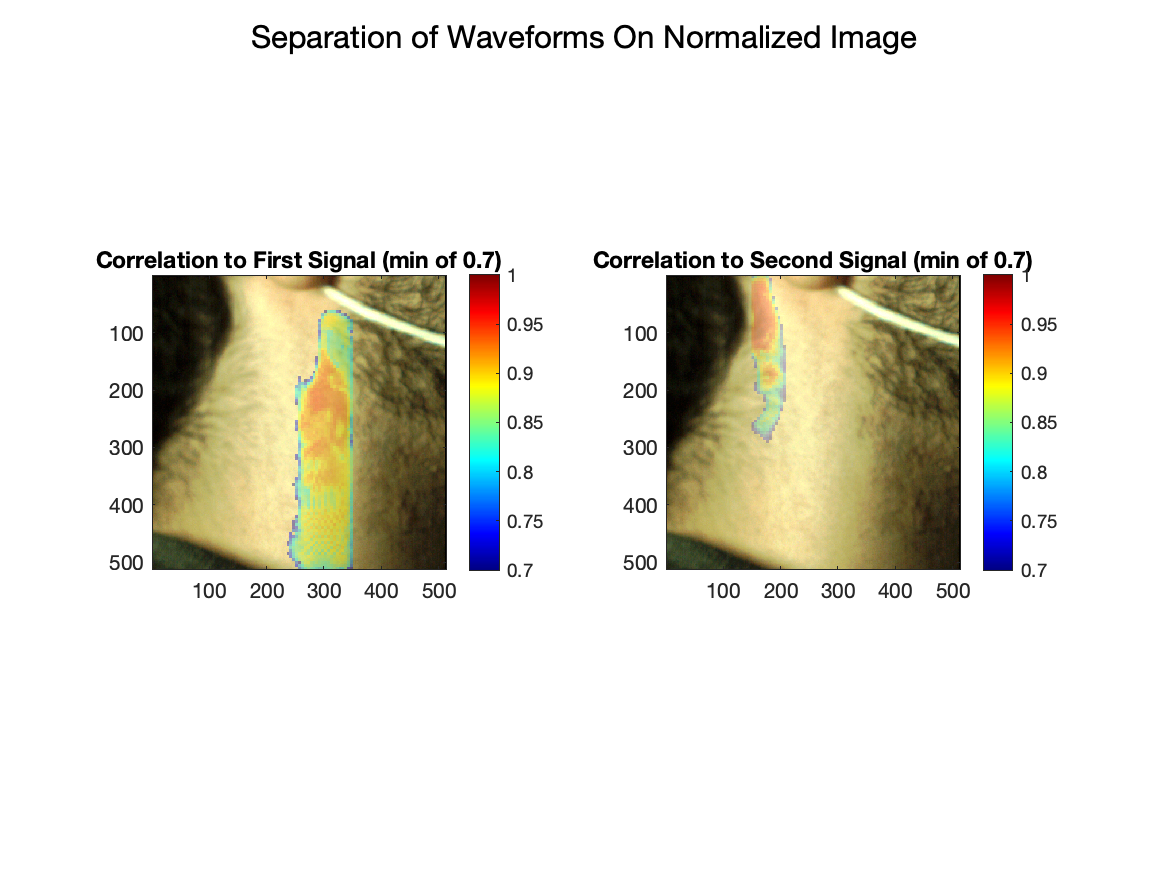 |  |

Figure 12: the first region generating local SVI signals with high internal correlation (top left), the second region generating local SVI signal with high internal correlation (top right), the SVI signals from the first and second regions plotted in the time domain and analyzed with cross-correlation (right).

## Subject 3, Signal B

| 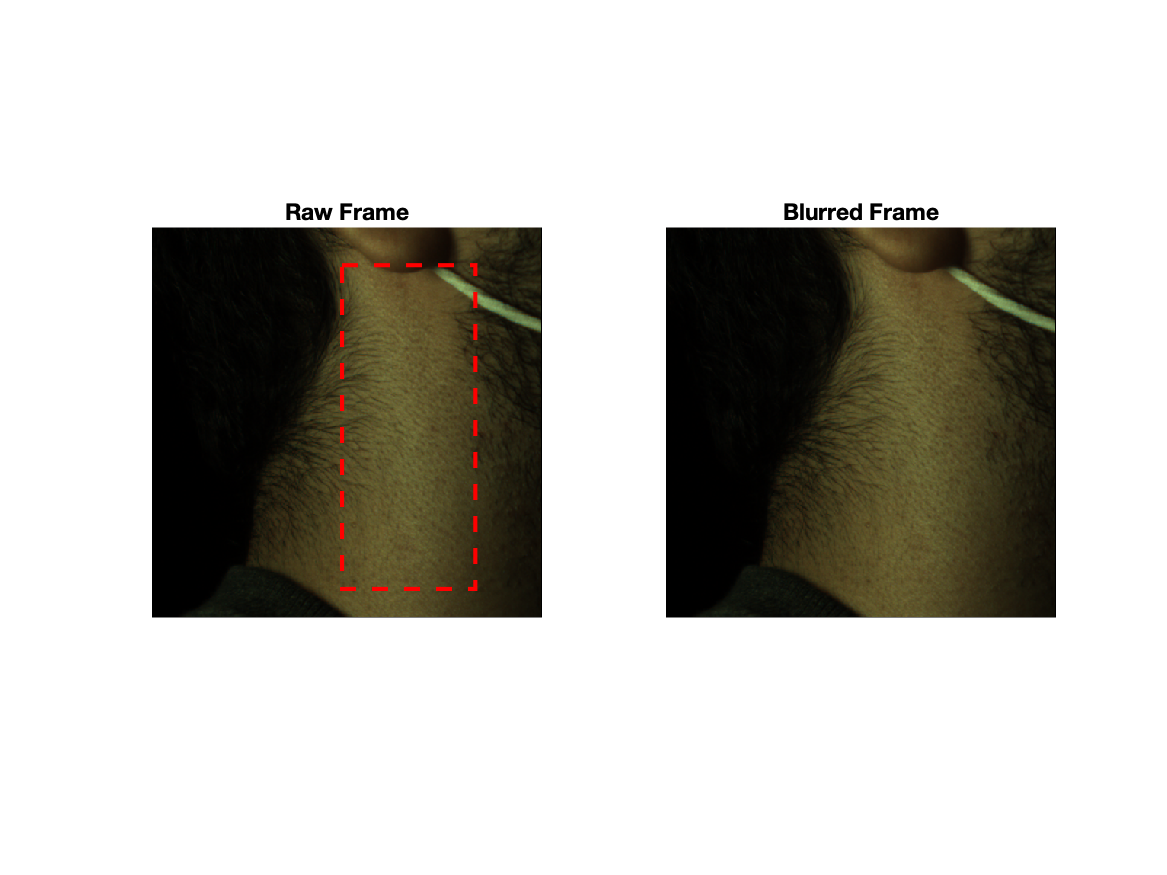 | 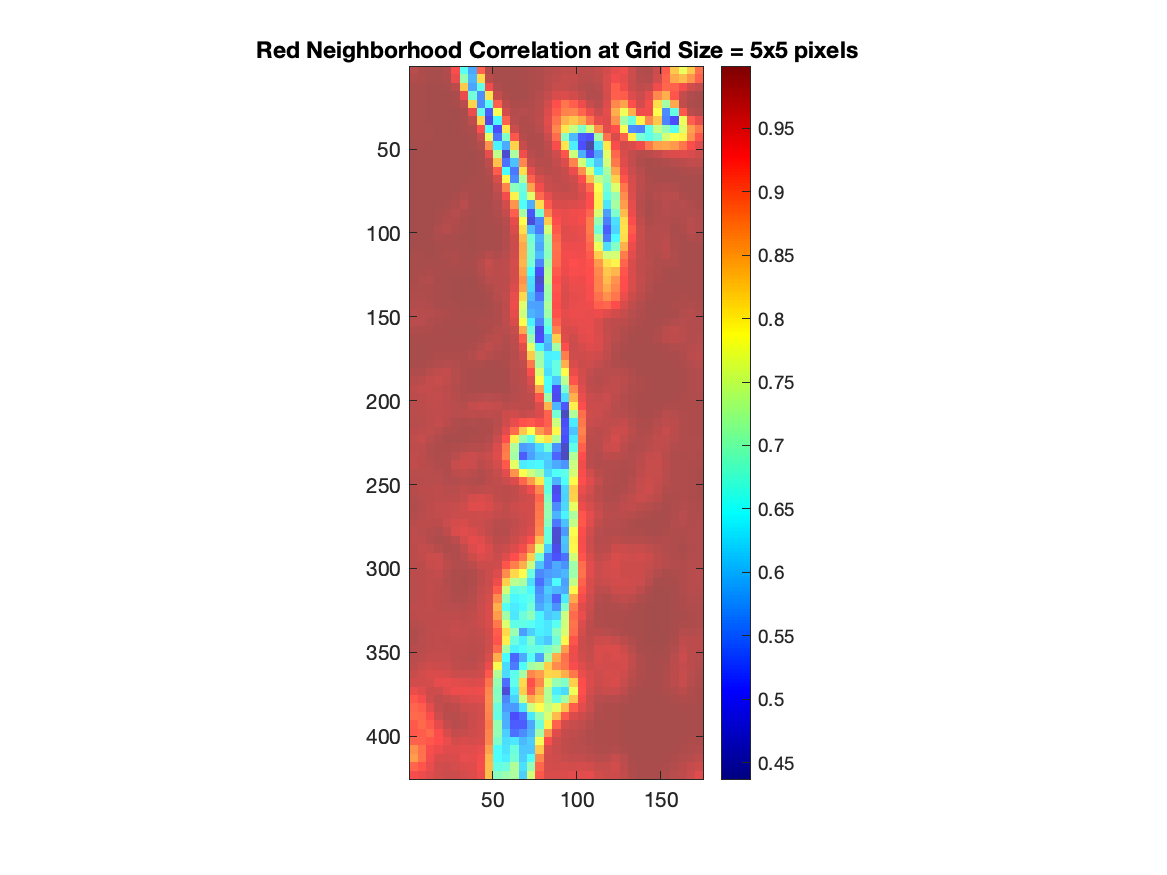 |
| --- | --- |

Figure 13: ROI (left) and local correlation analysis (right)

| 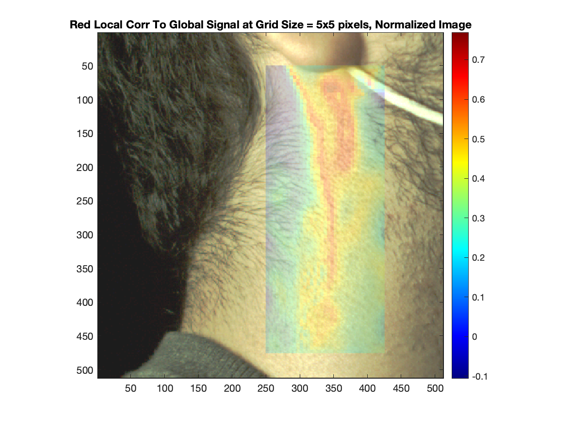 | 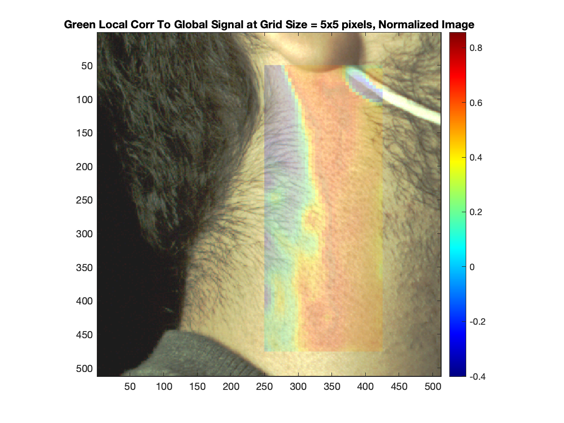 | 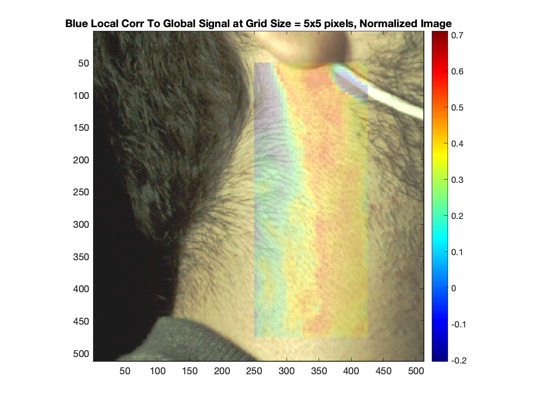 |
| --- | --- | --- |

Figure 14: Correlation of each local SVI (red on left, green in middle, blue on right) to the global SVI signal extracted from the ROI.

| 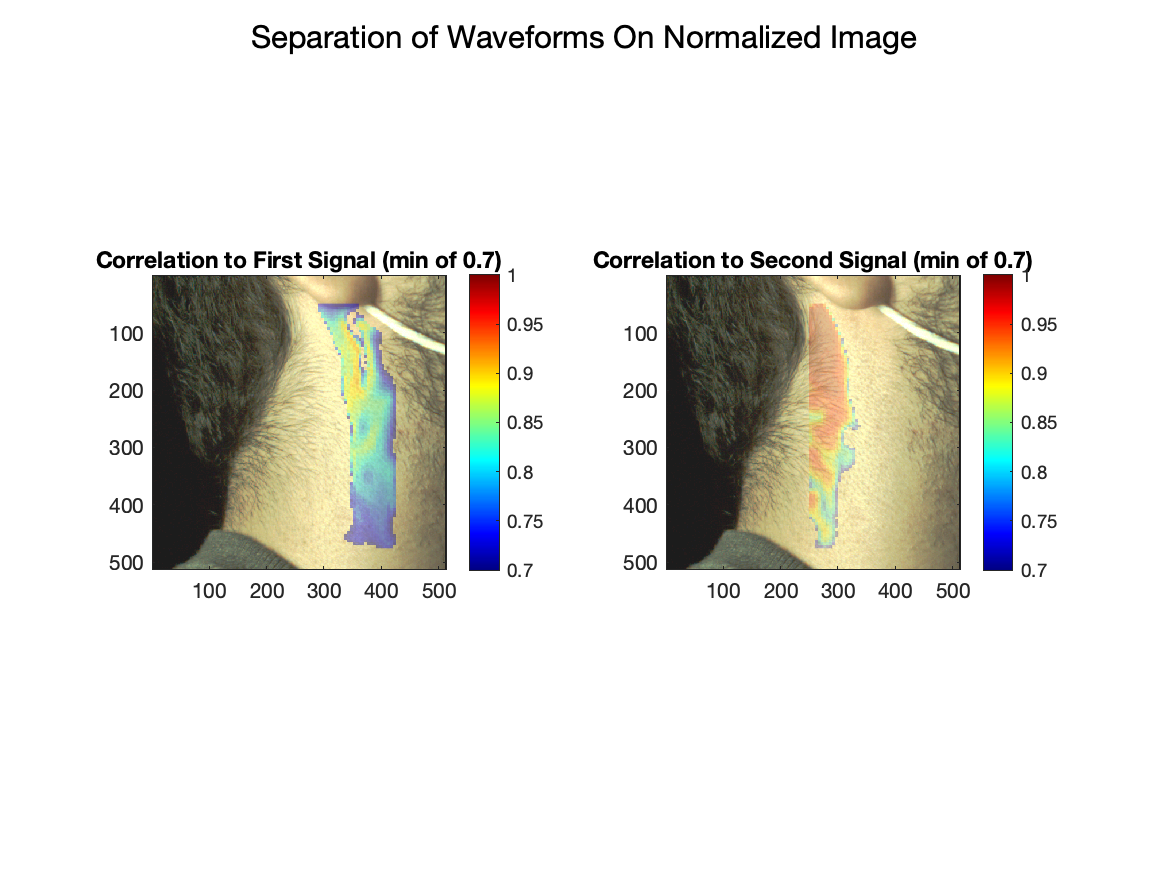 | 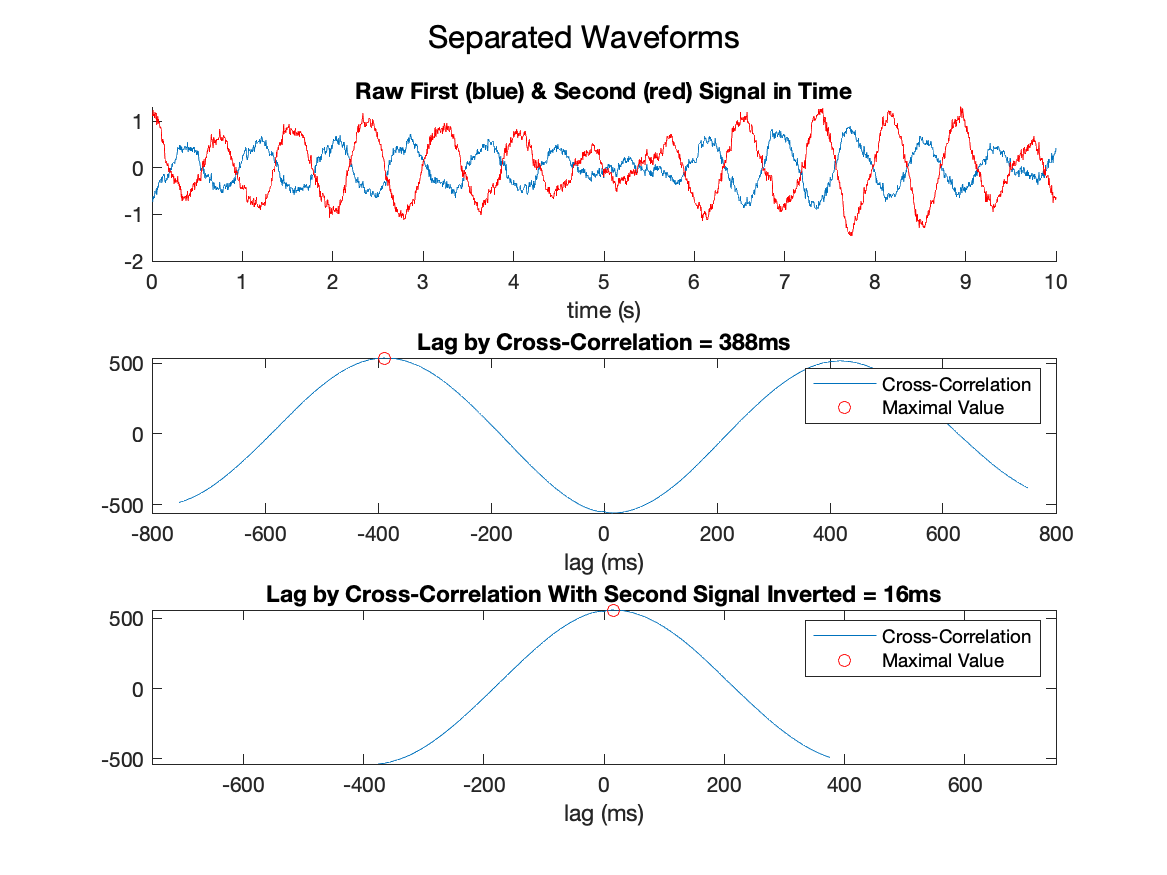 |
| --- | --- |
| 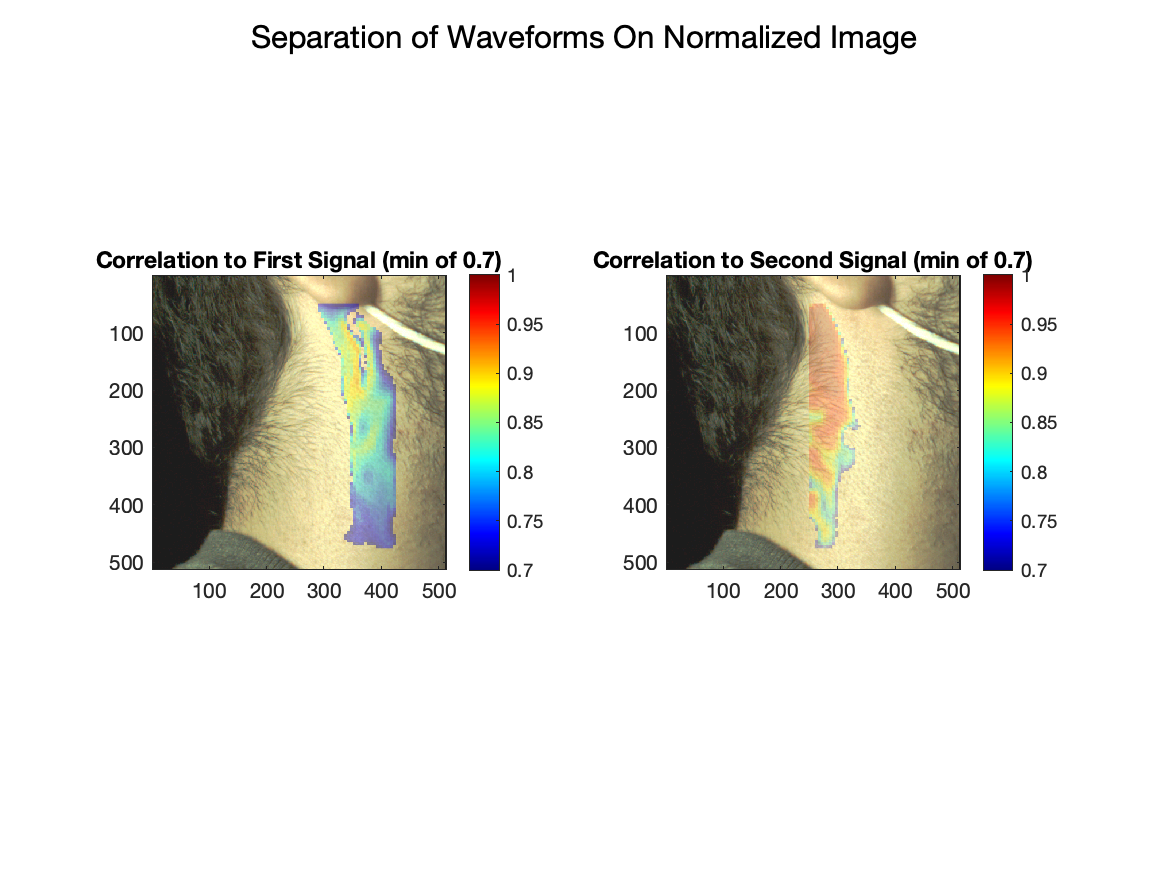 |  |

Figure 15: the first region generating local SVI signals with high internal correlation (top left), the second region generating local SVI signal with high internal correlation (top right), the SVI signals from the first and second regions plotted in the time domain and analyzed with cross-correlation (right).

## Subject 4, Signal A

| 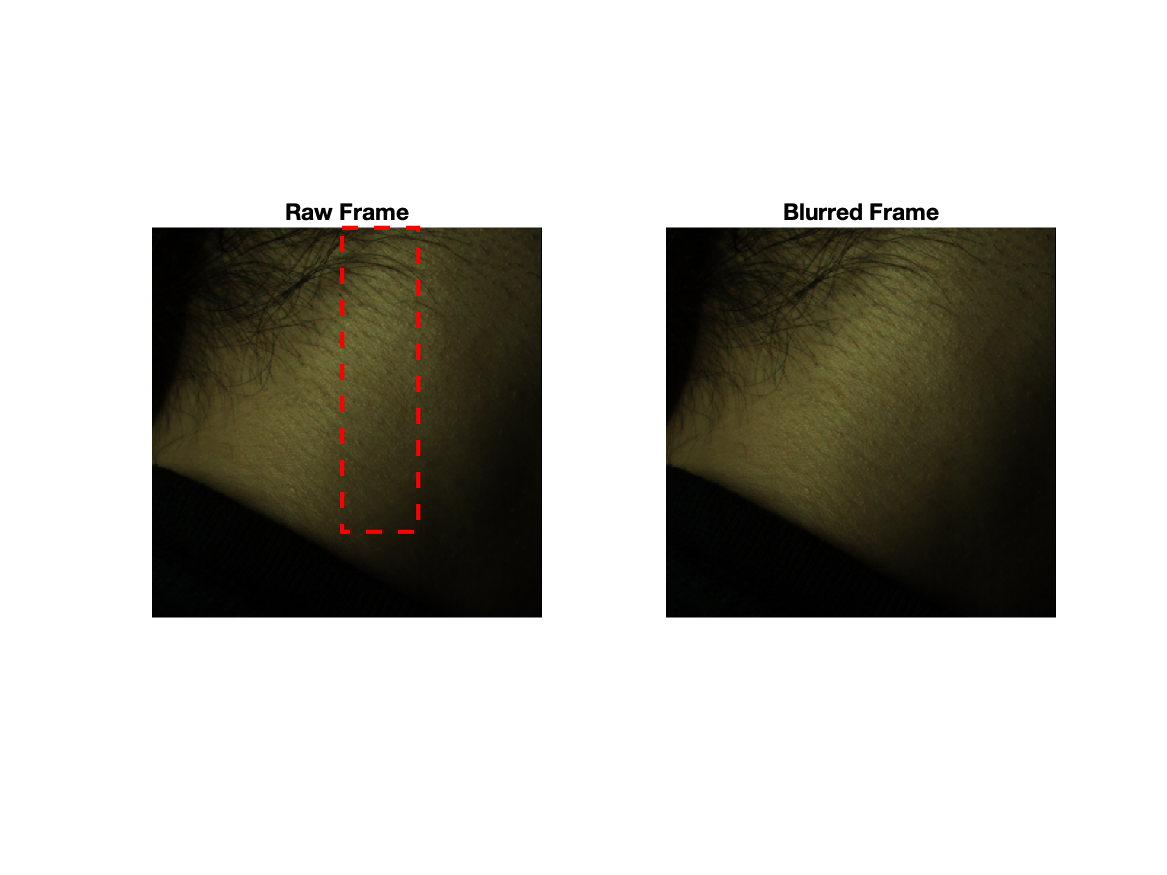 | 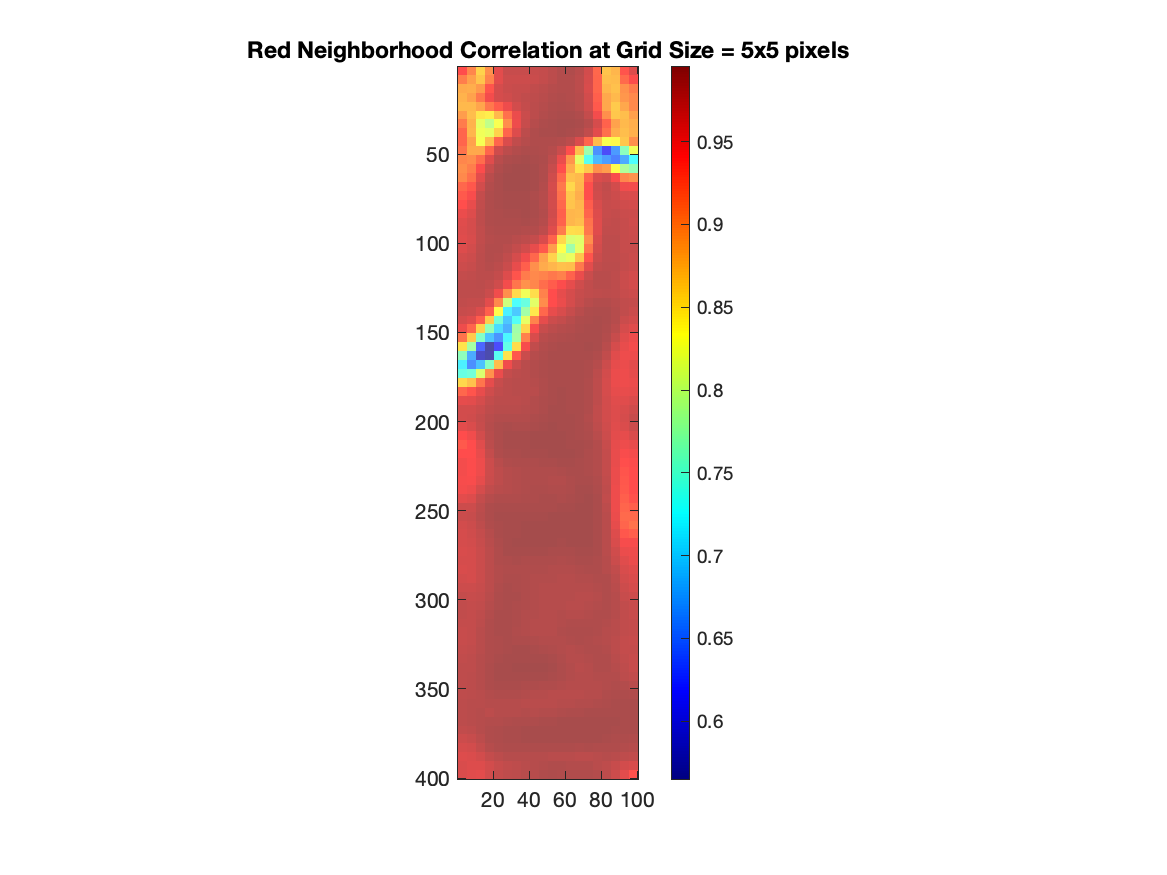 |
| --- | --- |

Figure 16: ROI (left) and local correlation analysis (right)

| 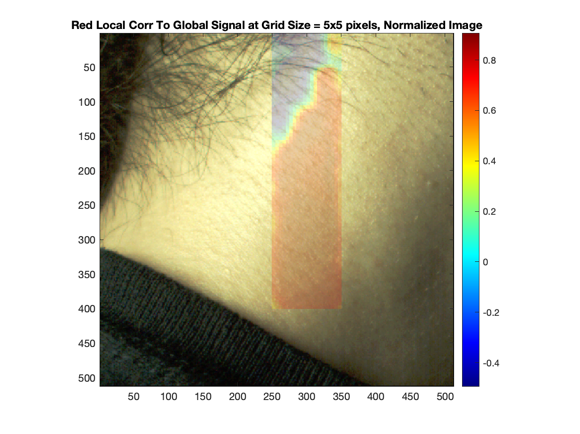 | 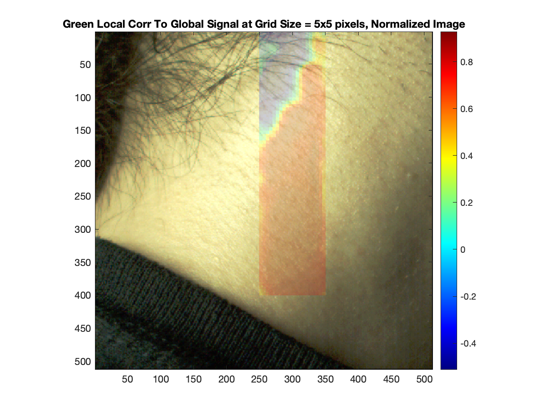 | 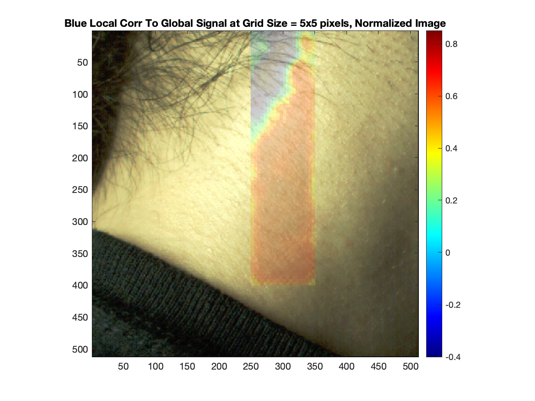 |
| --- | --- | --- |

Figure 17: Correlation of each local SVI (red on left, green in middle, blue on right) to the global SVI signal extracted from the ROI.

| 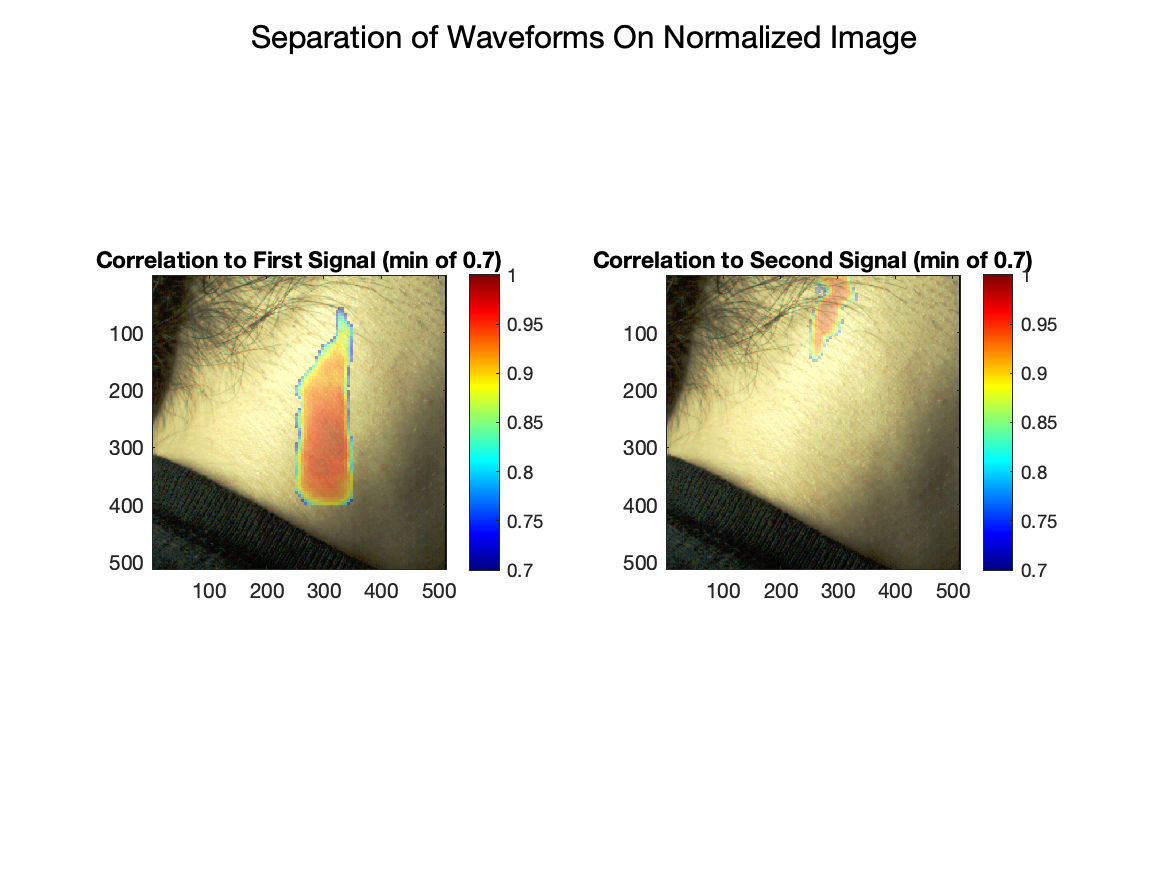 | 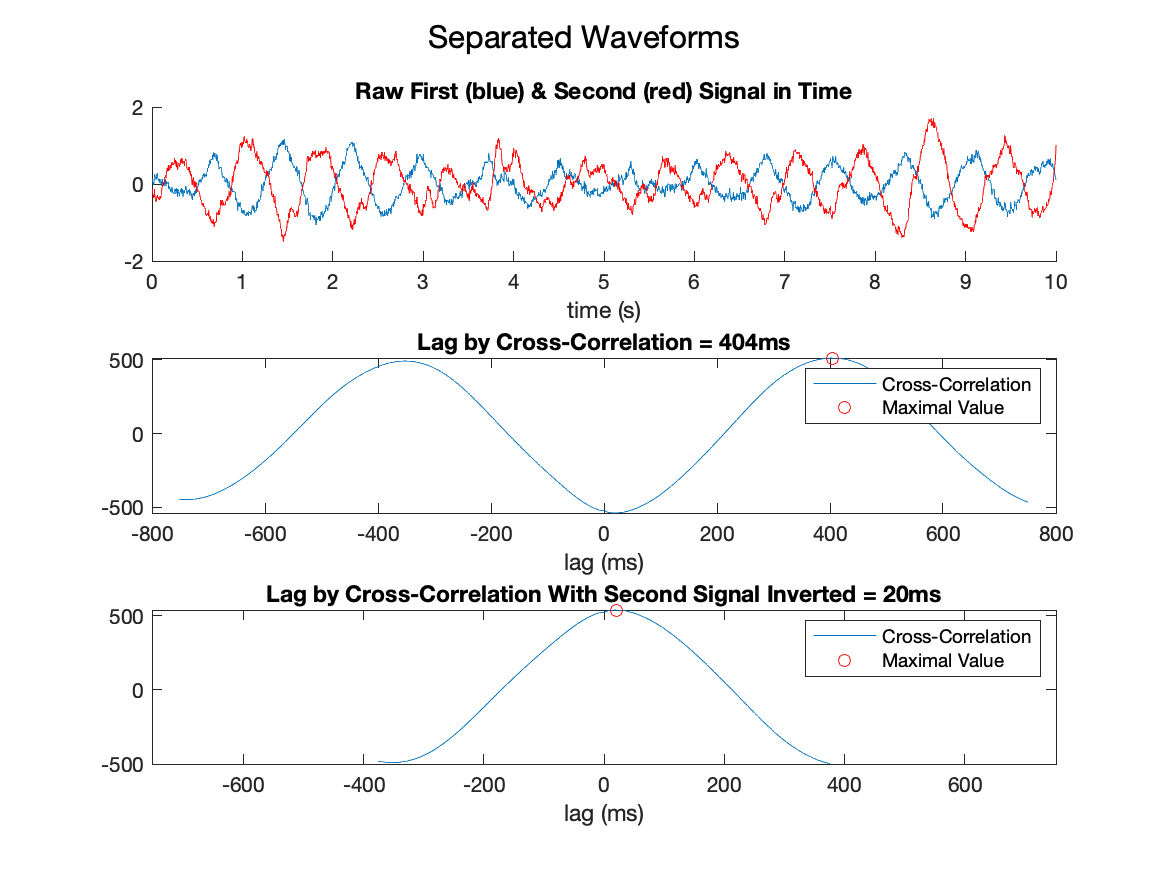 |
| --- | --- |
| 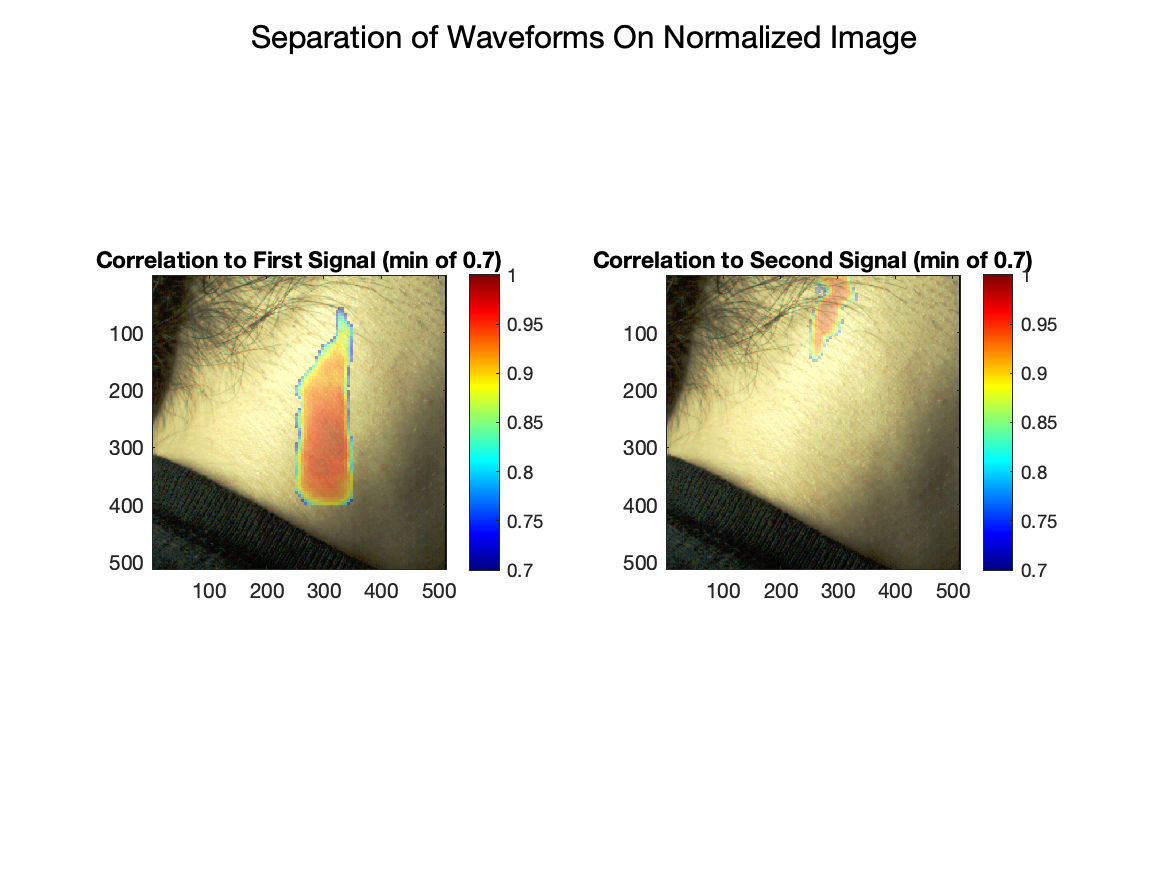 |  |

Figure 18: the first region generating local SVI signals with high internal correlation (top left), the second region generating local SVI signal with high internal correlation (top right), the SVI signals from the first and second regions plotted in the time domain and analyzed with cross-correlation (right).

## Subject 4, Signal B

| 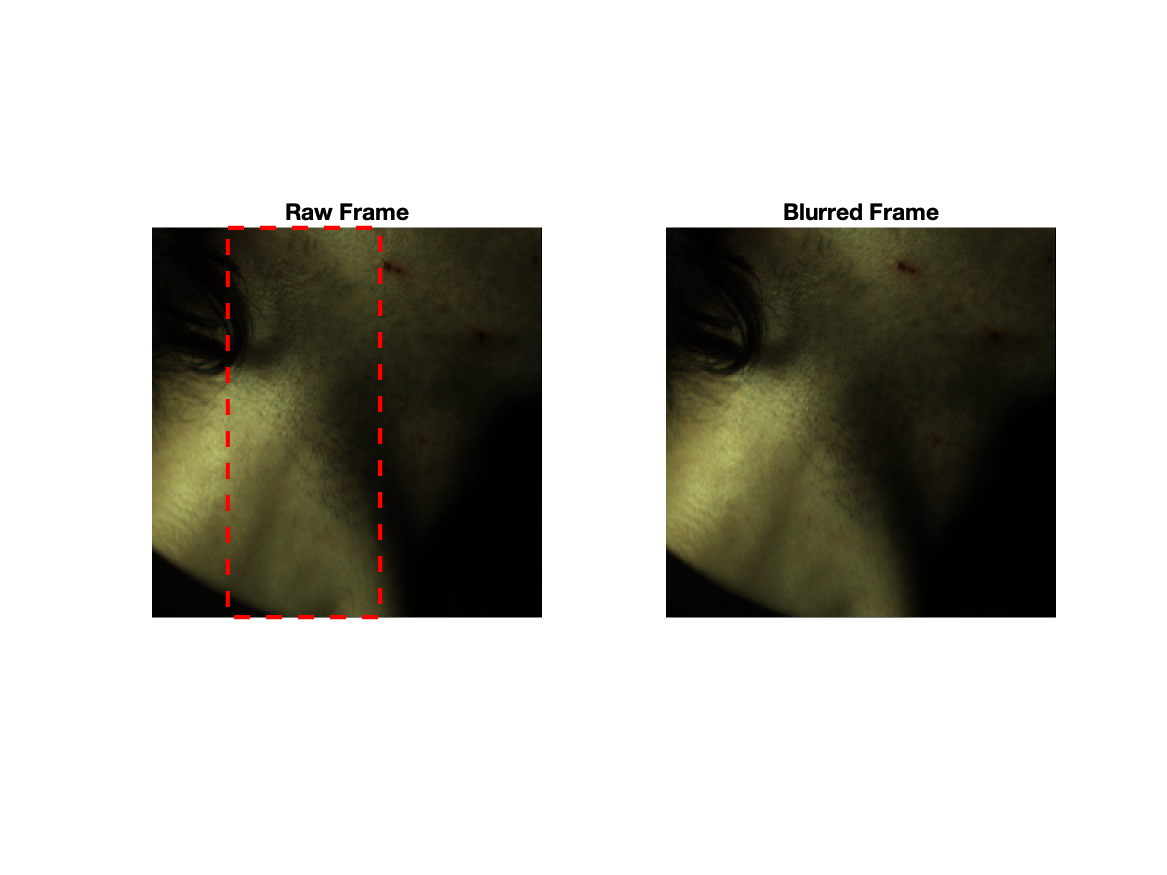 | 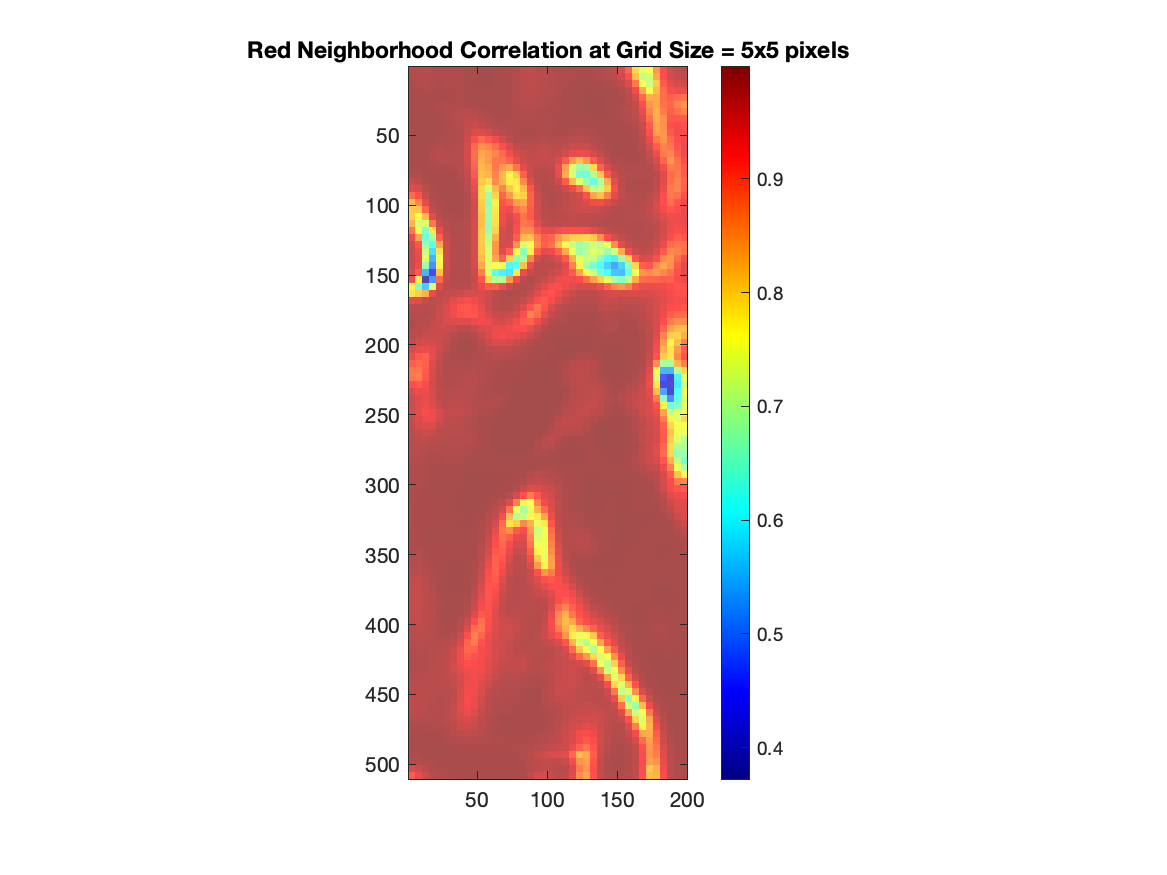 |
| --- | --- |

Figure 19: ROI (left) and local correlation analysis (right)

| 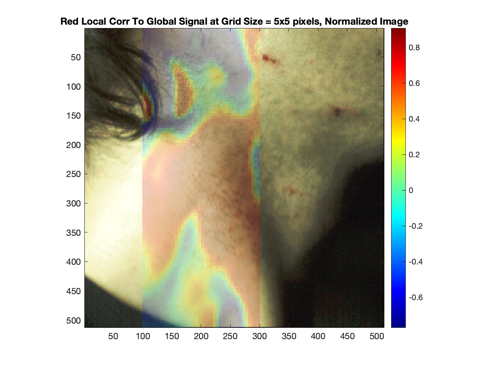 | 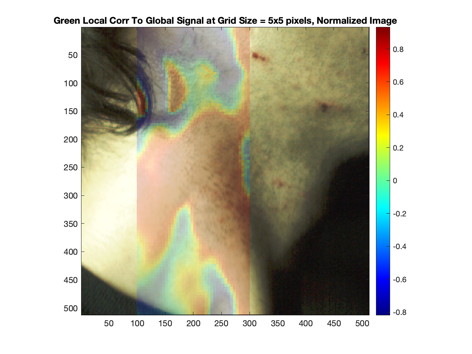 | 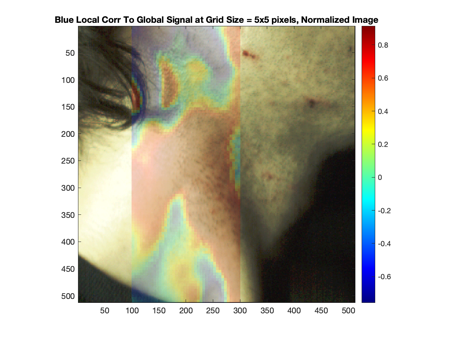 |
| --- | --- | --- |

Figure 20: Correlation of each local SVI (red on left, green in middle, blue on right) to the global SVI signal extracted from the ROI.

| 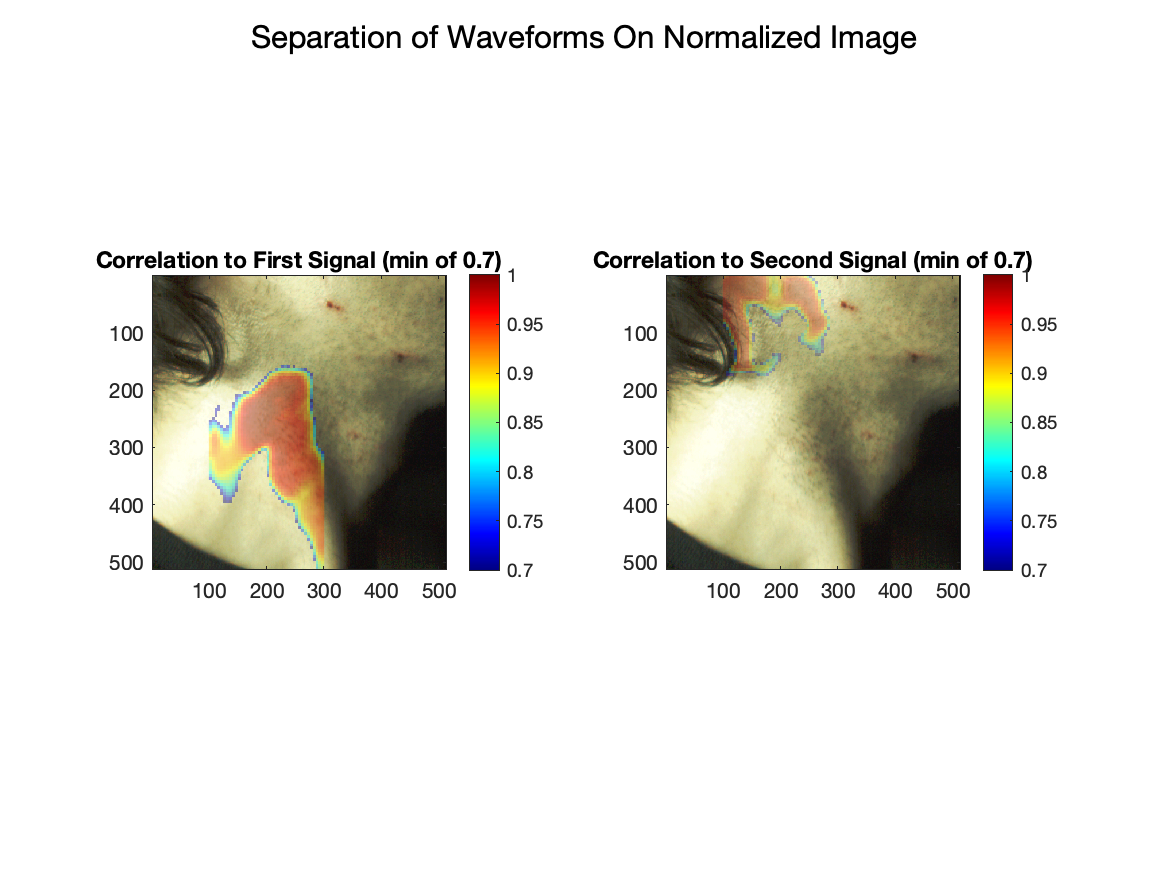 | 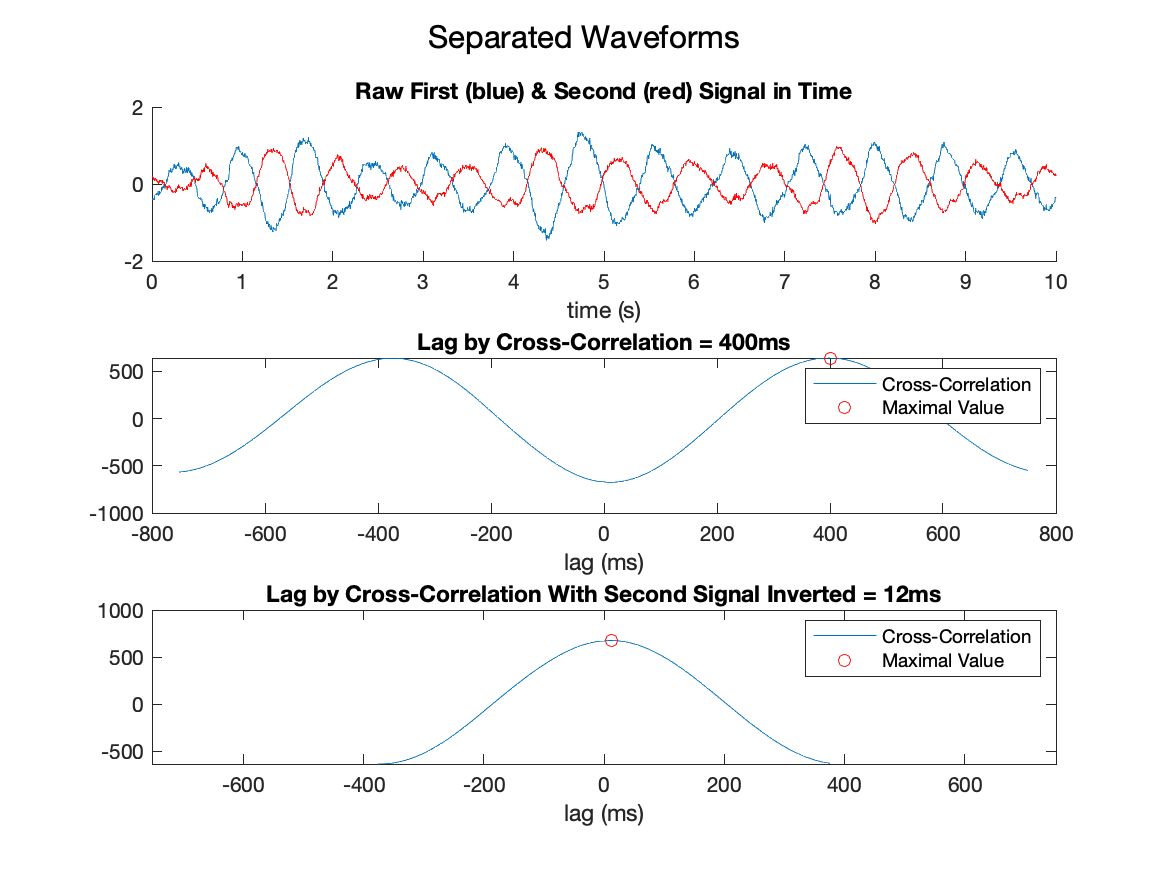 |
| --- | --- |
| 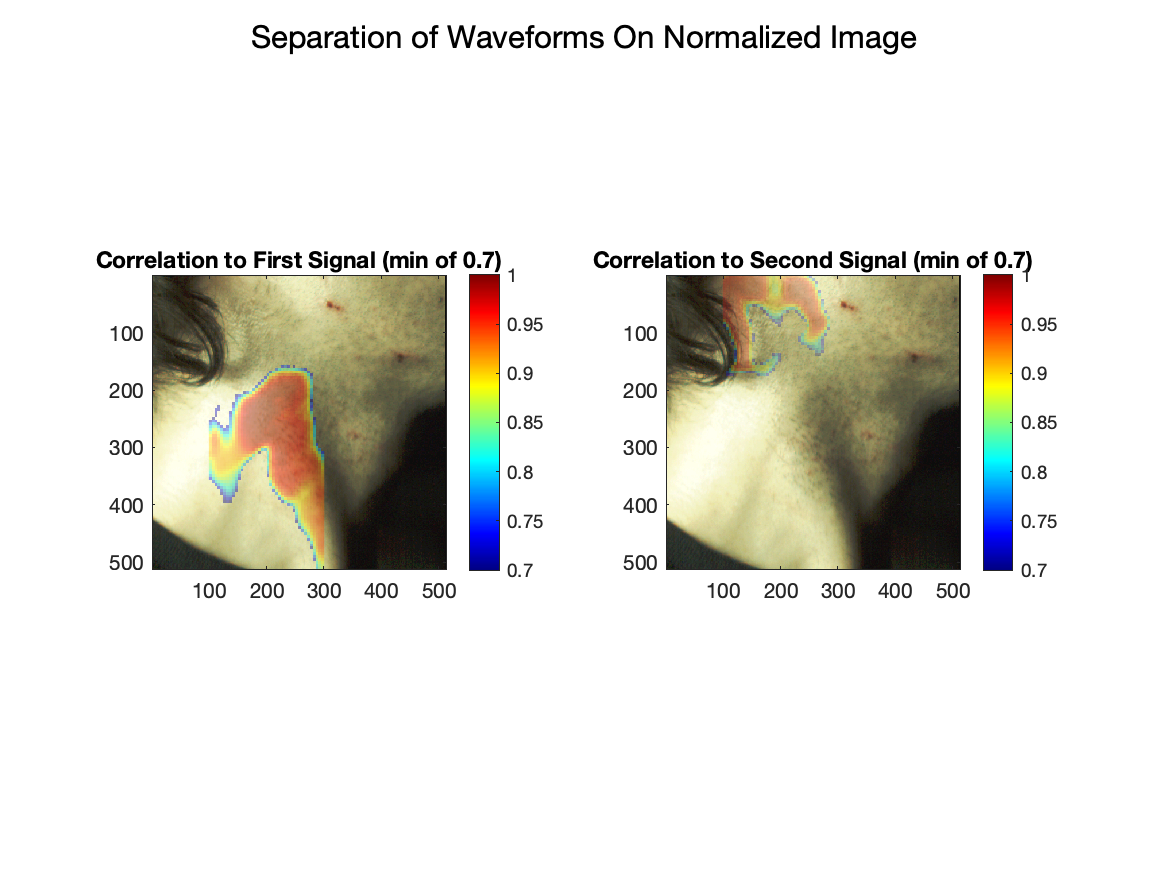 |  |

Figure 21: the first region generating local SVI signals with high internal correlation (top left), the second region generating local SVI signal with high internal correlation (top right), the SVI signals from the first and second regions plotted in the time domain and analyzed with cross-correlation (right).

## Subject 5, Signal A

| 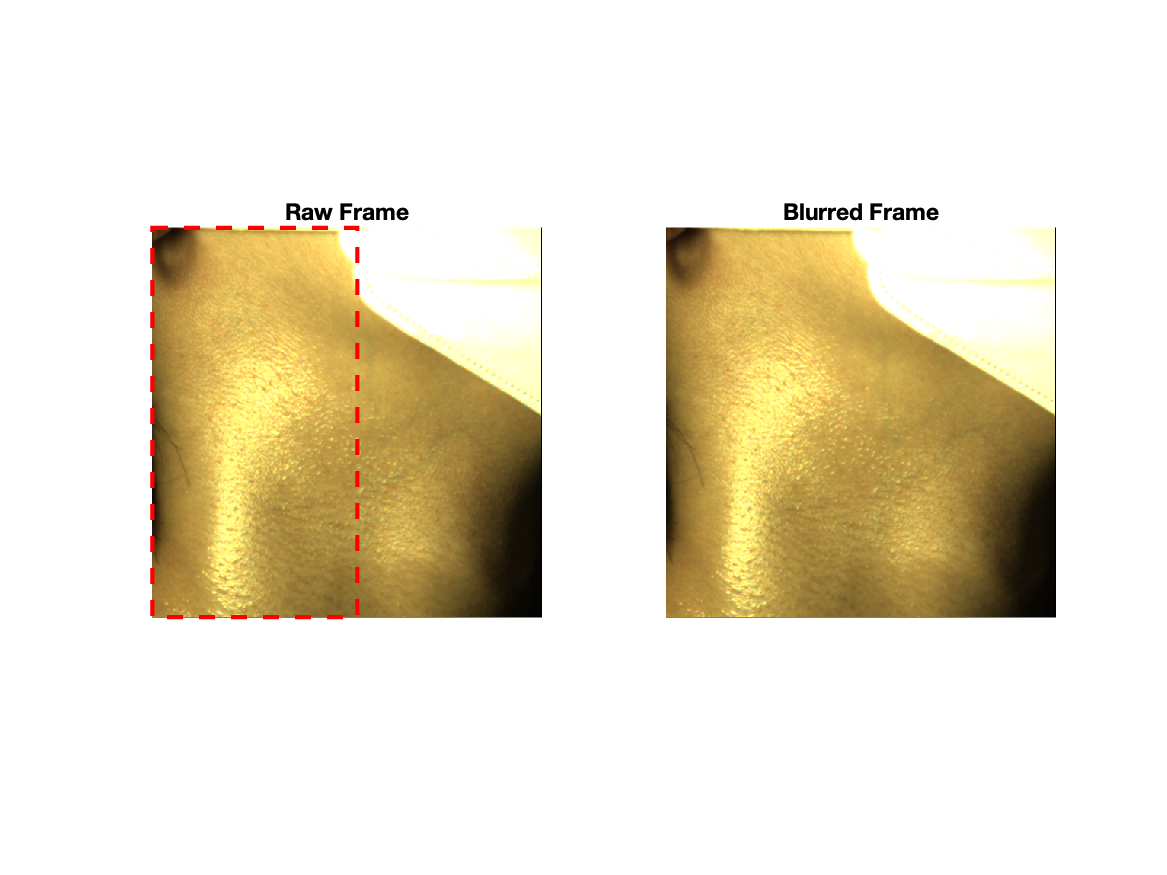 | 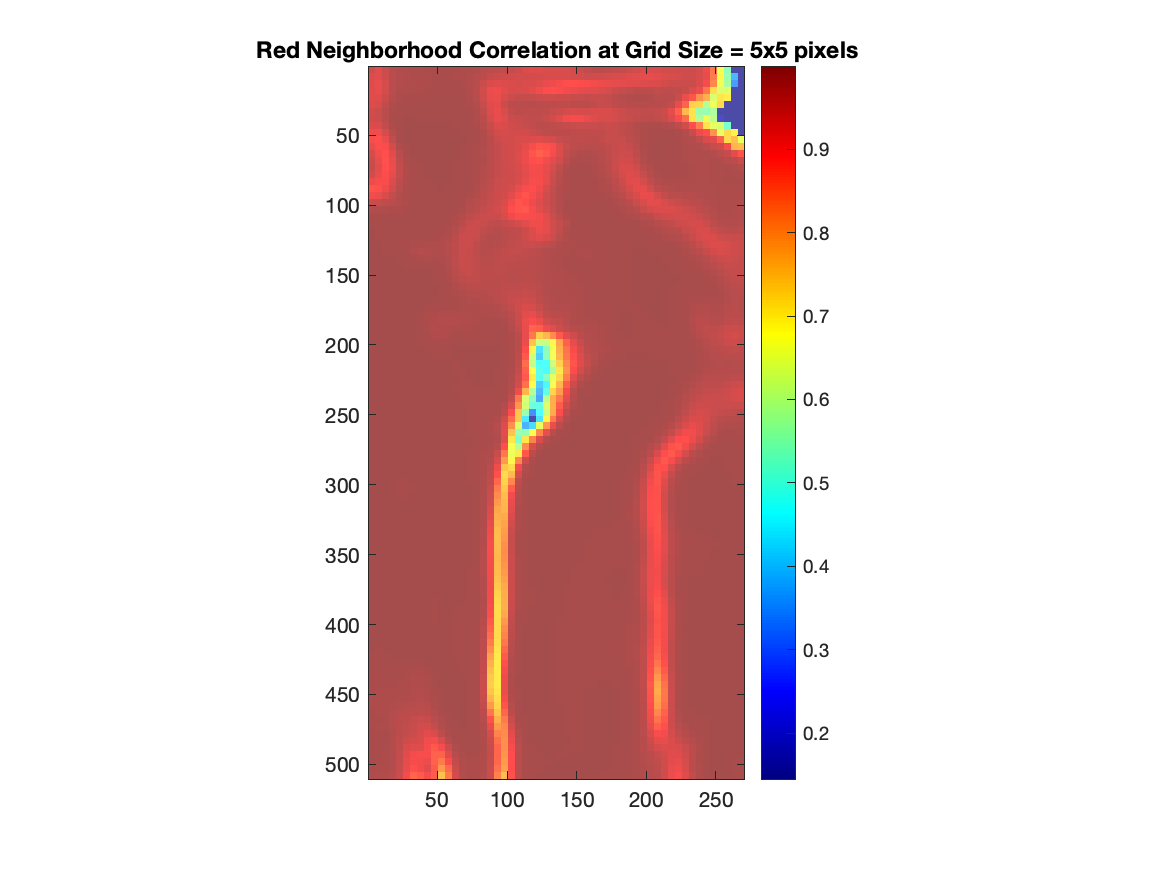 |
| --- | --- |

Figure 22: ROI (left) and local correlation analysis (right)

| 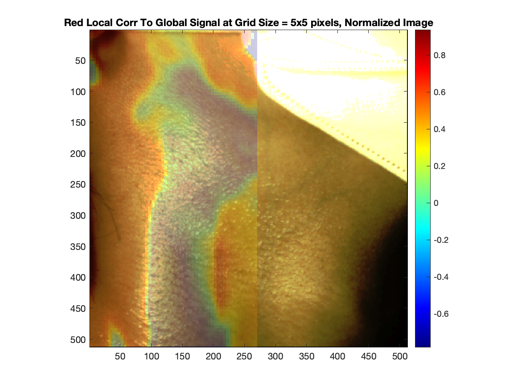 | 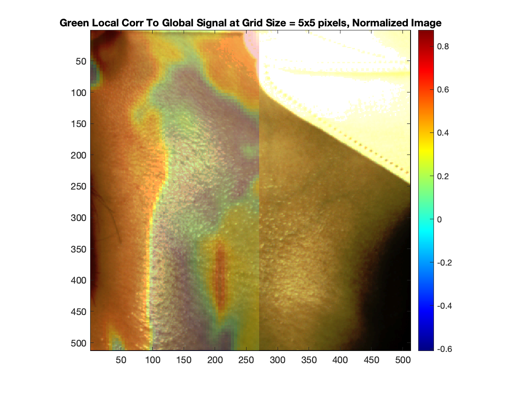 | 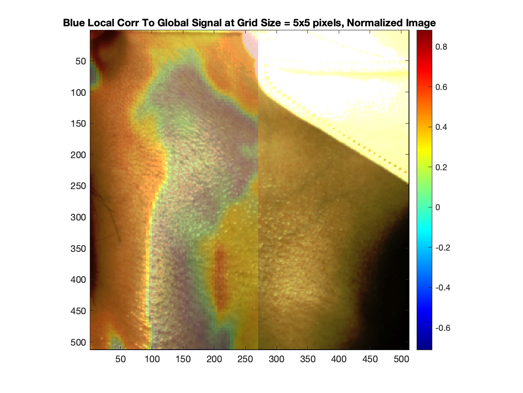 |
| --- | --- | --- |

Figure 23: Correlation of each local SVI (red on left, green in middle, blue on right) to the global SVI signal extracted from the ROI.

| 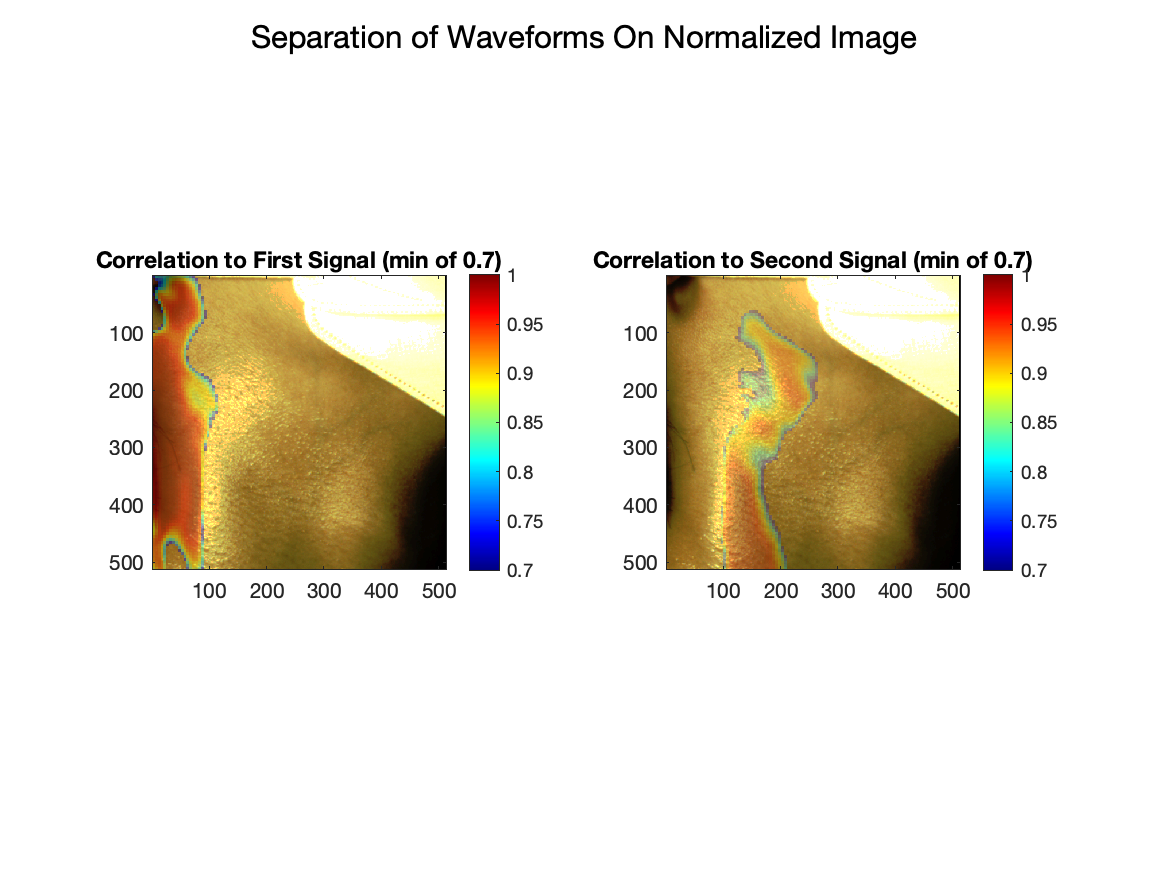 | 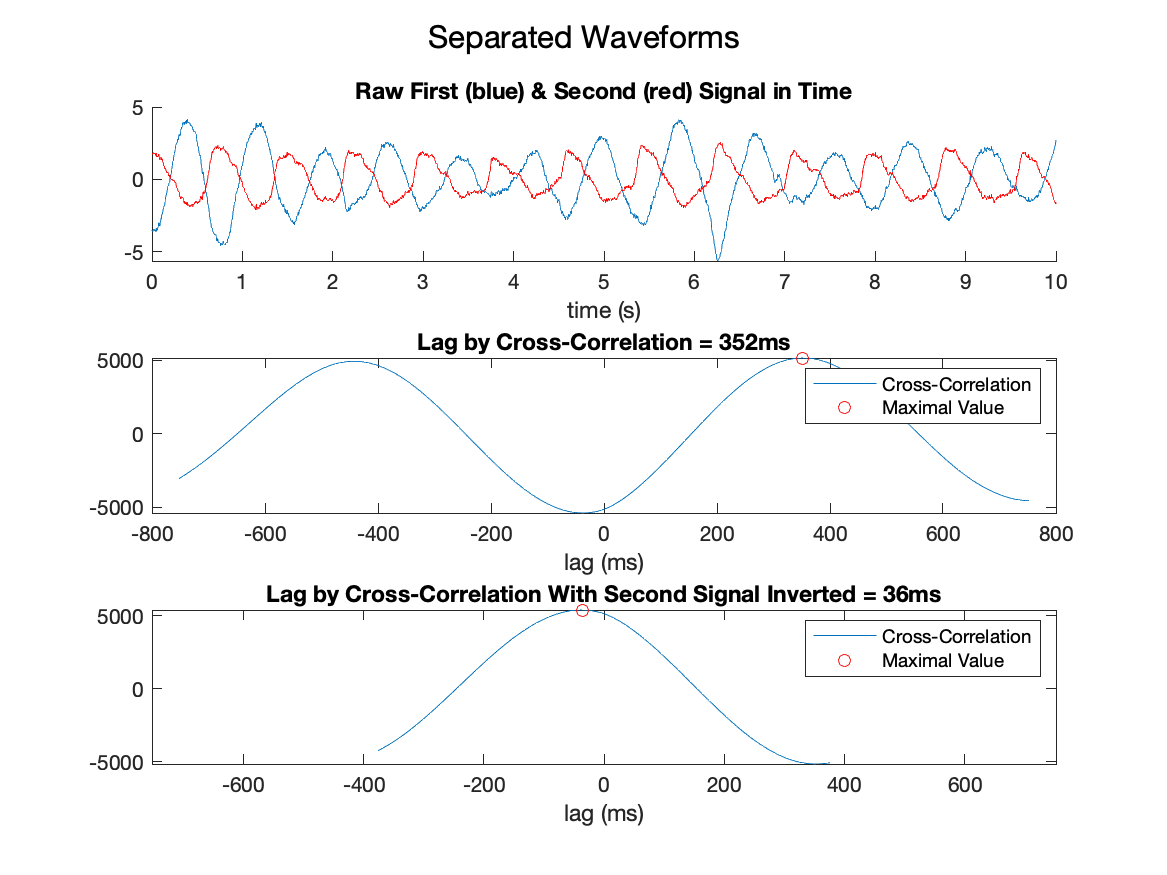 |
| --- | --- |
| 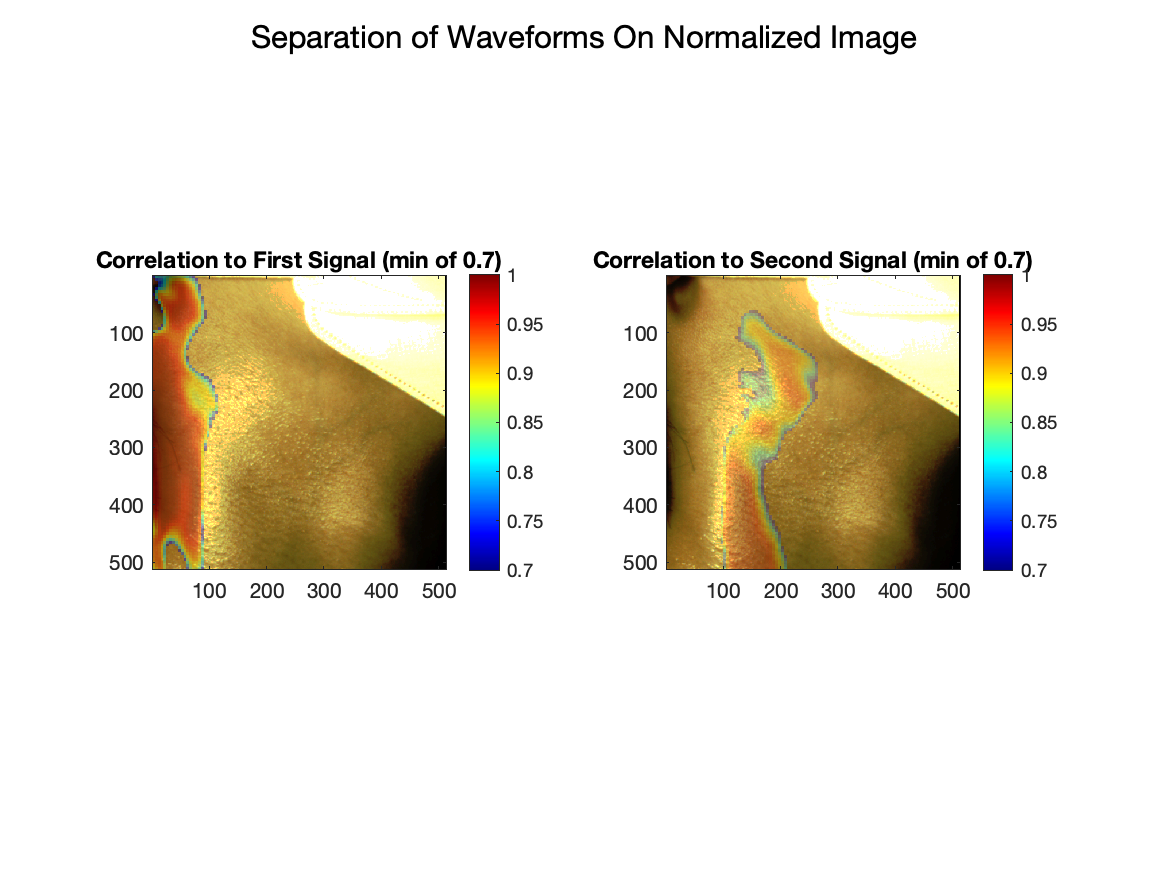 |  |

Figure 24: the first region generating local SVI signals with high internal correlation (top left), the second region generating local SVI signal with high internal correlation (top right), the SVI signals from the first and second regions plotted in the time domain and analyzed with cross-correlation (right).

## Subject 5, Signal B

| 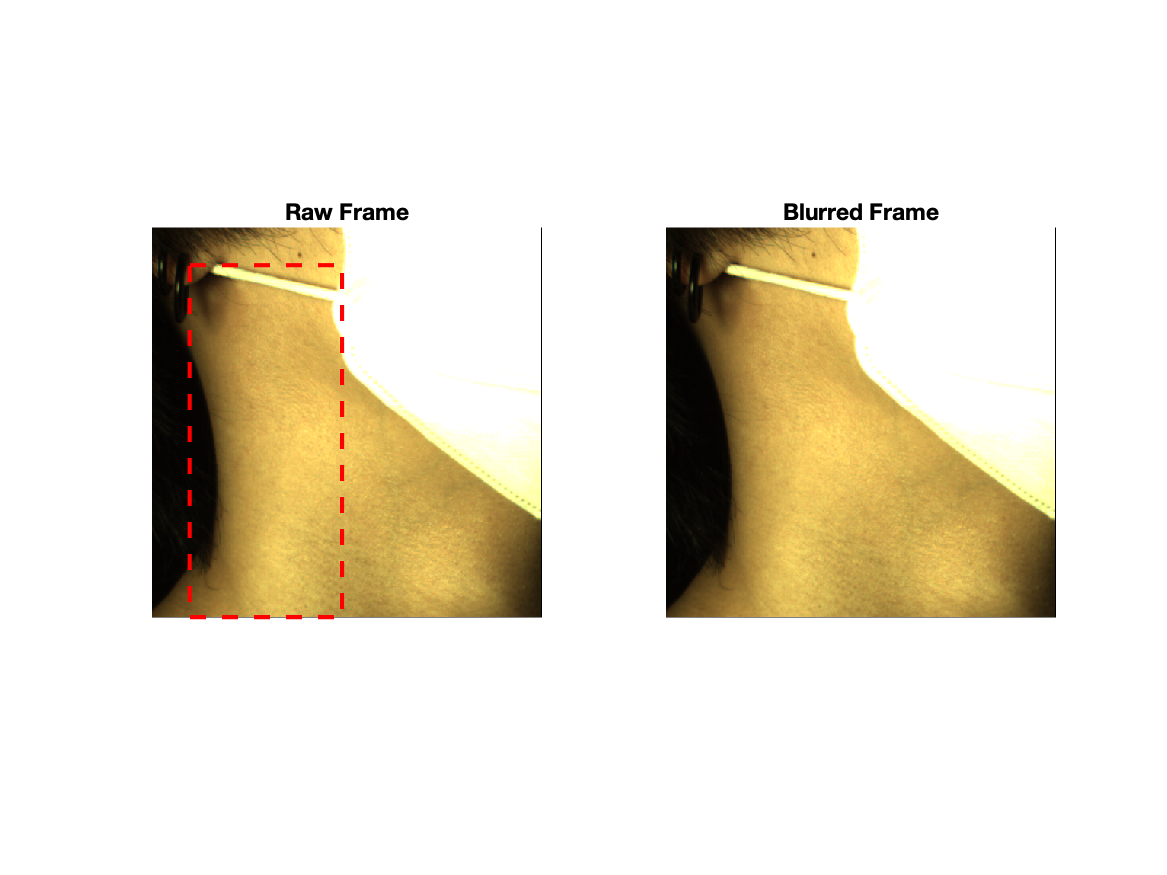 | 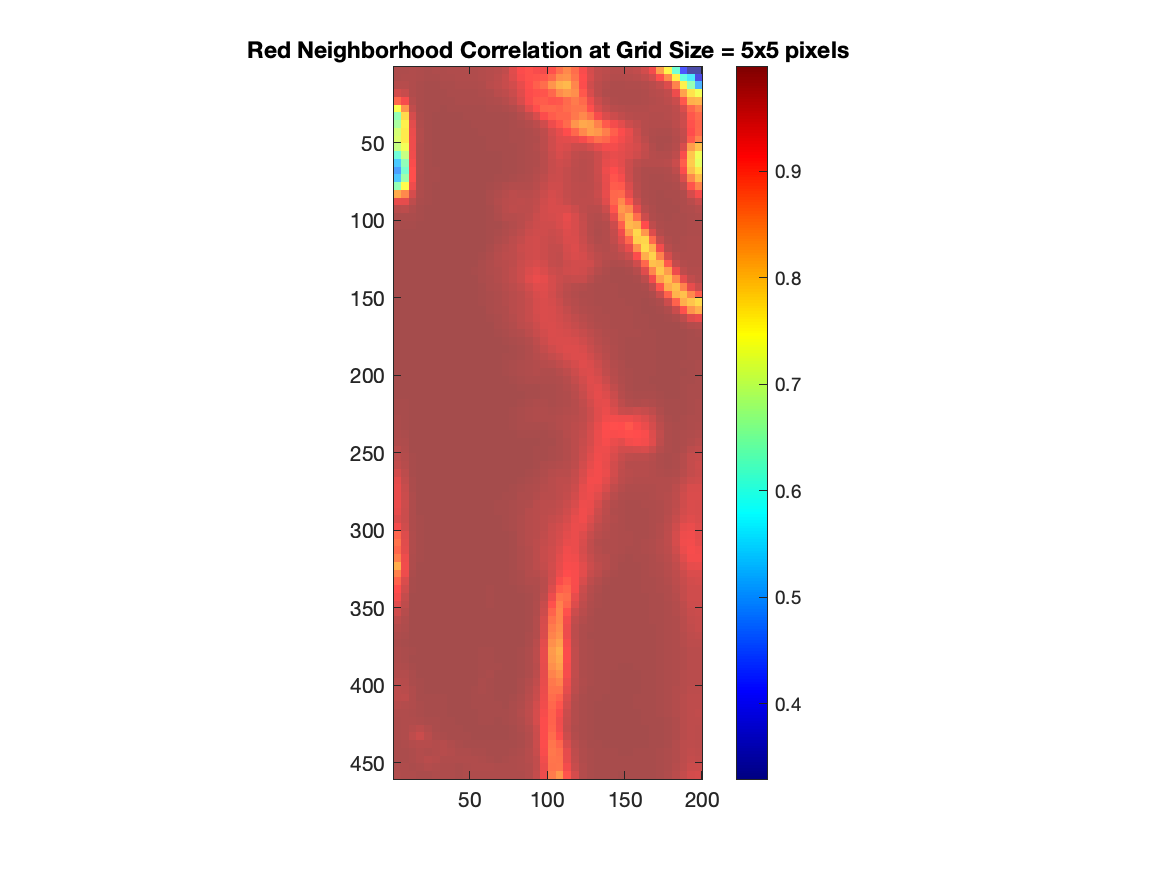 |
| --- | --- |

Figure 25: ROI (left) and local correlation analysis (right)

| 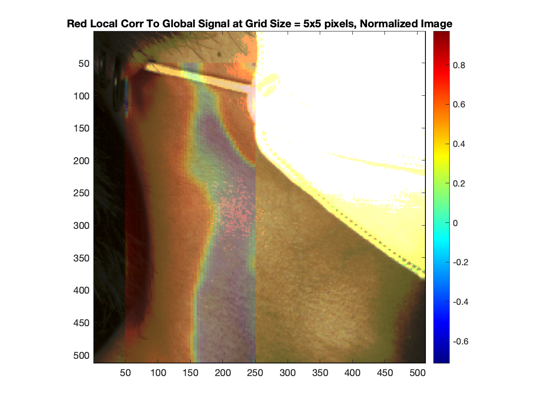 | 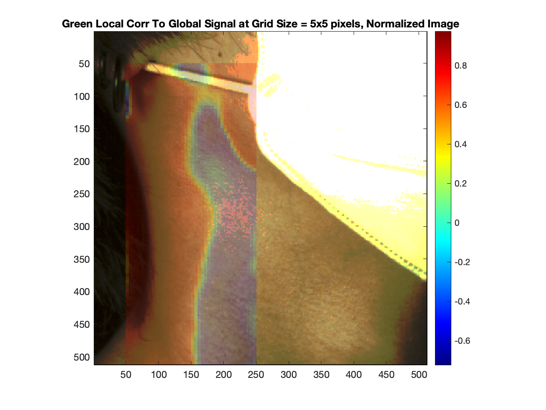 | 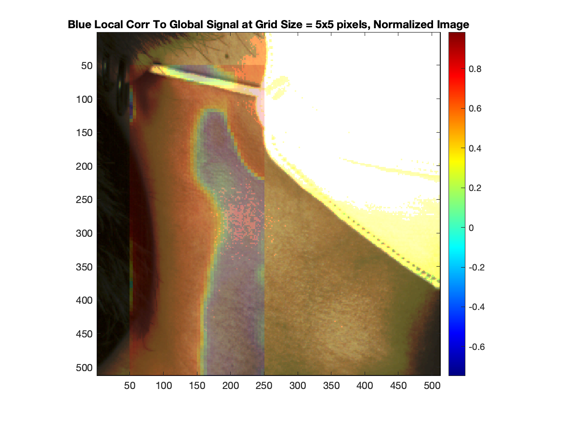 |
| --- | --- | --- |

Figure 26: Correlation of each local SVI (red on left, green in middle, blue on right) to the global SVI signal extracted from the ROI.

| 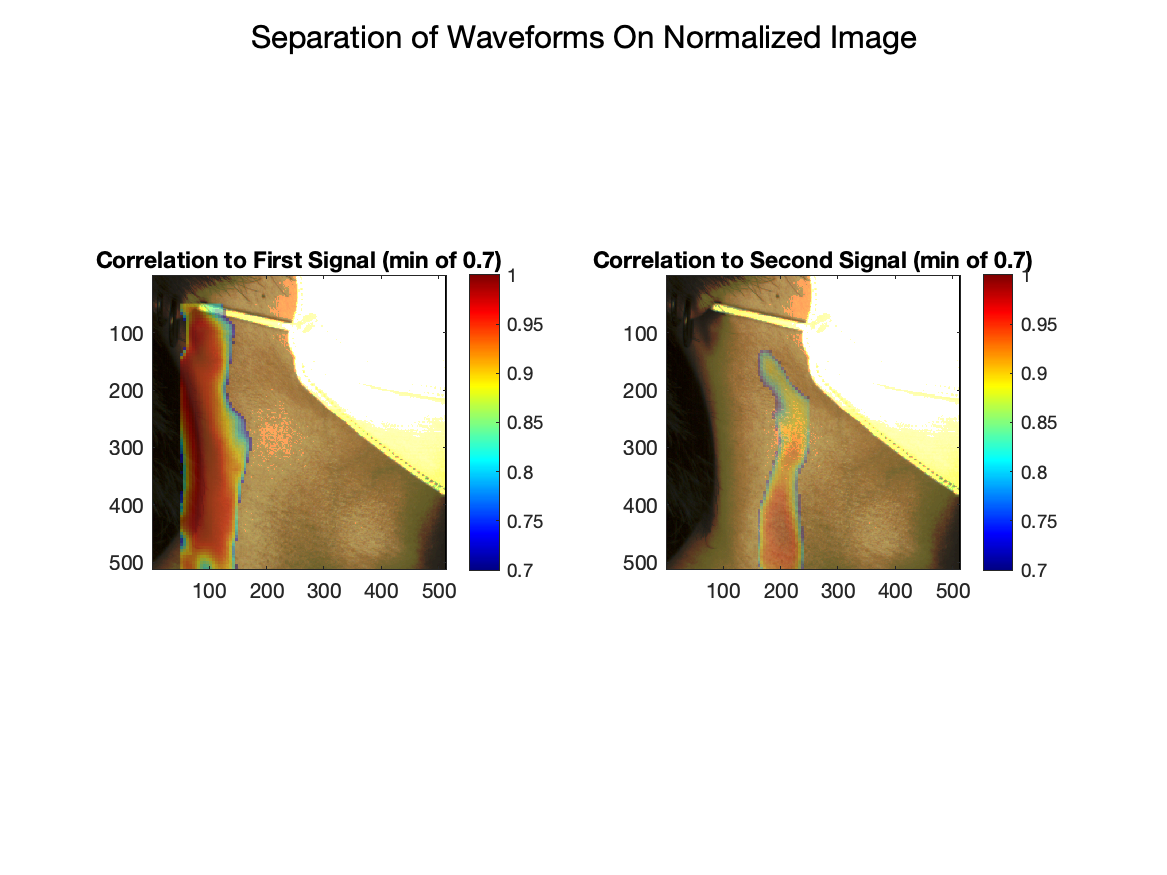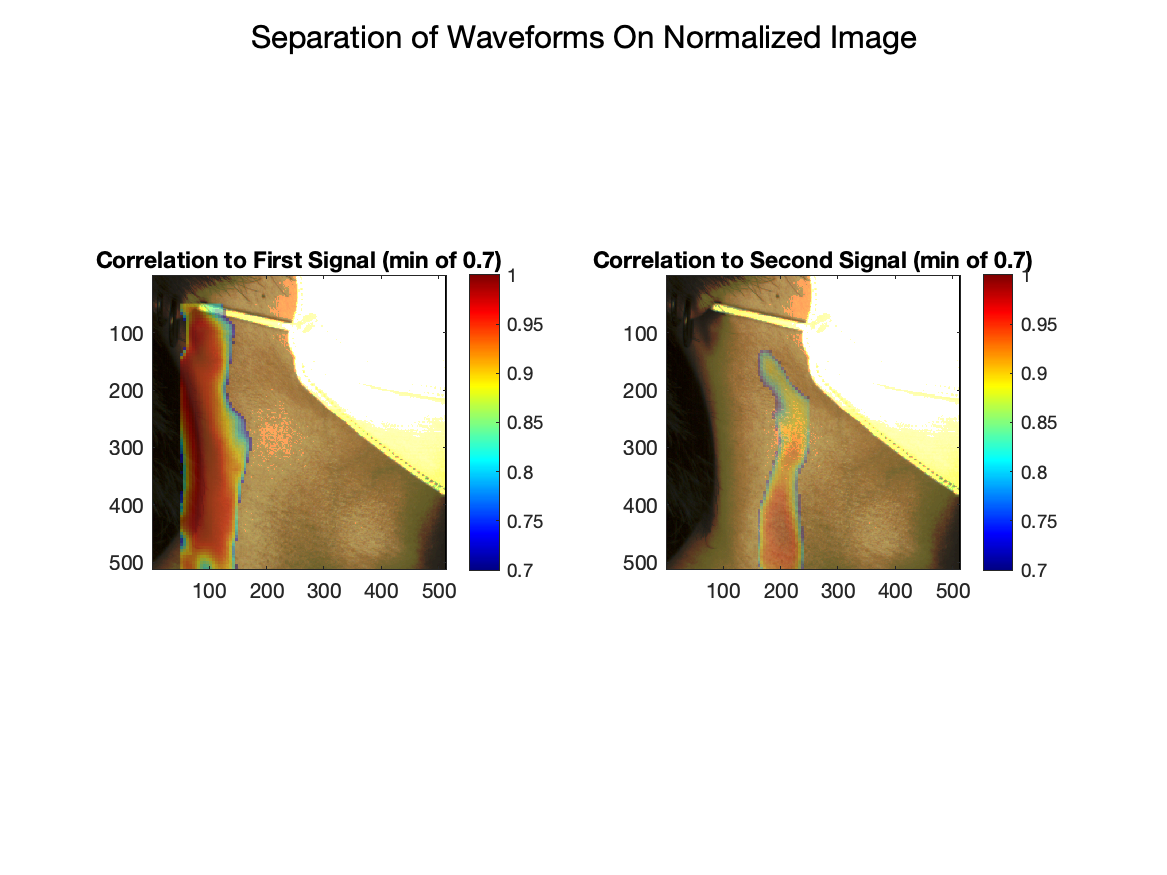 | 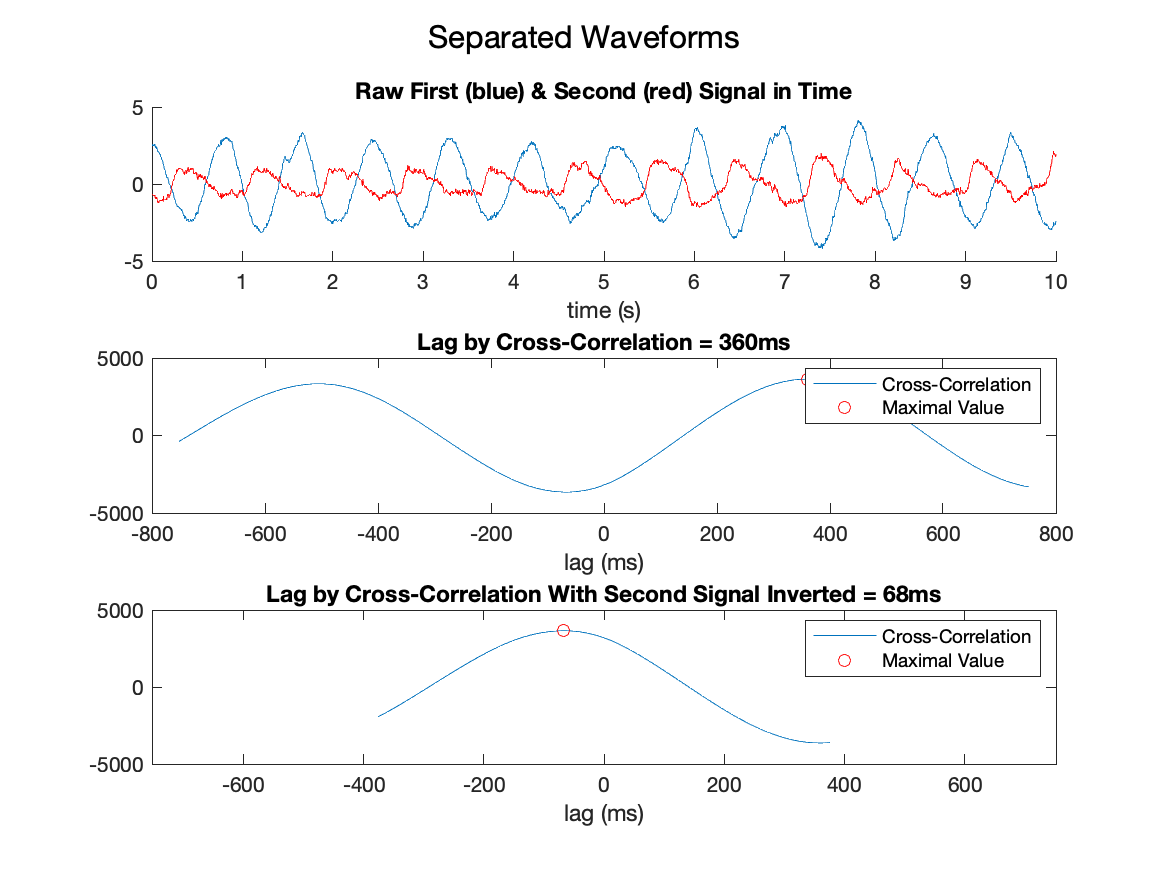 |
| --- | --- |
|  |  |

Figure 27: the first region generating local SVI signals with high internal correlation (top left), the second region generating local SVI signal with high internal correlation (top right), the SVI signals from the first and second regions plotted in the time domain and analyzed with cross-correlation (right).

## Subject 6, Signal A

| 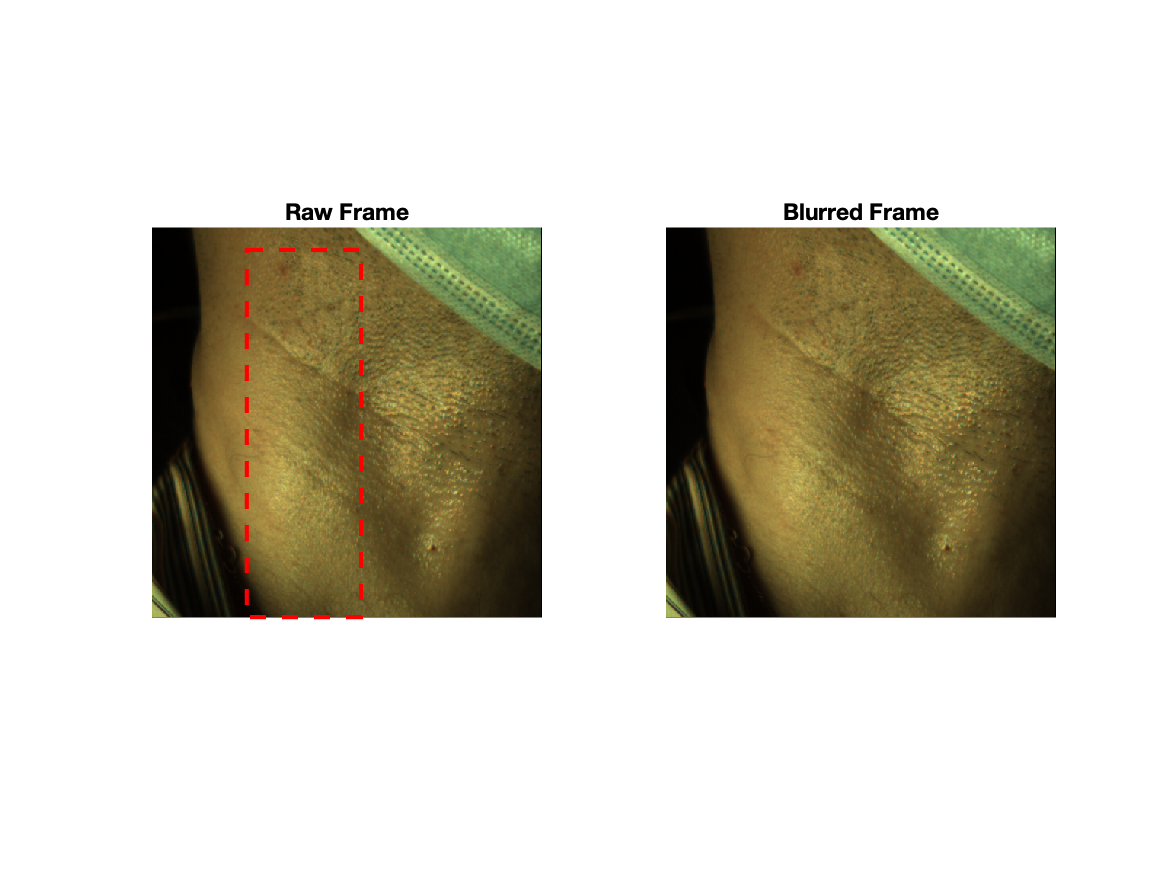 | 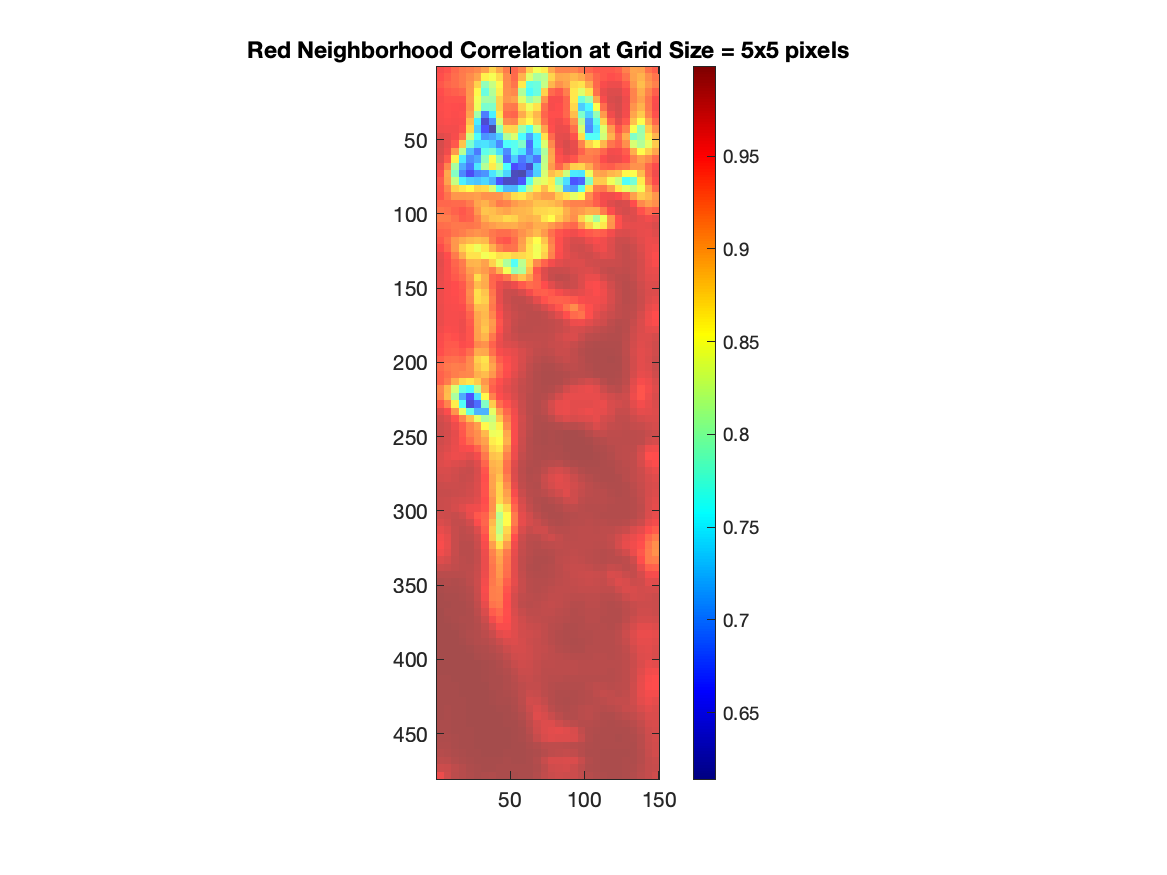 |
| --- | --- |

Figure 28: ROI (left) and local correlation analysis (right)

| 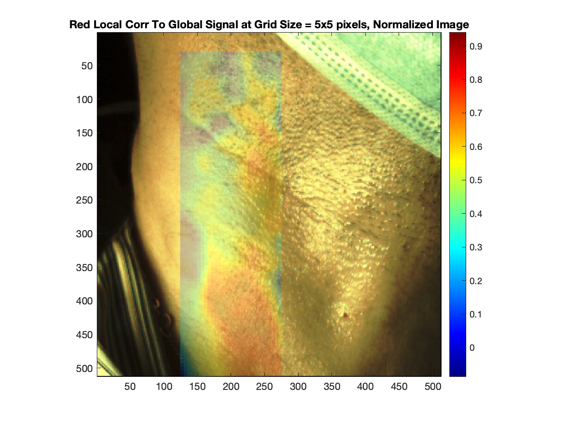 | 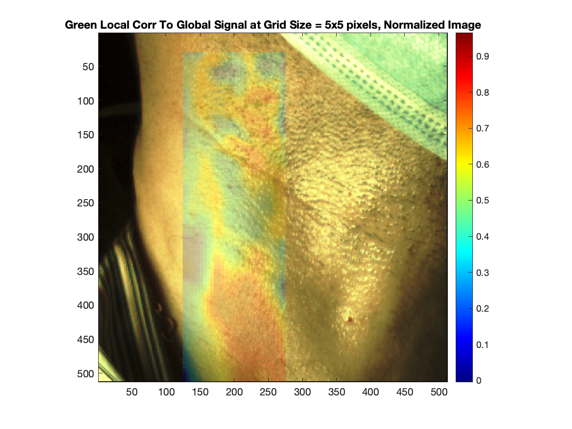 | 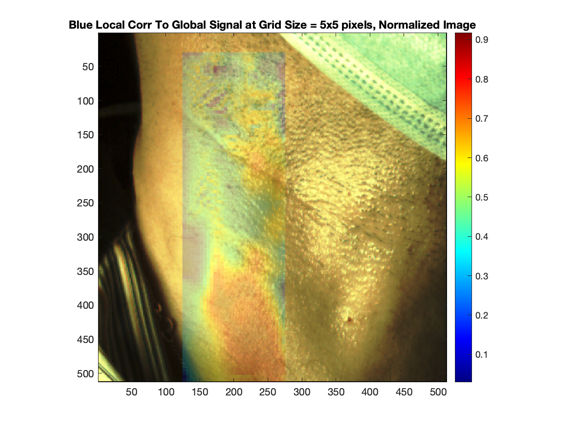 |
| --- | --- | --- |

Figure 29: Correlation of each local SVI (red on left, green in middle, blue on right) to the global SVI signal extracted from the ROI.

| 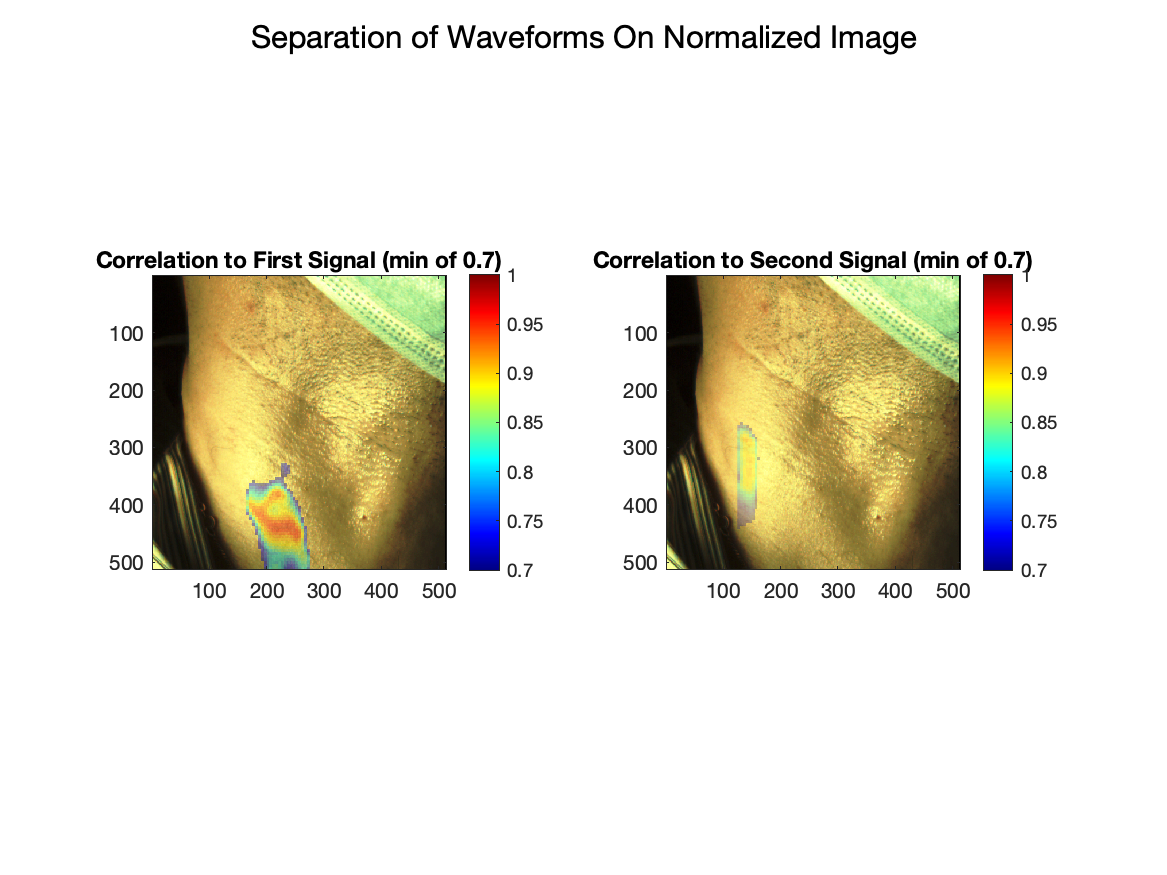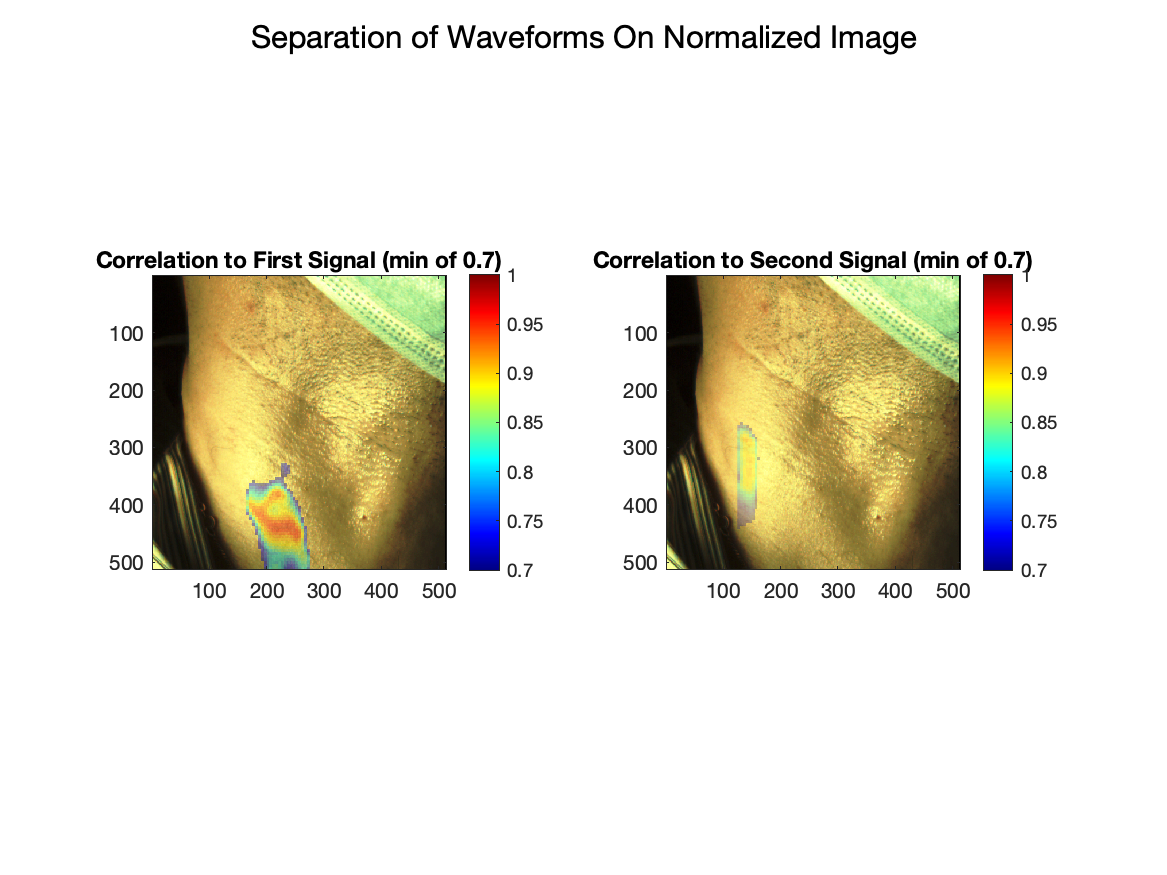 | 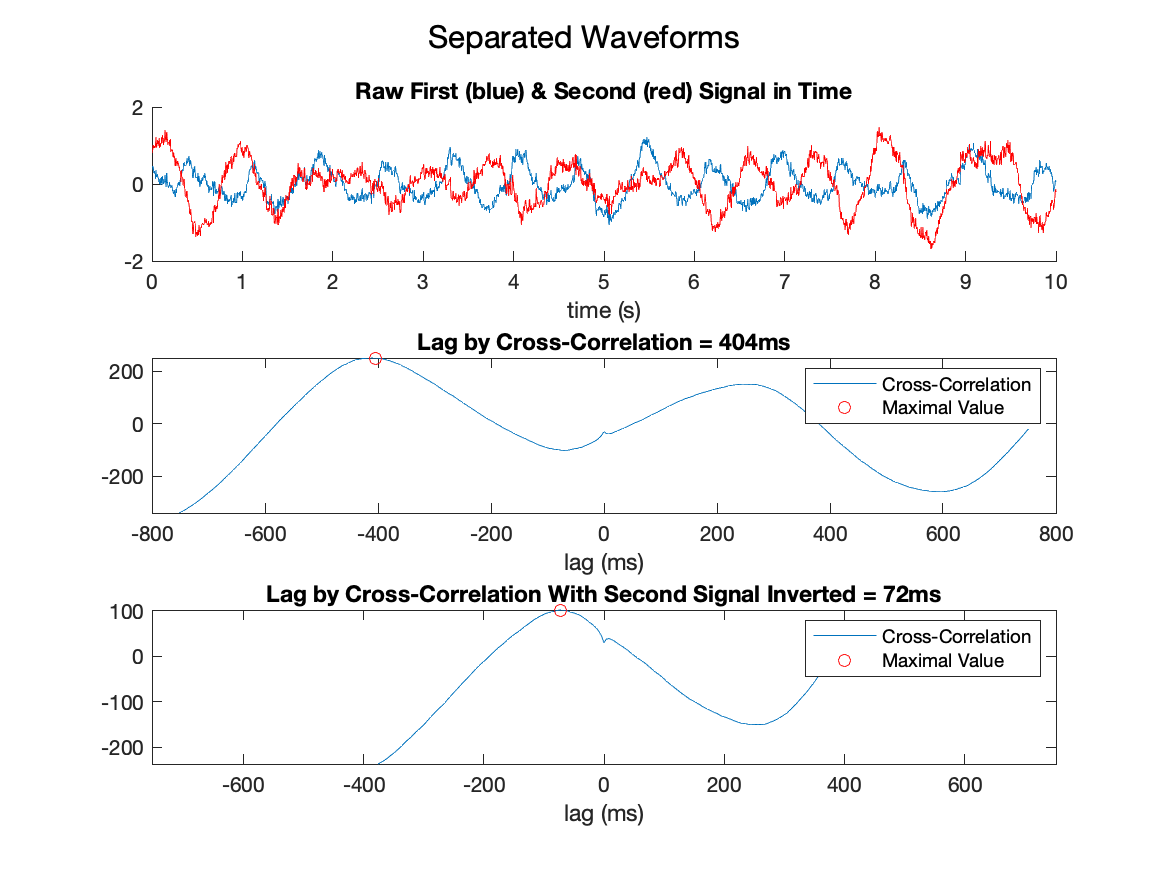 |
| --- | --- |
|  |  |

Figure 30: the first region generating local SVI signals with high internal correlation (top left), the second region generating local SVI signal with high internal correlation (top right), the SVI signals from the first and second regions plotted in the time domain and analyzed with cross-correlation (right).

## Subject 6, Signal B

| 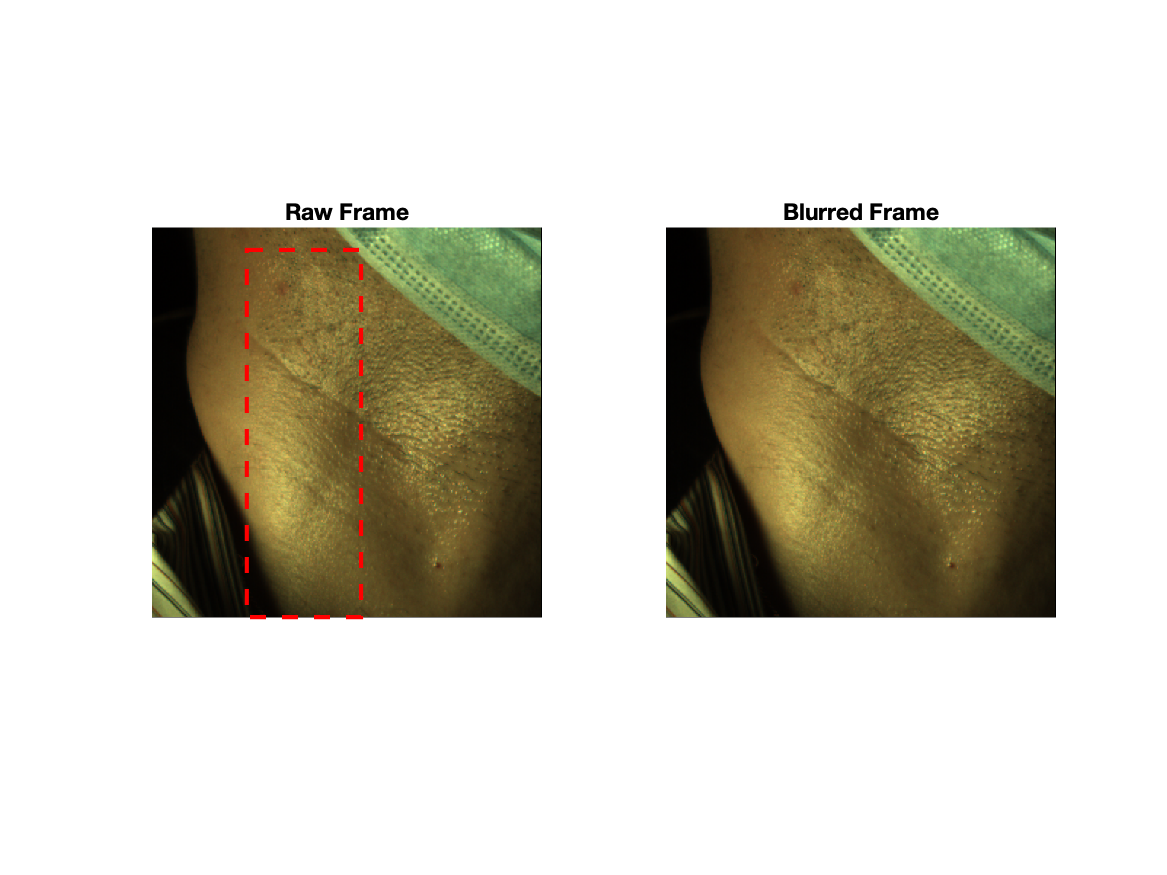 | 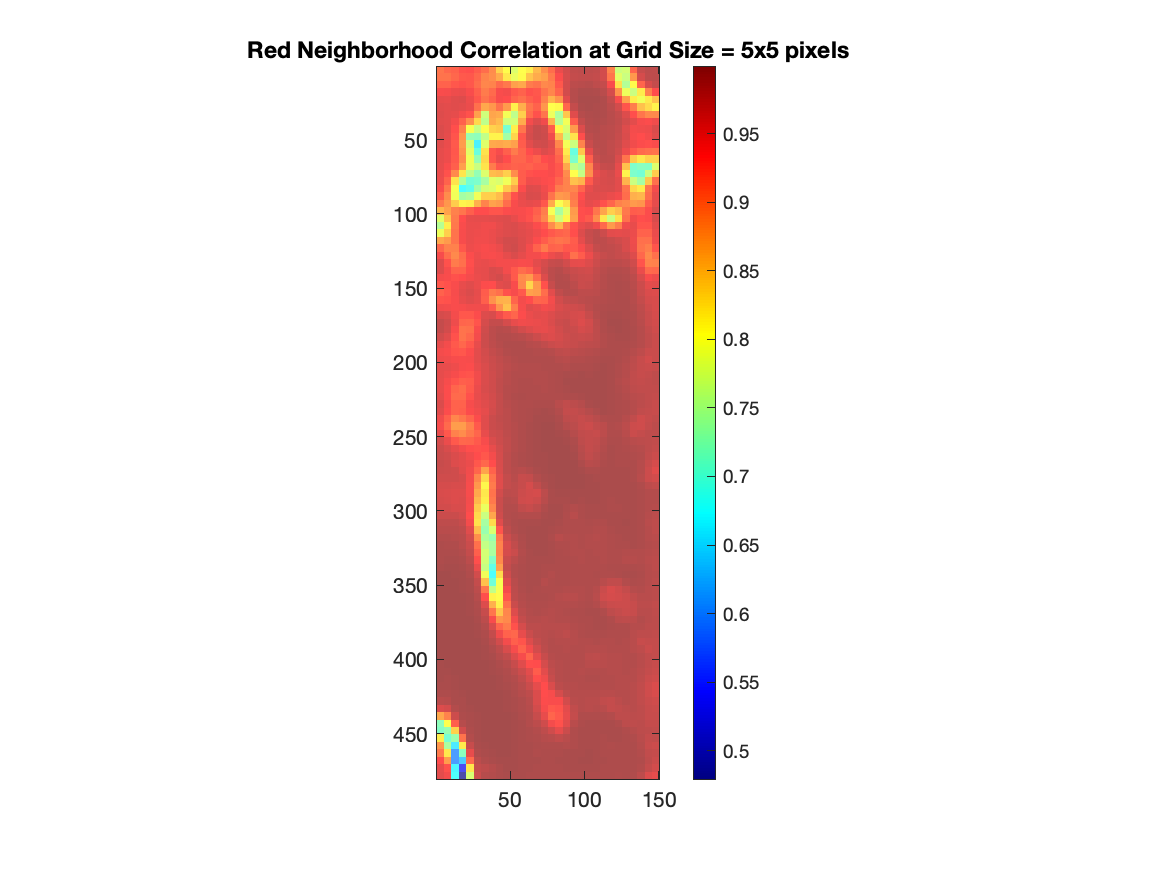 |
| --- | --- |

Figure 31: ROI (left) and local correlation analysis (right)

| 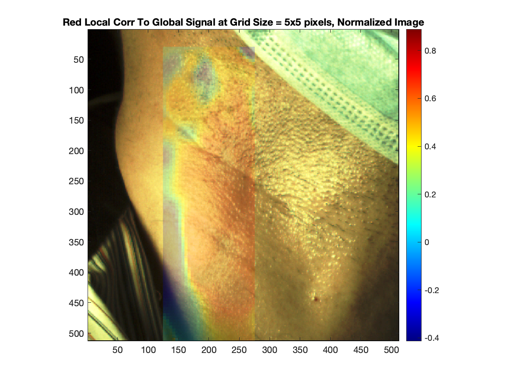 | 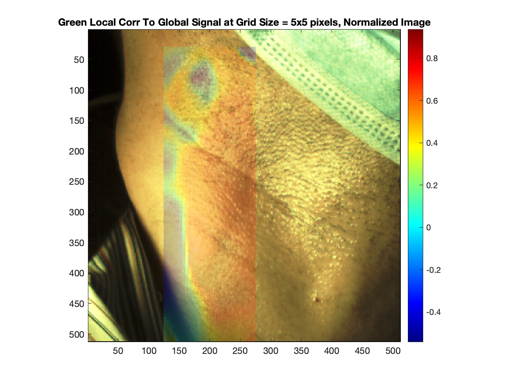 | 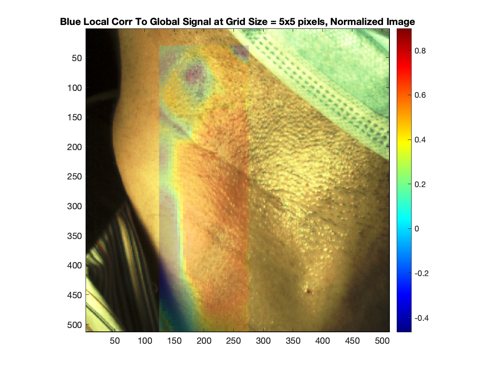 |
| --- | --- | --- |

Figure 32: Correlation of each local SVI (red on left, green in middle, blue on right) to the global SVI signal extracted from the ROI.

| 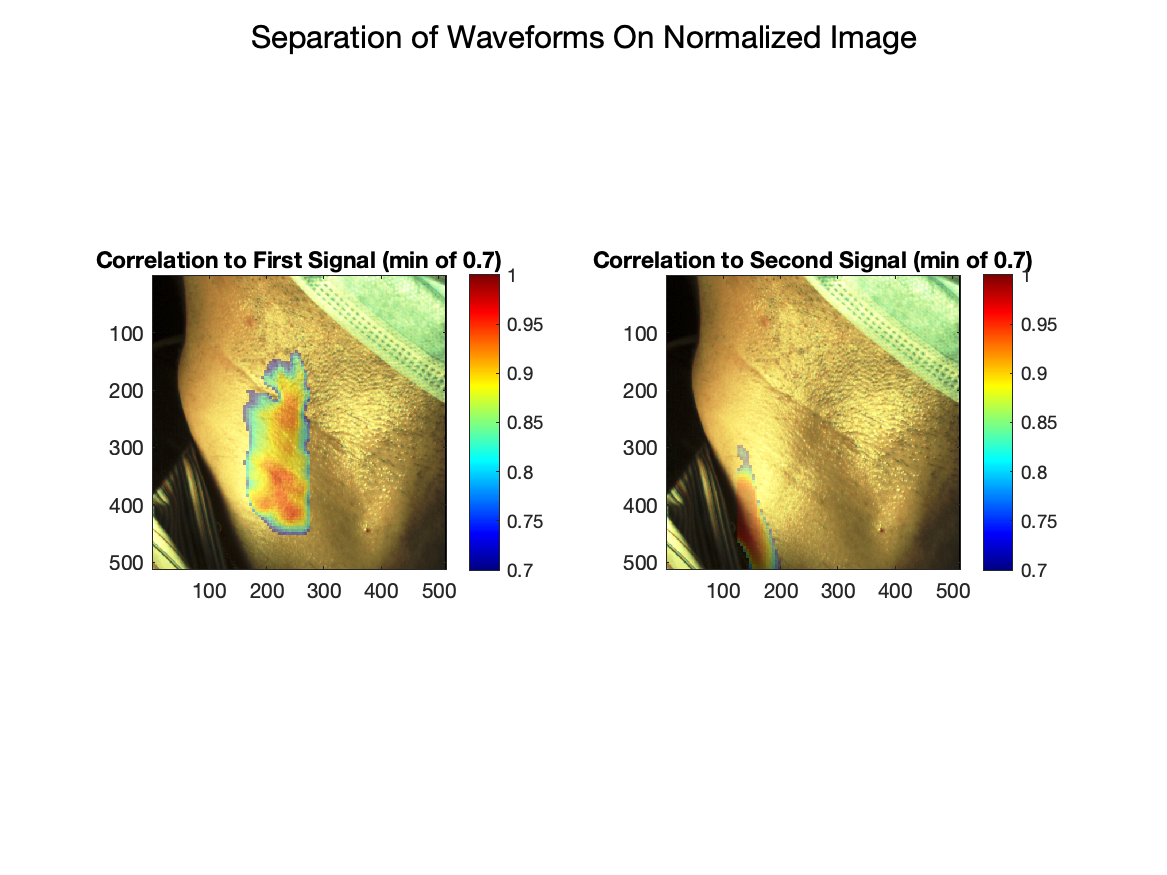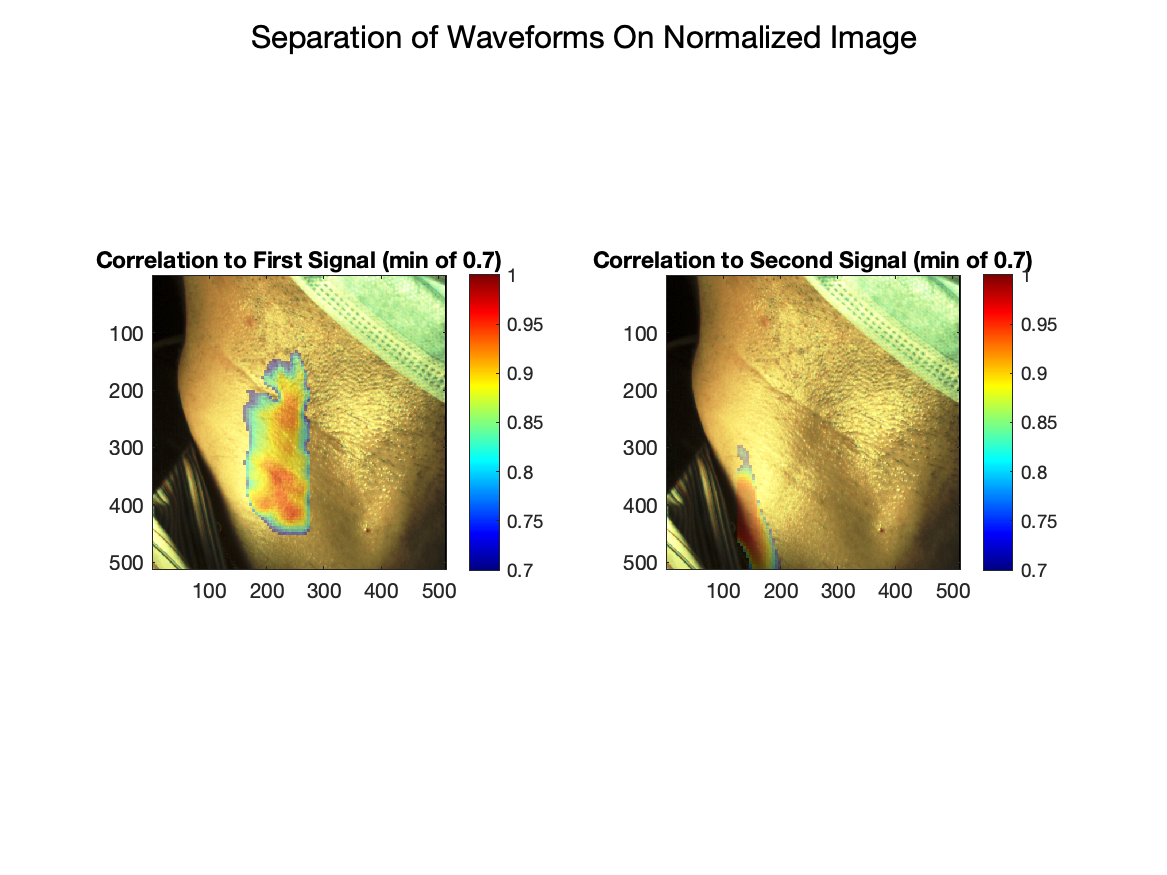 | 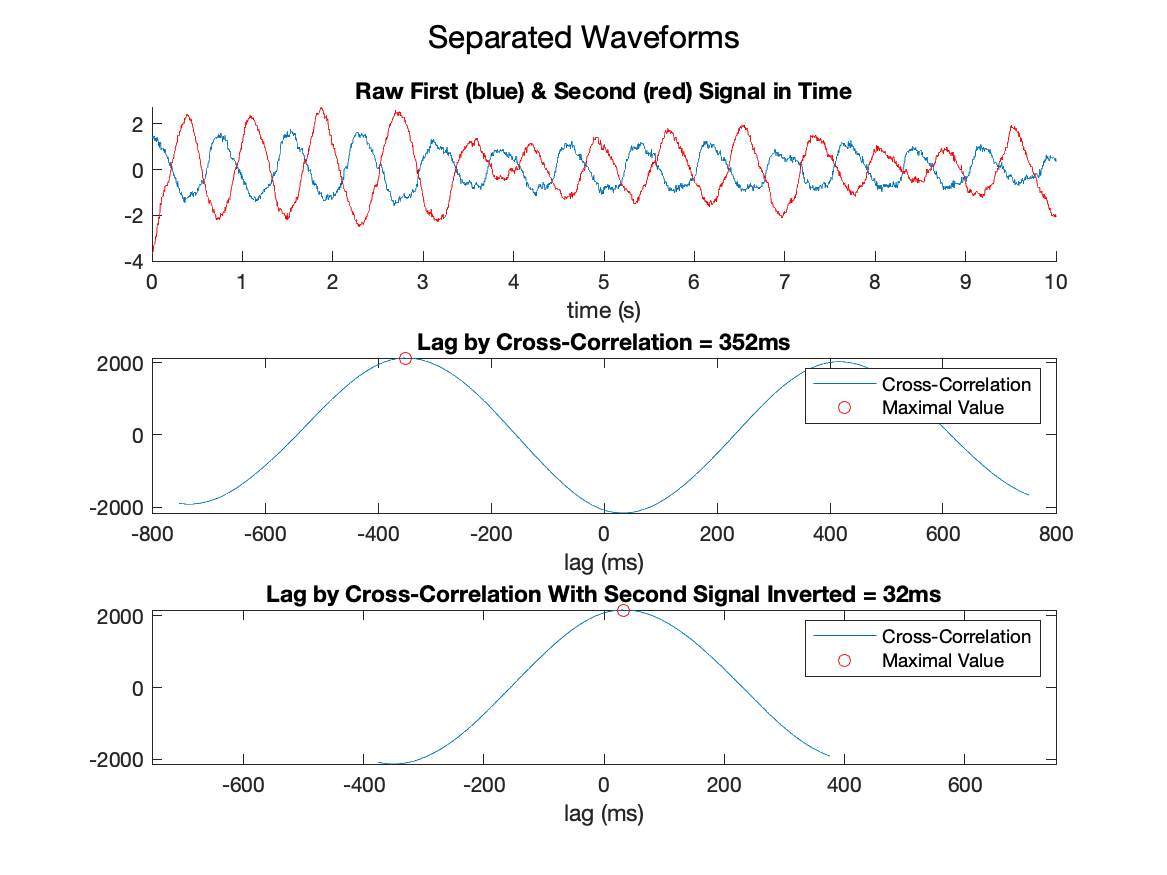 |
| --- | --- |
|  |  |

Figure 33: the first region generating local SVI signals with high internal correlation (top left), the second region generating local SVI signal with high internal correlation (top right), the SVI signals from the first and second regions plotted in the time domain and analyzed with cross-correlation (right).

## Subject 7, Signal A

| 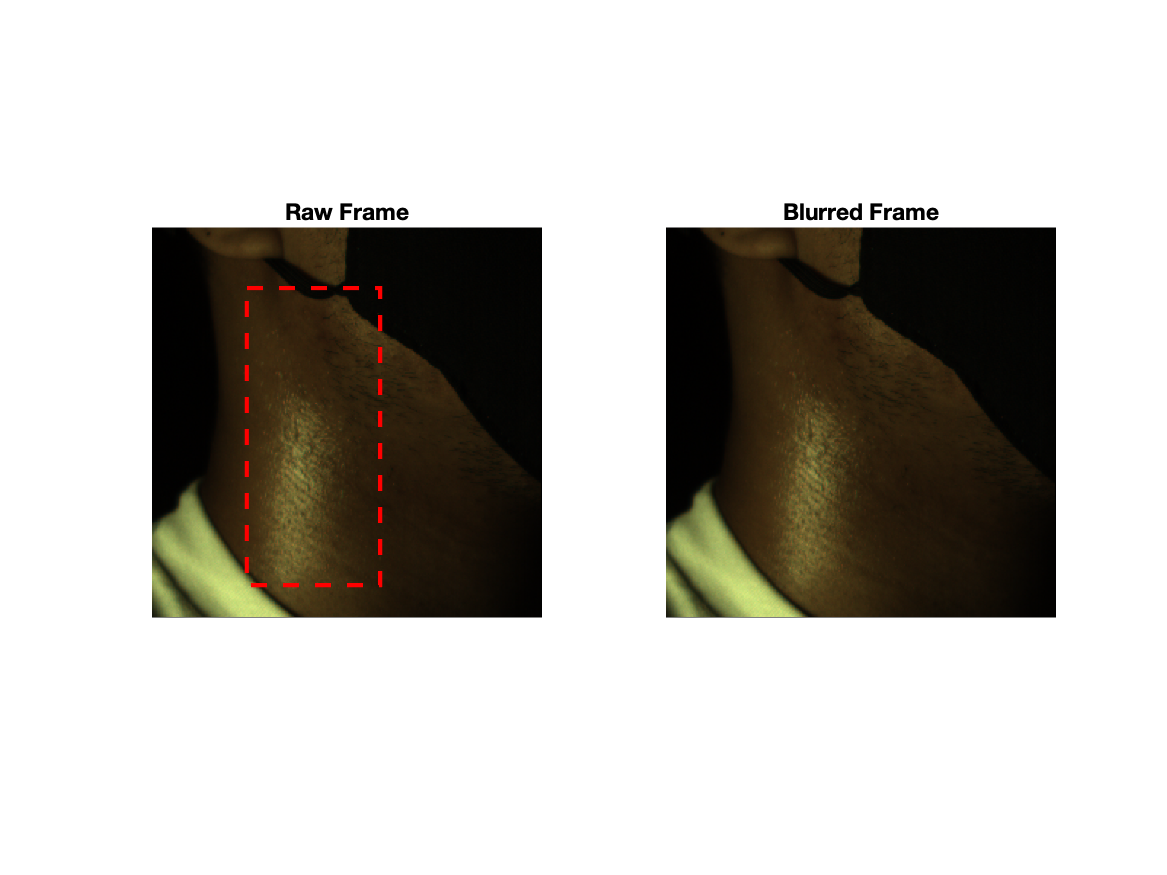 | 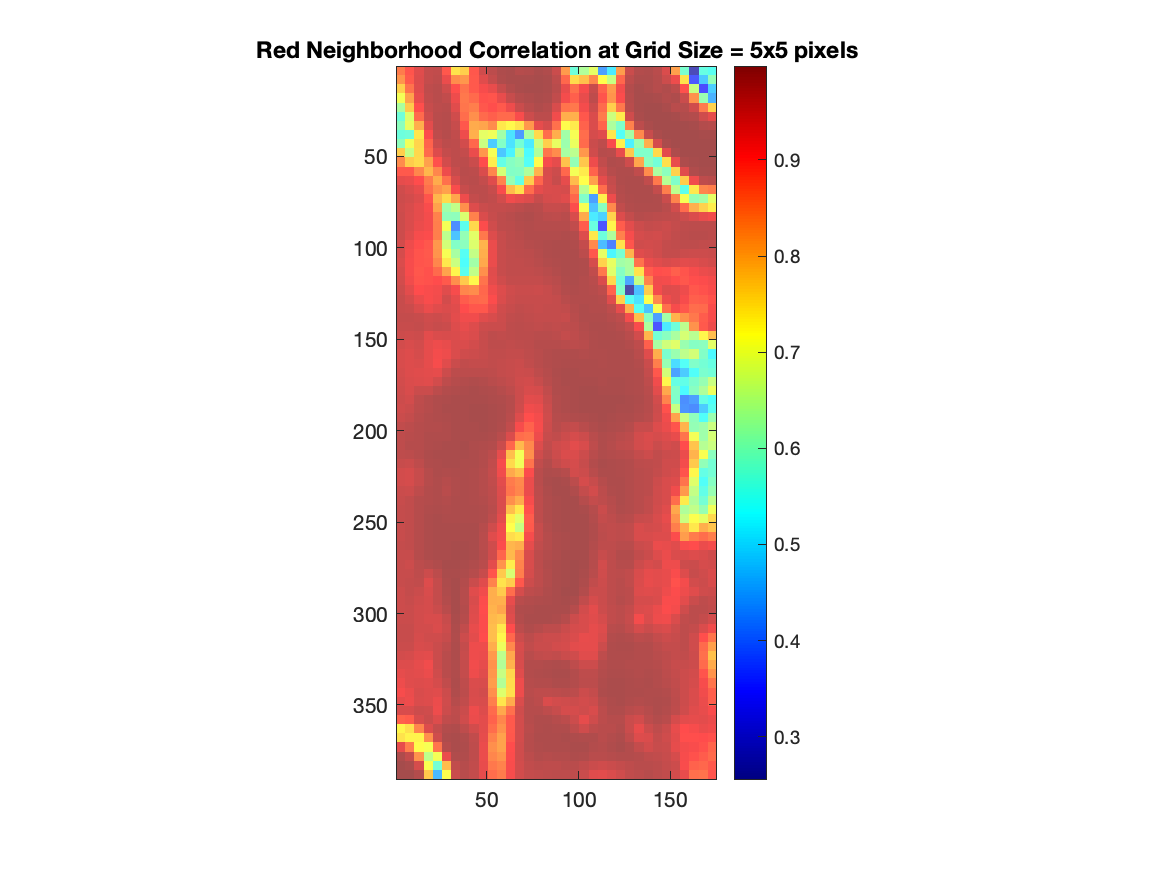 |
| --- | --- |

Figure 34: ROI (left) and local correlation analysis (right)

| 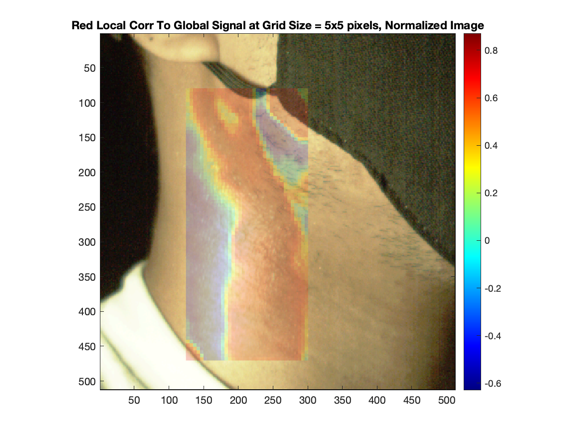 | 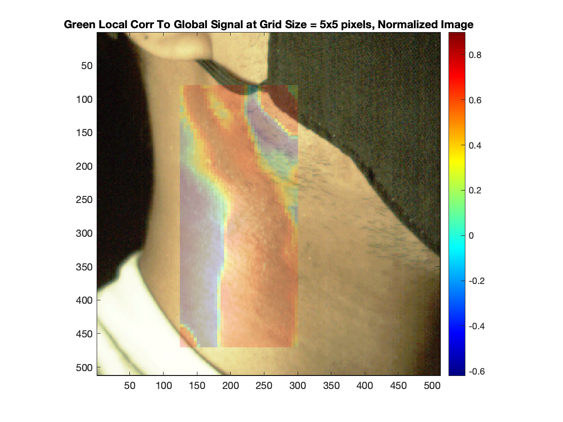 | 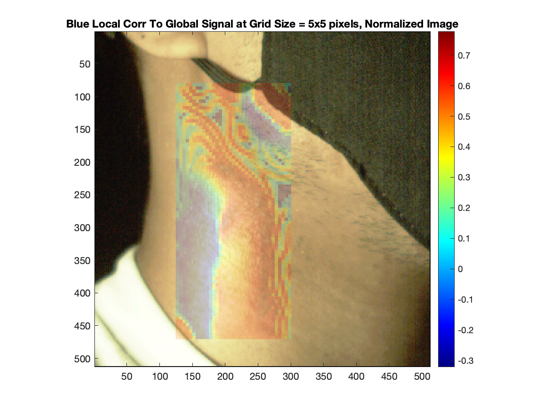 |
| --- | --- | --- |

Figure 35: Correlation of each local SVI (red on left, green in middle, blue on right) to the global SVI signal extracted from the ROI.

| 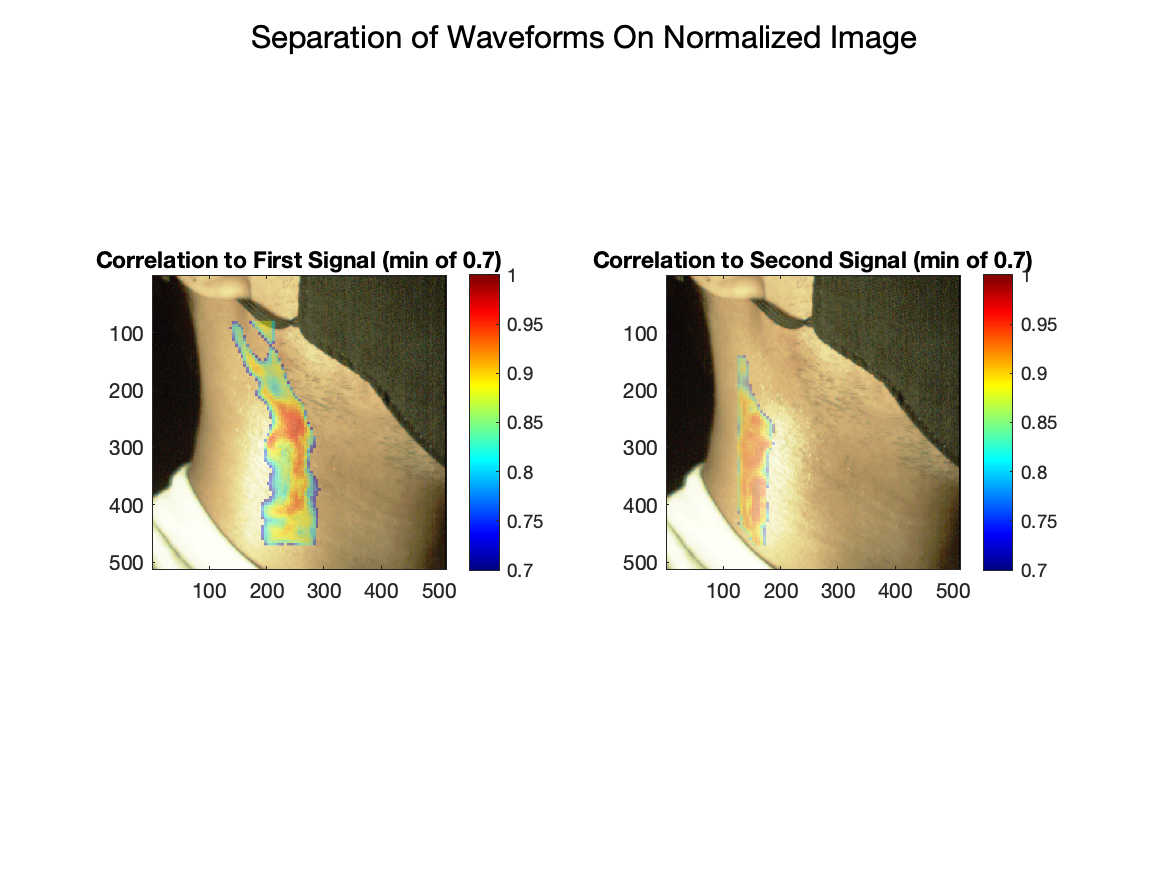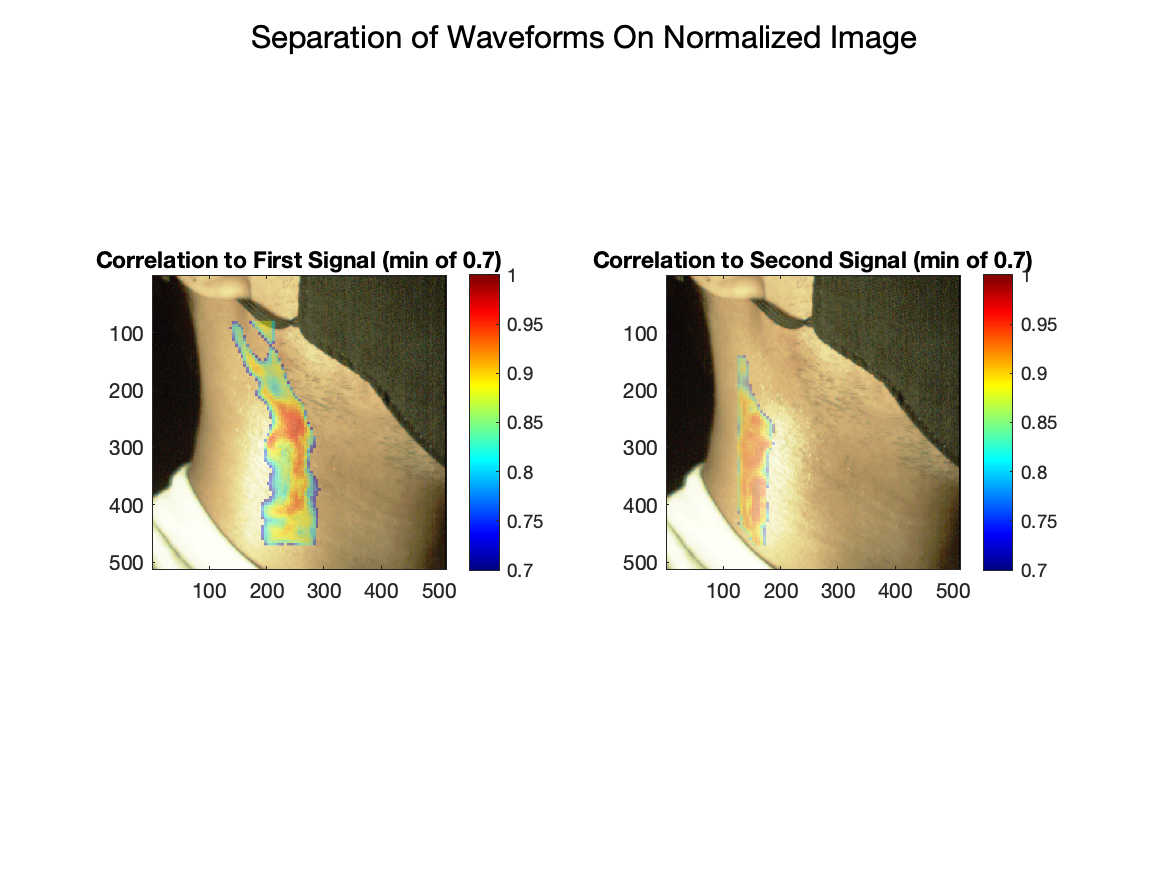 | 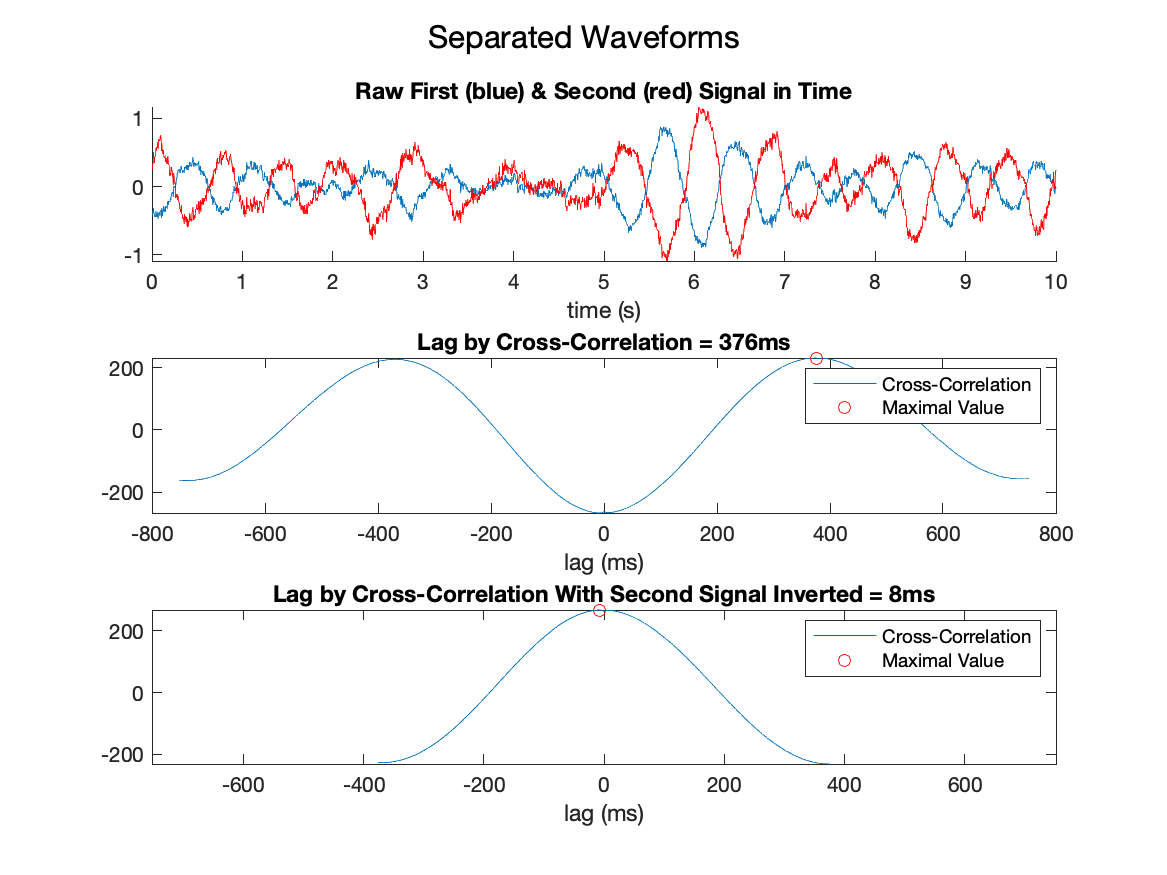 |
| --- | --- |
|  |  |

Figure 36: the first region generating local SVI signals with high internal correlation (top left), the second region generating local SVI signal with high internal correlation (top right), the SVI signals from the first and second regions plotted in the time domain and analyzed with cross-correlation (right).

## Subject 7, Signal B

*n/a – analysis not successful*

## Subject 8, Signal A

| 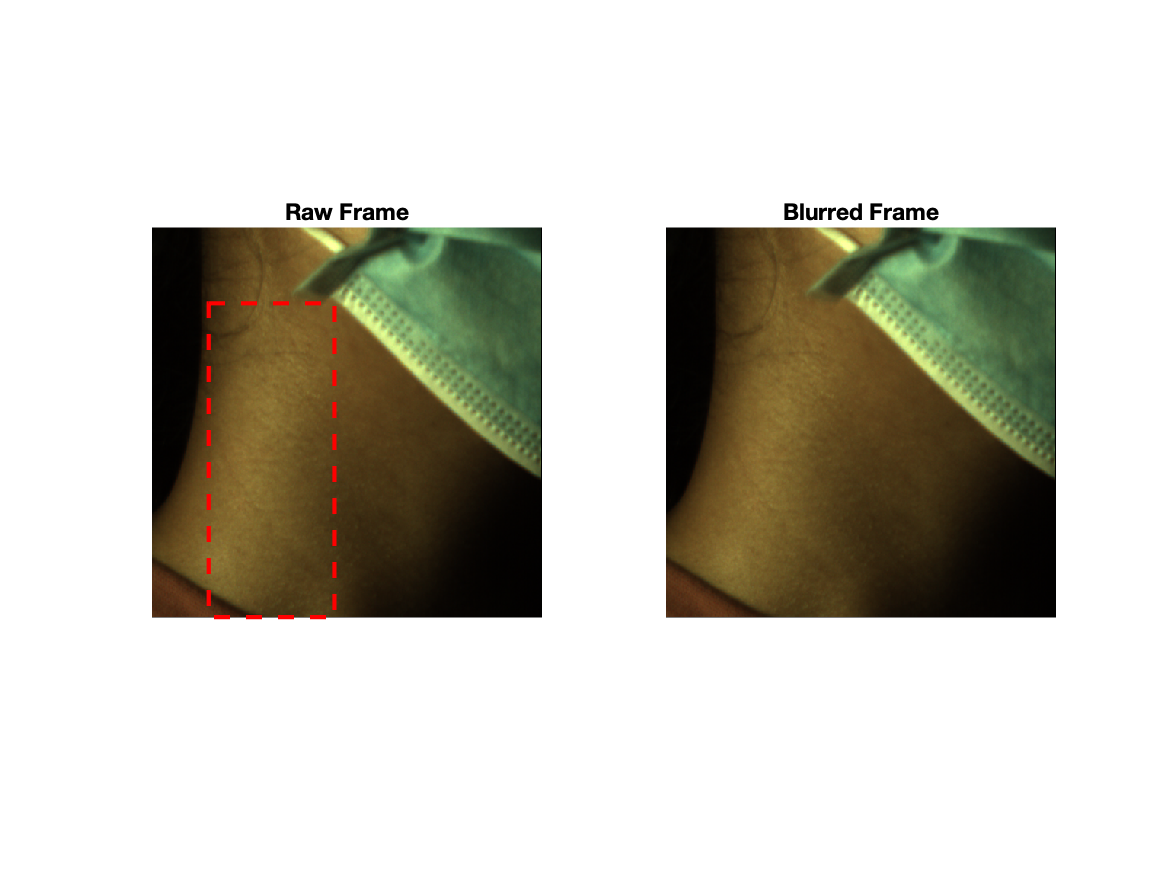 | 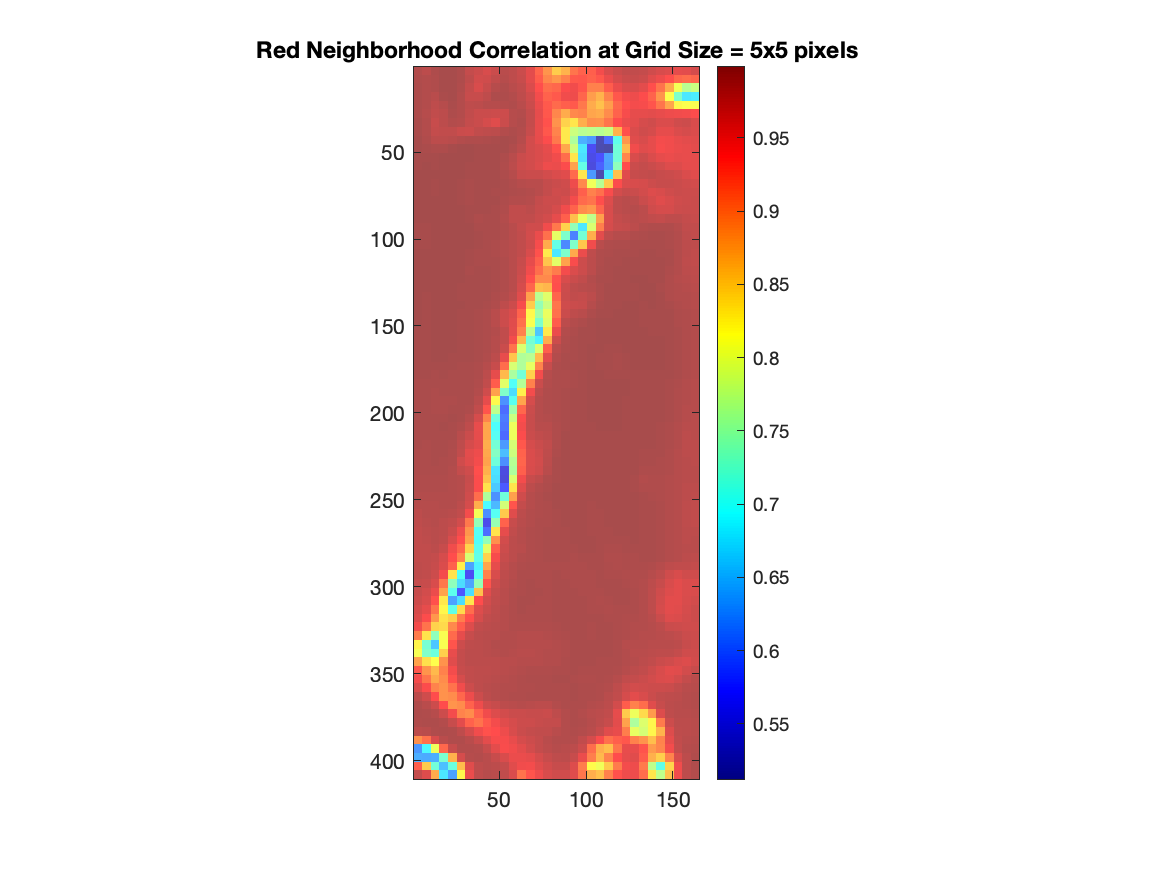 |
| --- | --- |

Figure 37: ROI (left) and local correlation analysis (right)

| 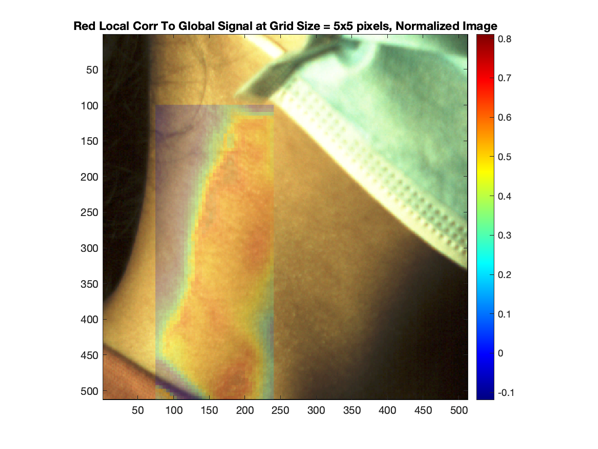 | 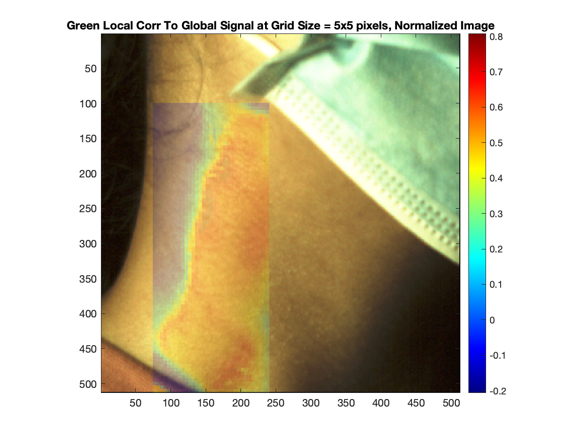 |  |
| --- | --- | --- |

Figure 38: Correlation of each local SVI (red on left, green in middle, blue on right) to the global SVI signal extracted from the ROI.

|  |  |
| --- | --- |
|  |  |

Figure 39: the first region generating local SVI signals with high internal correlation (top left), the second region generating local SVI signal with high internal correlation (top right), the SVI signals from the first and second regions plotted in the time domain and analyzed with cross-correlation (right).

## Subject 8, Signal B

|  |  |
| --- | --- |

Figure 40: ROI (left) and local correlation analysis (right)

|  |  |  |
| --- | --- | --- |

Figure 41: Correlation of each local SVI (red on left, green in middle, blue on right) to the global SVI signal extracted from the ROI.

|  |  |
| --- | --- |
|  |  |

Figure 42: the first region generating local SVI signals with high internal correlation (top left), the second region generating local SVI signal with high internal correlation (top right), the SVI signals from the first and second regions plotted in the time domain and analyzed with cross-correlation (right).

## Subject 9, Signal A

|  |  |
| --- | --- |

Figure 43: ROI (left) and local correlation analysis (right)

|  |  |  |
| --- | --- | --- |

Figure 44: Correlation of each local SVI (red on left, green in middle, blue on right) to the global SVI signal extracted from the ROI.

|  |  |
| --- | --- |
|  |  |

Figure 45: the first region generating local SVI signals with high internal correlation (top left), the second region generating local SVI signal with high internal correlation (top right), the SVI signals from the first and second regions plotted in the time domain and analyzed with cross-correlation (right).

## Subject 9, Signal B

|  |  |
| --- | --- |

Figure 46: ROI (left) and local correlation analysis (right)

|  |  |  |
| --- | --- | --- |

Figure 47: Correlation of each local SVI (red on left, green in middle, blue on right) to the global SVI signal extracted from the ROI.

|  |  |
| --- | --- |
|  |  |

Figure 48: the first region generating local SVI signals with high internal correlation (top left), the second region generating local SVI signal with high internal correlation (top right), the SVI signals from the first and second regions plotted in the time domain and analyzed with cross-correlation (right).

## Subject 10, Signal A

|  |  |
| --- | --- |

Figure 49: ROI (left) and local correlation analysis (right)

|  |  |  |
| --- | --- | --- |

Figure 50: Correlation of each local SVI (red on left, green in middle, blue on right) to the global SVI signal extracted from the ROI.

|  |  |
| --- | --- |
|  |  |

Figure 51: the first region generating local SVI signals with high internal correlation (top left), the second region generating local SVI signal with high internal correlation (top right), the SVI signals from the first and second regions plotted in the time domain and analyzed with cross-correlation (right).

## Subject 10, Signal B

|  |  |
| --- | --- |

Figure 52: ROI (left) and local correlation analysis (right)

|  |  |  |
| --- | --- | --- |

Figure 53: Correlation of each local SVI (red on left, green in middle, blue on right) to the global SVI signal extracted from the ROI.

|  |  |
| --- | --- |
|  |  |

Figure 54: the first region generating local SVI signals with high internal correlation (top left), the second region generating local SVI signal with high internal correlation (top right), the SVI signals from the first and second regions plotted in the time domain and analyzed with cross-correlation (right).
